# Defining Vaginal Community Dynamics: daily microbiome transitions, the role of menstruation, bacteriophages, and bacterial genes

**DOI:** 10.1186/s40168-024-01870-5

**Published:** 2024-08-19

**Authors:** Luisa W. Hugerth, Maria Christine Krog, Kilian Vomstein, Juan Du, Zahra Bashir, Vilde Kaldhusdal, Emma Fransson, Lars Engstrand, Henriette Svarre Nielsen, Ina Schuppe-Koistinen

**Affiliations:** 1 Science for Life Laboratory, Department of Medical Biochemistry and Microbiology, Uppsala University, Husargatan 3, 75237 Uppsala, Sweden; 2 Department of Microbiology, Tumor and Cell Biology (MTC), Centre for Translational Microbiome Research, Karolinska Institutet, Nobels Väg 6, 17177 Stockholm, Sweden; 3 The Recurrent Pregnancy Loss Unit, The Capital Region, Copenhagen University Hospitals, Rigshospitalet and Hvidovre Hospital, Blegdamsvej 9, 2100 Copenhagen and Kettegård Alle 30, 2650 Hvidovre, Denmark; 4 Department of Clinical Immunology, Copenhagen University Hospital, Rigshospitalet, Blegdamsvej 9, 2100 Copenhagen, Denmark; 5 Department of Clinical Medicine, Copenhagen University, Blegdamsvej 3B, 2200 Copenhagen, Denmark; 6 Department of Obstetrics and Gynecology, Copenhagen University Hospital, Hvidovre Hospital, Kettegård Alle 30, 2650 Hvidovre, Denmark; 7 Department of Obstetrics and Gynecology, Region Zealand, Slagelse Hospital, Fælledvej 13, 4200 Slagelse, Denmark; 8 Department of Medicine Solna, Division of Infectious Diseases, Karolinska Institutet, Department of Infectious Diseases, Karolinska University Hospital, Center for Molecular Medicine, Stockholm, Sweden; 9 Department of Women’s and Children’s Health, Uppsala University, Dag Hammarskjölds Vägäg 20, 75185 Uppsala, Sweden

**Keywords:** Vaginal microbiome, Menstrual cycle, Daily variations, Dysbiosis, Reproductive health

## Abstract

**Background:**

The composition of the vaginal microbiota during the menstrual cycle is dynamic, with some women remaining eu- or dysbiotic and others transitioning between these states. What defines these dynamics, and whether these differences are microbiome-intrinsic or mostly driven by the host is unknown. To address this, we characterized 49 healthy, young women by metagenomic sequencing of daily vaginal swabs during a menstrual cycle. We classified the dynamics of the vaginal microbiome and assessed the impact of host behavior as well as microbiome differences at the species, strain, gene, and phage levels.

**Results:**

Based on the daily shifts in community state types (CSTs) during a menstrual cycle, the vaginal microbiome was classified into four Vaginal Community Dynamics (VCDs) and reported in a classification tool, named VALODY: constant eubiotic, constant dysbiotic, menses-related, and unstable dysbiotic. The abundance of bacteria, phages, and bacterial gene content was compared between the four VCDs. Women with different VCDs showed significant differences in relative phage abundance and bacterial composition even when assigned to the same CST. Women with unstable VCDs had higher phage counts and were more likely dominated by *L. iners*. Their *Gardnerella* spp. strains were also more likely to harbor bacteriocin-coding genes.

**Conclusions:**

The VCDs present a novel time series classification that highlights the complexity of varying degrees of vaginal dysbiosis. Knowing the differences in phage gene abundances and the genomic strains present allows a deeper understanding of the initiation and maintenance of permanent dysbiosis. Applying the VCDs to further characterize the different types of microbiome dynamics qualifies the investigation of disease and enables comparisons at individual and population levels. Based on our data, to be able to classify a dysbiotic sample into the accurate VCD, clinicians would need two to three mid-cycle samples and two samples during menses. In the future, it will be important to address whether transient VCDs pose a similar risk profile to persistent dysbiosis with similar clinical outcomes. This framework may aid interdisciplinary translational teams in deciphering the role of the vaginal microbiome in women’s health and reproduction.

Video Abstract

**Supplementary Information:**

The online version contains supplementary material available at 10.1186/s40168-024-01870-5.

## Background

The vaginal microbiota guards the entry of the reproductive tract. In concert with immune cells and the vaginal mucosa, it provides a physical and (bio-)chemical barrier against pathogens, preventing gynecological infections. A healthy vaginal microbiome is dominated by non-pathogenic *Lactobacillus* spp.*,* producing lactic acid, hydrogen peroxide, and bacteriocins, providing an acidic environment and hindering the growth of other bacteria [1]. In some women, the vaginal microbial composition can change suddenly, with a loss of *Lactobacillus* and the growth of other bacteria, often defined as vaginal dysbiosis [2]. A dysbiotic vaginal microbiome is considered “unhealthy” as previous studies have associated it with poor reproductive outcomes, such as prolonged unexplained infertility, preterm birth, sexually transmitted infections (STI), and even gynecological cancers [3–8]. Understanding the dynamics of the intricate interplay between the vaginal microbiota and its environment is crucial for understanding how to maintain or improve women's fertility and reproductive health.

Despite the epidemiological evidence, many unaccounted factors exist in defining a 'healthy' vaginal microbiome. A key issue is that this definition often lacks the temporal aspect: it remains to be determined whether there is a difference in the reproductive health between women with constant vaginal dysbiosis and women with fluctuations between *Lactobacillus* dominance and dysbiosis. *Lactobacillus* dominance can disappear abruptly, with high diversity as a result, but can sometimes be quickly restored. So far, menstruation and sexual activity have been identified as primary drivers of these temporal changes^2^. The pattern of transitions in the vaginal microbiome over time in any individual woman is a complex interaction between three main determinants: inherent causes (genetic, immune system, hormone levels), lifestyle/clinical drivers (sexual intercourse, bleeding, hygiene habits) and microbiome determinants (for example interactions between species or strain-level differences) [9, 10]. How readily a microbiome recovers from dysbiosis may depend on which of these determinants initiates the dysbiosis.

Whether a sudden lack of *Lactobacillus* dominance is due to a loss of *Lactobacillus* spp. that favors the growth of other bacteria or whether other bacteria can suppress the *Lactobacillus* spp. dominance is still a pending question [11–13]. A considerable reduction of lactobacilli that occurs before the expansion of anaerobic bacteria typical of bacterial vaginosis (BV) could be caused by bacteriophages. Lysogenic phages reside inside the bacterial host, and this viral strategy is probably favorable when the density of its host bacteria is low. Notably, phages can rapidly switch from a lysogenic to a lytic cycle, quickly killing the host bacteria and releasing thousands of phage virions [14, 15]. However, only a few studies have focused on the viral players of the vaginal ecosystem. Therein, it was shown that bacteriophages in vaginal swabs, like bacteria, cluster into two unique bacteriophage community groups: a high-diversity and a low-diversity group [16]. These two bacteriophage community groups correlated with the *Lactobacillus* dominance (low diversity bacteriophages) and non-*Lactobacillus* spp. dominance (high diversity bacteriophages) bacterial groups. Moreover, the bacteriophage composition may predict clinical BV as efficiently as the bacteriome composition [17].

The main drivers necessary to induce the overgrowth of certain bacteria, such as *Gardnerella* spp., *Prevotella* spp., or *Fannyhessea vaginae*, into full-blown bacterial vaginosis are still poorly understood. Contributing factors such as biofilm formation, local inflammation, and endocrine differences in the individual may all contribute [18]. While genus *Gardnerella* has long been associated with BV, its recent delineation into 13 genomic species [19] has led to a better understanding of its genomic variability and association with disease [20]. Still, little is known about the pangenome of these species, considering that their genetic variability and accessory genes could be significant in understanding the development of bacterial vaginosis and its role as a risk factor for poor reproductive outcomes and women´s health.

In most studies, determining whether a vaginal microbiome is “unhealthy” is based on its composition on the arbitrary day the sample was taken. Therefore, the definition of an unhealthy vaginal microbiome in women of reproductive age calls for more precise terminology, both in terms of specific patterns of species abundance and in the timing and duration of the dysbiosis in relation to the menstrual cycle or pregnancy. There is a great need for a thorough investigation of the daily transitions in the vaginal microbiome with detailed information on both lifestyle and microbiome features to support future research by defining when and how often a woman needs to be sampled for accurate characterization of her vaginal microbiome.

In the initial phase of this study, 15 women were followed with daily swabs for 42 days. Then, the daily changes in the vaginal microbiome were investigated by shotgun sequencing in an additional 49 young, healthy women during a menstrual cycle to identify potential drivers of sudden transitions in microbiome composition. Based on these data, we aimed to classify the dynamic patterns of the vaginal microbiome composition during a menstrual cycle into Vaginal Community Dynamics (VCDs). The metagenomic approach made it possible to analyze the presence of co-occurring bacteriophages and, by metagenomic assembly, investigate the different bacterial genomic strains that are connected to dysbiosis.

## Results

### Daily vaginal swabs reveal both rapid and cyclic changes in the microbiota

**Fig. 1:**
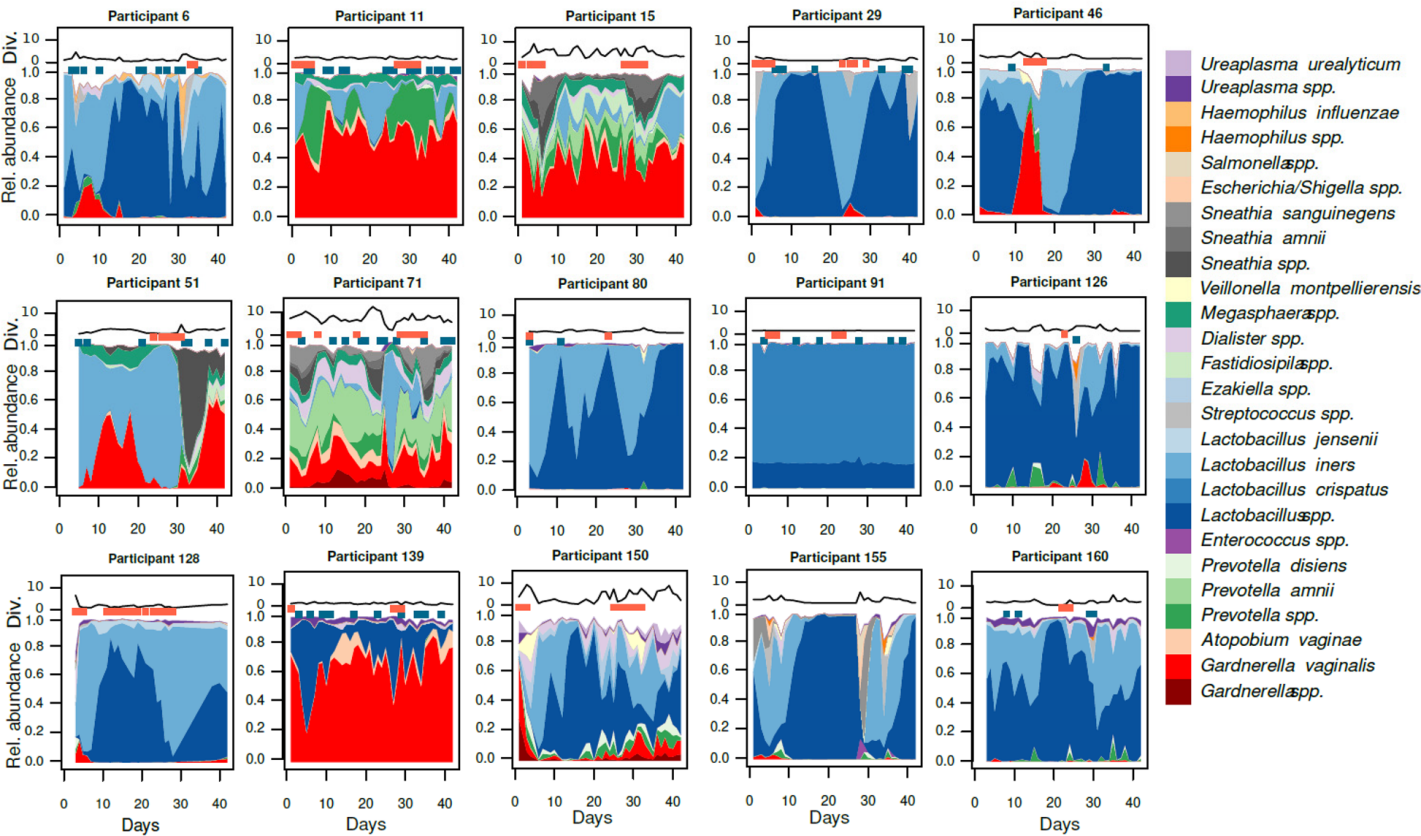
The vaginal microbiota can be remarkably stable over 6 weeks, but also experience both cyclical and rapid shifts. Area plots represent bacterial composition inferred from 16S rRNA gene amplicons, with relative abundance on the *Y*-axis and days on the *X*-axis. Red dots above the area chart represent days with menstrual bleeding or spotting, and the blue dots represent days with vaginal intercourse. The black line above each profile shows their alpha diversity (inverse Simpson’s index)

During the initial stage of the study, the vaginal microbiota of 15 women was analyzed daily for a period of up to 42 days. This analysis involved sequencing the V3–V4 regions of the 16S rRNA gene. These women were using three different contraceptive regimens (non-hormonal contraceptives, NHC; combined oral contraceptives, COC; levonorgestrel intra-uterine system, IUS; *n* = 5 in each group). The samples generated 839 ASV, corresponding to 154 species from 130 genera. The full dataset is presented in Supplementary Table S1. Some women, such as ID 15, 71, and 150, presented significant fluctuations daily, including rapid changes in alpha diversity (Fig. 1). Conversely, participant ID 11 or 139 had essentially constant diversity, and 91 had stable profiles throughout the entire cycle. Despite these fluctuations, we observed the cyclical effect of menses in women with regular menstrual bleeding. Menstruation in participants ID 11, 15, and 29 was repeatedly accompanied by an increase in *Lactobacillus iners, Prevotella* spp., and *Sneathia* spp*.,* respectively. Participant 46 was an example of a reversible change during menses, with a rapid expansion in *Gardnerella* spp., quickly followed by dominance of *Lactobacillus* spp. Conversely, participant 51 showed how changes triggered by menses could become permanent. A significant expansion of *Sneathia amnii* in parallel with the loss of *Lactobacillus iners* resulted in a *Gardnerella vaginalis-*dominated microbiota until the end of the follow-up.

Since this first stage showed the need for daily samples to capture the whole dynamics of the menstrual cycle, additional 49 women were included with daily vaginal swabs from day 4 to day 32 of their menstrual cycle. Metagenomic sequencing was used in the larger sample set to improve taxonomic and functional resolution and extend our analysis to viruses.

### Unprotected intercourse and menstrual bleeding negatively affect the stability of the vaginal microbiome

**Fig. 2:**
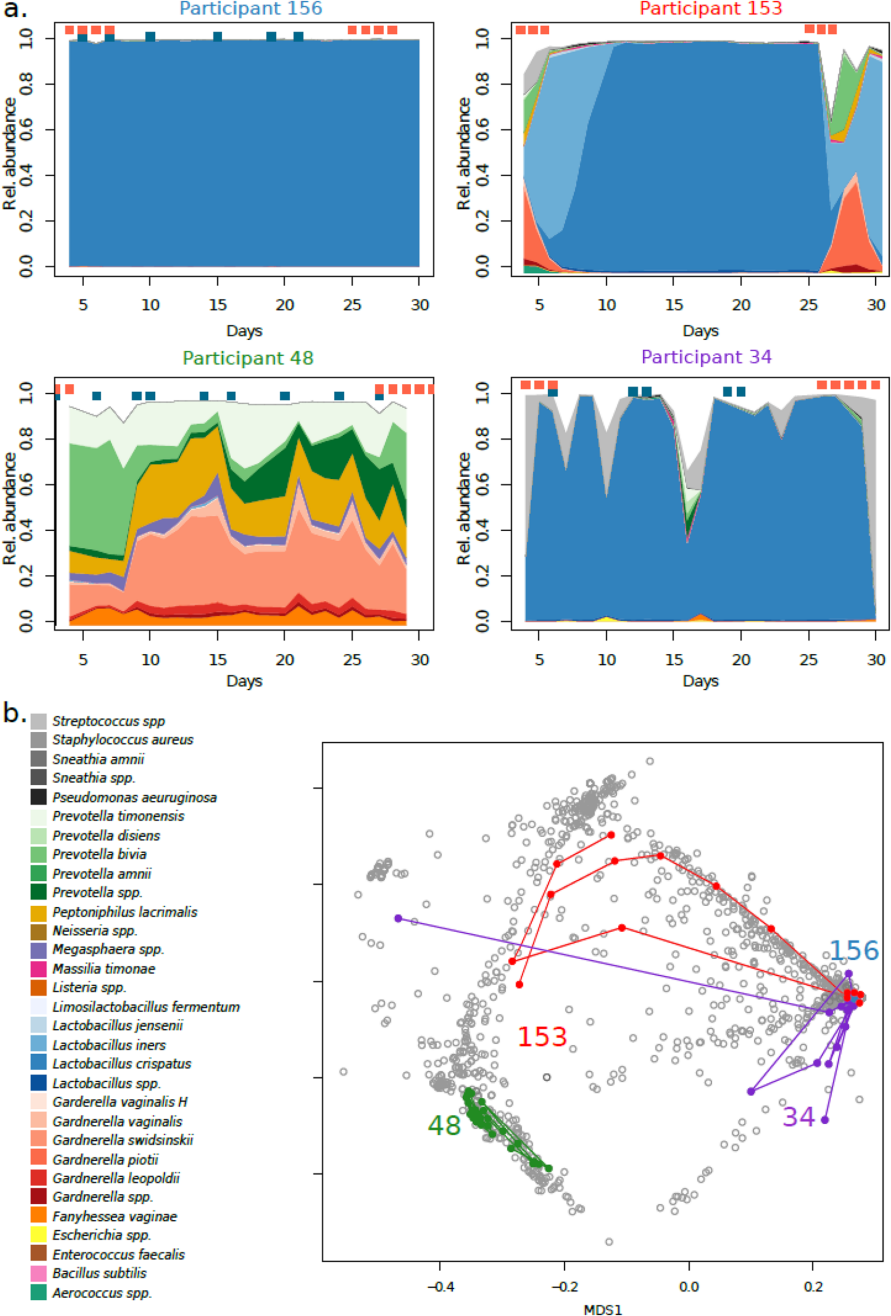
Vaginal samples are dominated by either *Lactobacillus* spp*., Gardnerella* spp., or *Prevotella* spp.*,* and can rapidly or cyclically switch between types. **a** Four representative individuals’ vaginal microbiomes are shown during a menstrual cycle, starting from cycle day 4. Women can be stably high *Lactobacillus* spp*.,* stably *Lactobacillus* spp. depleted, high *Lactobacillus* spp. except during their menses or high *Lactobacillus s*pp*.* but with relative abundances falling as a response to unprotected sexual intercourse. **b** a non-metric multidimensional scaling based on Bray–Curtis dissimilarity of all shotgun metagenomics samples in this study. The same individuals are highlighted, showing their trajectory during the follow-up

For this part of the study, samples from an additional forty-nine women were included using metagenomic shotgun sequencing. An average of 25.9 samples were successfully sequenced, starting from cycle day four with daily samples for another 28 days. The 1269 samples sequenced by shotgun were, like the 532 metabarcoding samples, dominated by *Lactobacillus crispatus, Lactobacillus iners, Gardnerella* spp., and *Prevotella* spp. The total beta diversity during the time series fluctuated considerably in the individual woman. Four representative participants are shown in Fig. 2: The vaginal microbiome of participant 156 was *L. crispatus-*dominated and showed no response to either intercourse or bleeding. Participant 48 had no *Lactobacillus* spp*.* and was instead dominated by *Gardnerella* spp*., Prevotella* spp*.,* and *Peptoniphilus lacrimalis.* The relative abundances of different species of her vaginal microbiome were changing rapidly throughout the study period. Participant 115 was dominated by *Lactobacillus crispatus* in most samples, but during menses, became dominated by *Gardnerella* spp. Finally, participant 34 was *Lactobacillus crispatus* dominated but presented rapid shifts in response to intercourse. All participants are shown in Supplementary Figure S1 and Supplementary Table S2.

**Fig. 3:**
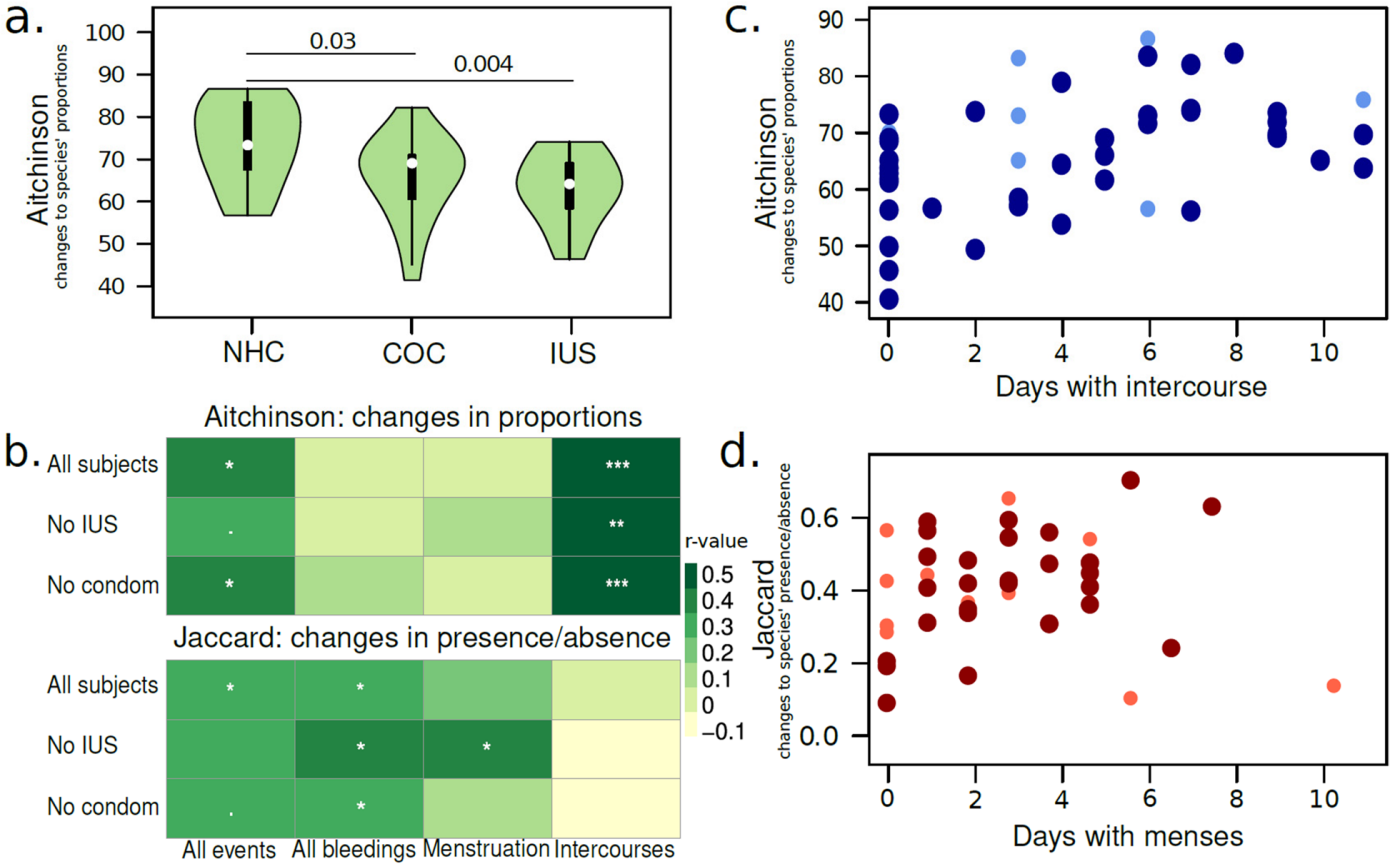
Contraceptive usage and intercourse frequency affect relative abundance of bacteria, while days with menses affect the influx of new bacteria. **a** Women not using hormonal contraceptives had higher total beta-diversity over the sampling period than women on combined oral contraceptives or with an IUS. **b** Pearson’s correlations between life events (bleedings, intercourses) and total beta-diversity, Aitchinson’s or Jaccard’s, per participant in different groups. **c** Number of days with unprotected sexual intercourse was positively correlated to total Aitchinson’s distance. Dark blue dots: women with unprotected sexual intercourse. Light blue dots: women with sexual intercourse with condoms. **d** The number of days with menstrual bleeding was directly correlated to total Jaccard dissimilarity. Dark red dots: women not using hormonal contraceptives or on combined oral contraceptives. Light red dots: women with an IUS, with typically very light bleeding

The stability of the vaginal microbiome may result from features unique to the microbiome or host. We, therefore, started assessing the correlation between total beta diversity and host-specific factors. Changes in microbiome composition can further be divided into two main categories, qualitative (the introduction or removal of species) and quantitative (alterations in the relative abundance of species present), which can be quantified by different metrics. To assess the effect of host variables on microbiome variation, we took the average pairwise sample distance for each participant. Contraceptive usage did not affect qualitative changes in the microbiome (Jaccard distance; all *p* > 0.3). However, Aitchinson’s distance was higher in the non-hormonal contraceptive group than in the combined oral contraceptive or progestin-only intra-uterine system (Fig. 3a). Aitchinson’s and Jaccard’s distances were weakly positively correlated to the total bleeding and spotting days (Fig. 3b). However, only Aitchinson’s was strongly positively correlated with the total number of intercourses, indicating a (temporary) quantitative change in beta-diversity (Fig. 3c). Conversely, only a qualitative change in beta-diversity as measured by Jaccard’s was positively correlated to days with menstrual bleedings after the removal of participants with an IUS (Fig. 3d). Interestingly, we identified eight species that were significantly more prevalent during menses: *Gardnerella vaginalis, Prevotella disiens, Staphylococcus epidermidis, Streptococcus agalactiae, Ureaplasma parvum*, and *Veillonella montpellierensis*. Only *Pseudomonas aeruginosa* and *Massilia timonae* were significantly more prevalent outside of menses. Only *Pseudomonas aeruginosa* and *Massilia timonae* were significantly more prevalent outside of menses, albeit these taxa are often detected in low bacterial load samples and may be spurious. No significant differences in total beta-diversity were found in relation to menstrual hygiene products or douching.

**Fig. 4:**
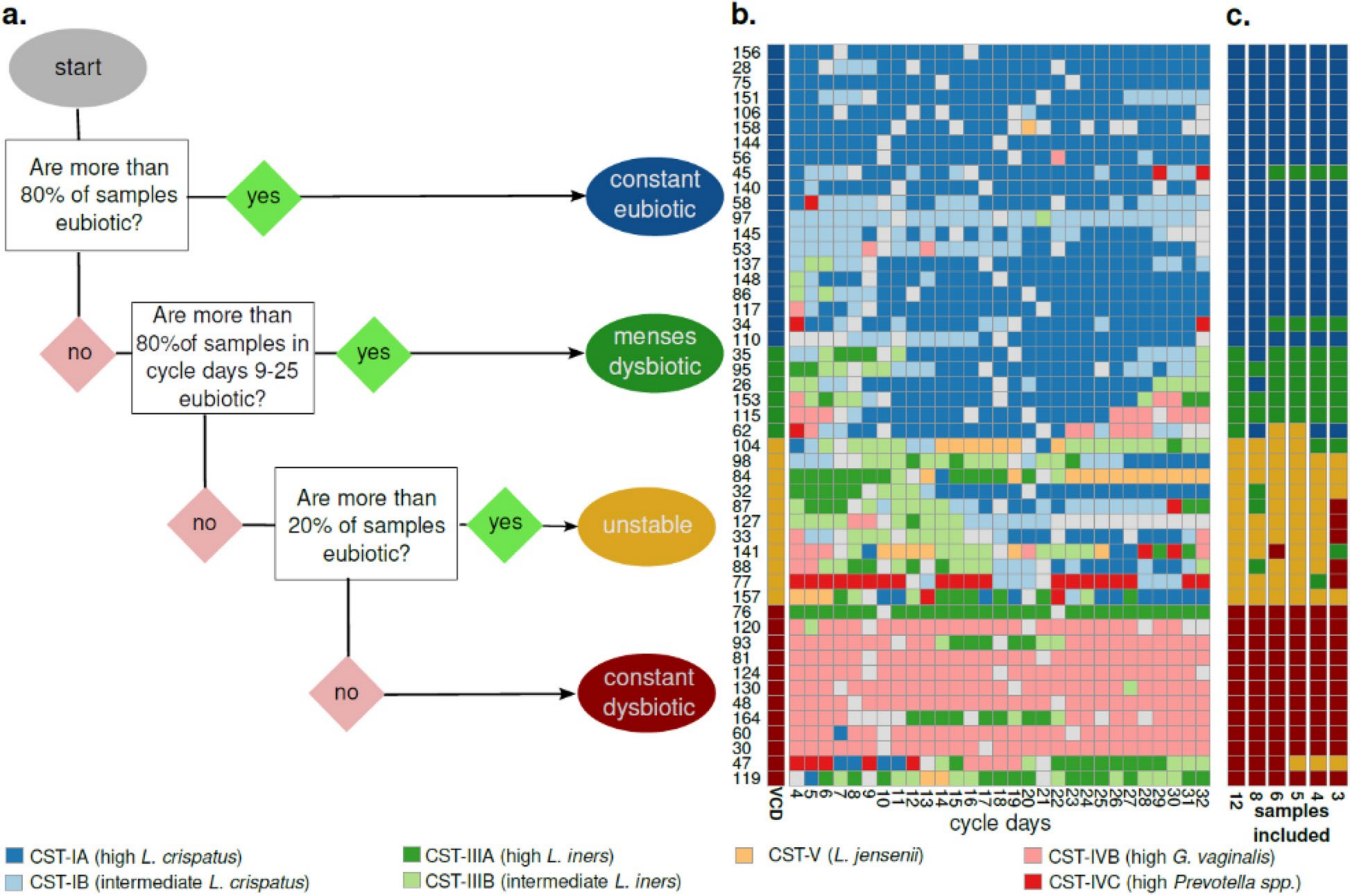
Vaginal time-series can be classified into four categories (Vaginal Community Dynamics) according to their proportion of eubiotic samples. **a** A decision tree can separate a time-series of samples into dynamic groups, based on the community state types. Input from the user is which CST are considered eubiotic (here: I, II, and V) and which days are to be considered free from the influence of menses (here: cycle day 9 to cycle day 25). Time-series with >  = 80% eubiotic samples are considered constant eubiotic; conversely, those with > 80% dysbiotic samples are considered constant dysbiotic. For those in the 20–80% range, a second assessment is done on the days free of menses: if they are > 80% eubiotic, the time-series is considered menses-related dysbiotic, and otherwise unstable (changing from eubiosis to dysbiosis without a clear temporal pattern). **b** A color map with one individual per row and one day per column. The color of each intersection depicts CST. Colored bars on the left side show the vaginal community dynamics of each woman. **c** Additional color bars show the inferred vaginal community dynamics of each participant when using fewer samples for classification

Similar to community state types (CST) becoming a standard for the research community, we have proposed a standard for classifying the dynamics of a vaginal microbiome. We classified vaginal samples during a complete menstrual cycle based on CST into four Vaginal Community Dynamics (VCD). The two first categories consisted of “constant eubiotic” and “constant dysbiotic” if the woman was either eubiotic or dysbiotic in more than 80% of the daily samples during a complete menstrual cycle. Then for the two intermediate VCDs, we assessed the remaining samples' dynamics when samples were not under the influence of menses. If a woman was having > 80% eubiotic samples only during the cycle days 9–25 her vaginal microbiome was classified as "menses-related dysbiotic” and, otherwise, as “unstable” (Fig. 4). In this study, we have defined eubiosis as CST-I and CST-V and dysbiosis as CST-III and CST-IV. CST-II was not observed in this cohort (Fig. 4). These parameters can be adjusted for the specific population studied and the experimental set-up. The code is available from www.github.com/ctmrbio/valody*.* Using this scheme, we found that in 49 women: 20 were constant eubiotic, six had menses-related dysbiosis, 11 were unstable, and 12 were constant dysbiotic (Fig. 4). The CSTs and VCDs for the entire investigated population in this study are shown in Supplementary Figure S2 (16S participants) and Supplementary Figure S3 shotgun participants).

To increase the applicability of the VCDs, we also assessed how many samples during a menstrual cycle are necessary to assign them to the correct VCD accurately. Sampling every other day still gave perfect accuracy. However, separating menses-related dysbiosis from an unstable vaginal microbiome required at least five samples, two during menses and three outside. Meanwhile, constant eubiotic dynamics could be detected with two eubiotic samples, at least one during menses, and constant dysbiosis required 2–3 mid-cycle dysbiotic samples (Fig. 4).

### Characteristics of the metagenomic sequencing study population compared between the four groups of Vaginal Community Dynamics

The women were generally young, lean, and healthy, and the majority had had a single sexual partner in the previous month. Detailed health and demographic information are presented in Table 1, divided into the proposed vaginal time-series microbiota classification presented below.

**Table 1:** Clinical characteristics of the participants in the four VCDs

	Constant eubiotic (*n* = 20)	Menses-related dysbiotic (*n* = 6)	Unstable (*n* = 11)	Constant dysbiotic (*n* = 12)	*p* value
Samples/participant ^1^	25.9 (2.0)	26.1 (1.0)	25.5 (3.1)	26.1 (1.6)	
Total samples	517	157	281	314	
Demographics					
Age (years) ^3^	24.4 (22.4–28.8)	23.2 (21.2–24.0)	23.4 (22.9–24.2)	23.4 (22.6–28.2)	0.55
BMI ^3^	21.8 (21.1–22.9)	22.4 (20.7–22.9)	23.1 (21.7–25.0)	21.8 (20.4–22.3)	0.23
Lifestyle					
Smoking ^2^	0	0	0	0	-
Alcohol					
0–7 units ^2^	15 (75.0%)	5 (83.3%)	10 (90.9%)	11 (91.7%)	0.55
8–14 units ^2^	5 (25.0%)	1 (16.7%)	1 (9.9%)	1 (8.3%)	0.55
Menstrual cycle and hygiene products/habits					
Cycle length (days)	28 (28–30)	28 (28 28)	30 (28 30)	28 (28 28)	
Tampon and/or menstrual cup use ^2^	15 (75.0%)	6 (100%)	9 (81.8%)	10 (83.3%)	0.58
Menstrual pads ^2^	14 (70.0%)	3 (50.0%)	4 (36.4%)	7 (58.3%)	0.33
Douching ^2^	2 (10.0%)	0	2 (18.2%)	2 (16.7%)	0.68
Contraceptives					
Non-hormonal ^2^	9 (45%)	1 (5%)	4 (36.4%)	3 (25%)	0.57
Combined oral contraceptive ^2^	5 (25%)	4 (66.7%)	4 (36.4%)	4 (33.3%)	0.57
IUS ^2^	6 (30%)	1 (16.7%)	3 (27.3%)	5 (41.7%)	0.57
Sexual history					
In a relationship ^2^	11 (55.0%)	2 (33.3%)	5 (45.45%)	9 (75.0%)	0.37
Single ^2^	9 (45.0%)	4 (66.7%)	5 (45.45%)	3 (25.0%)	0.37
Nb. of sexual partners in the last 3 months ^3^	1 (1–1)	1 (0–1)	1 (1–1)	1 (1–2)	0.05 *0.008
Nb. of sexual partners in total ^3^	9 (2–13)	6 (2–16)	7 (4–12)	9 (6–14)	0.75
Average # of vaginal intercourse in the last 3 months ^3^	4 (1–20)	2 (2–31)	6 (1–30)	12 (5–25)	0.80
Women with previous pregnancies ^2^	2 (10.0%)	0	0	1 (8.3%)	0.63
STI/bacterial vaginosis					
Diagnosed with BV, yeast, or STI in lifetime ^2^	9 (45.0%)	2 (33.33%)	3 (27.27%)	7 (63.63%)	0.32
Antibiotic use within the last 3 months ^3^	0	0	0	0	-

Data is presented as ^1^mean (SD), ^2^*n* (%), or ^3^median (IQR)

^2^Fisher’s exact test

^3^Kruskal-Wallis test

^*^Constant eubiotic vs. constant dysbiotic after Bonferroni correction

### Samples from different Vaginal Community Dynamics showed large differences in bacterial content despite having the same CST

One factor that can affect microbiome communities’ stability is bacterial species that perform critical ecological services despite being in low abundance. To assess whether this was likely the case for our samples, we used ANCOM-BC2 to assess whether samples from different VCDs differed in their bacterial composition. Because the VCDs are based on CST proportions, we only compared samples within the same CST subtype. Additionally, in each comparison, we only included dynamic groups with at least ten samples from at least three different women to minimize the effect of individual outliers, and adjusted for the participant ID as a random effect. This way, we could compare the menses-dysbiotic and unstable groups to constant eubiotic in CST-IA and CST-IB and constant dysbiotic in CST-IIIA, CST-IIIB, and CST-IVB.

**Fig. 5:**
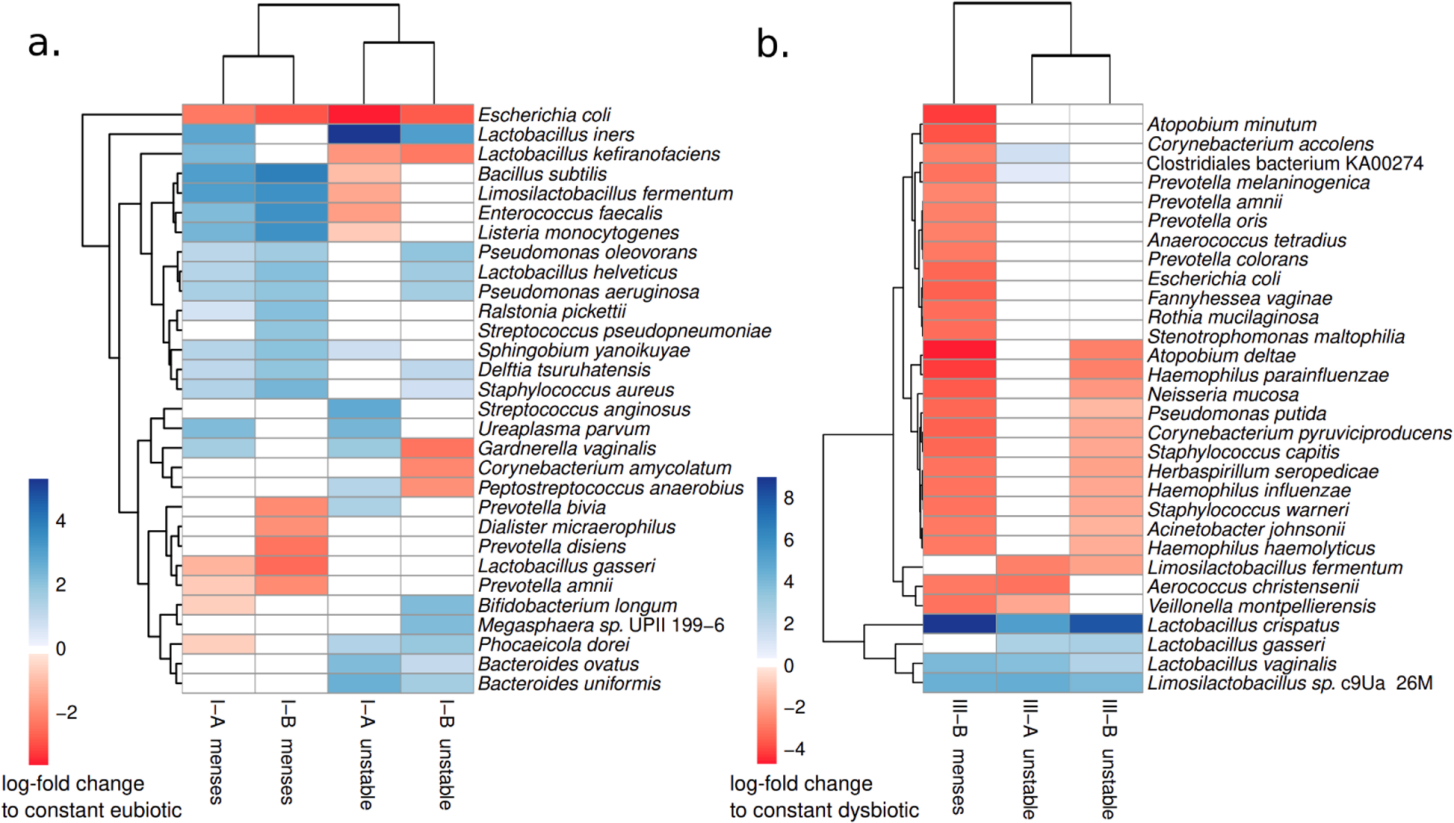
Samples belonging to the same CST, but deriving from different dynamic groups, have changes in the relative abundance of several bacterial species. **a** Samples in CST-IA and CST-IB from menses-related dysbiotic or unstable individuals were compared to those from stable eubiotic individuals. **b** Samples in CST-IIIA and CST-IIIB from menses-related dysbiotic or unstable individuals were compared to those from stable dysbiotic individuals. In each panel, the heatmap shows the log-fold change of the top 30 most extreme differences. White fields represent no significant change

Regarding the highly eubiotic CST-IA, samples from menses-dysbiotic and unstable participants have more *L. iners, Gardnerella* spp., and *Ureaplasma urealyticum* than constant eubiotic individuals. The unstable individuals also had an increased abundance of *Campylobacter* spp*., Corynebacterium* spp*.,* and *Streptococcus* spp. Interestingly, the eubiotic samples had two-fold higher *E. coli* than both the unstable and the menses-dysbiotic samples. In CST-IB, the results were similar, albeit the non-stable time series had an even lower abundance of certain BV-associated bacteria. Specifically, samples from menses-related dysbiotic women had a lower abundance of *P. amnii, P. bivia*, and *P. disiens,* while the unstable time series had a lower abundance of *Fannyhessea vaginae* and *Corynebacterium* spp., in addition to decreased *E. coli.* The top 30 most extreme fold-changes are shown in Fig. 5a (for the complete results, see Supplementary Figure S4 and Supplementary Tables S3 and S4).

Focusing on CST-III, the unstable and menses-dysbiotic participants had a higher relative abundance of several *Lactobacillus* species than the constant dysbiotic, most notably *L. crispatus* and *L. gasseri*, *L. vaginalis, L. reuteri*, *and Limosilactobacillus* spp. Additionally, in CST-IIIB, the menses-related dysbiotic individuals had decreased abundance of several *Prevotella spp, Veillonela spp, Megasphaera* spp*., and Mobiluncus* spp. and the BV-associated Clostridiales KA00067. The top 30 extreme fold-changes are shown in Fig. 5b (complete in Supplementary Figure S4 and Supplementary Tables S5 and S6). For comparison, too few menses-dysbiotic samples were assigned CST-IIIA. No significant results were found comparing samples in CST-IVB.

As a complementary analysis to the one above, we also assessed differentially abundant bacteria between dynamic groups not separated by CST, using either the constant eubiotic or the constant dysbiotic as the baseline for comparison. Both menses-dysbiotic and unstable had more differentially abundant bacteria compared to the constant dysbiotic time-series than when compared to eubiotic (menses-dysbiotic: 41 from constant eubiotic, 136 from constant dysbiotic; unstable: 81 from constant eubiotic, 128 from constant dysbiotic). In common, both intermediate groups have more *L. iners,* more *S. agalactiae* and more *Ureaplasma parvum* than the constant eubiotic (Supplementary Figure S6; Supplementary Table S7). Conversely, compared to the constant dysbiotic, the intermediate VCDs have decreased relative abundance of both *Prevotella* spp*.* and BV-associated bacteria, but an increase in *Bifidobacterium* spp*., Lactobacillus* spp*., Staphylococcus* spp*.* and *Streptococcus* spp. (Supplementary Figure S6; Supplementary Table S8)*.* While roughly confirming what was expected, these analyses were not significant when adjusted for either individual ID or sample CST and must therefore be interpreted with caution.

### Common and abundant species display considerable differences in functional gene content, but no association with microbiome stability

**Fig. 6:**
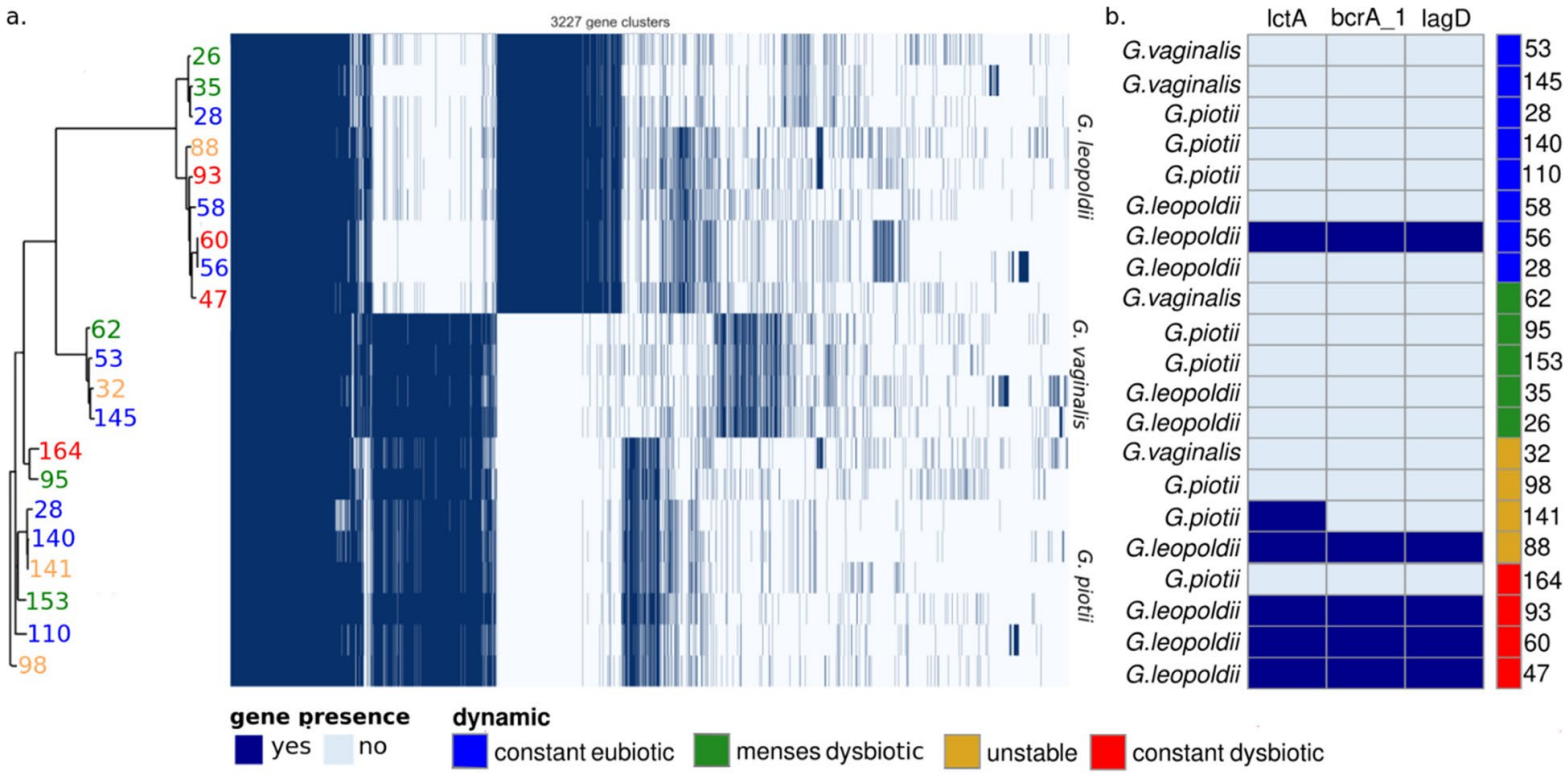
While strains do not segregate by vaginal community dynamics, bacteriocins are associated with instability. **a** Phylogenomic analysis of all detected *Gardnerella* species did not find a correlation between the individuals’ vaginal community dynamics and the observed phylogeny. Each row represents a genome and each column is a gene cluster. **b** Three bacteriocins from *G. leopoldii* were over-represented in unstable and dysbiotic samples. The presence of a gene is represented in dark blue and its absence in light blue. Participants are colored after their VCD. Blue: women who are constantly eubiotic. Red: women who are constantly dysbiotic. Yellow: women with unstable VCD. Green: women who are menses-related dysbiotic

In addition to bacteria in low abundance, it is possible that what determines the dynamics of a community are specific strains and their accessory functional genes. The time-series design of this study also allowed us to build draft genomes (MAGs, metagenome-assembled genomes) for prevalent and abundant bacteria within each participant. At the read-level analysis, all *Gardnerella* spp. reads were classified as *G. vaginalis.* However, genome assembly revealed at least three species in this small cohort, namely *G. leopoldii*, *G.piotii*, and *G. vaginalis*, of which the latter is the least common. There was however no difference between the occurrence of these species in the different VCD (Fig. 6a). The taxonomic identity of *Gardnerella* species from reads was therefore reassessed through direct mapping to these genomes, as specified in the methods.

To assess strain-level differences, we focused on the genera which yielded the largest number of high-quality MAG, i.e. *Gardnerella* spp.*, Lactobacillus* spp., and *Prevotella* spp. The three *Gardnerella* species had a relatively small pangenome, unlike the large *Prevotella* pangenomes*.* Among *Lactobacillus* spp*., L. crispatus* had by far the largest pangenome, while *L. iners* had the smallest (Supplementary Figure S7). Most gene families were classified as either “core” (present in all or almost all genomes) or “cloud” (present in one or very few genomes), with fewer clusters in the intermediate “shell” category. Because of this distribution pattern, no gene cluster could be significantly associated with a VCD (Supplementary Figures S8–S9; Tables S9–S11).

Still, while not significant past multiple testing corrections, we did find three bacteriocin genes associated with *G. leopoldii* that were over-represented in unstable or constant dysbiotic participants (Fig. 6b).

### Bacteriophages stabilize vaginal microbiomes, both in eubiosis and dysbiosis

**Fig. 7:**
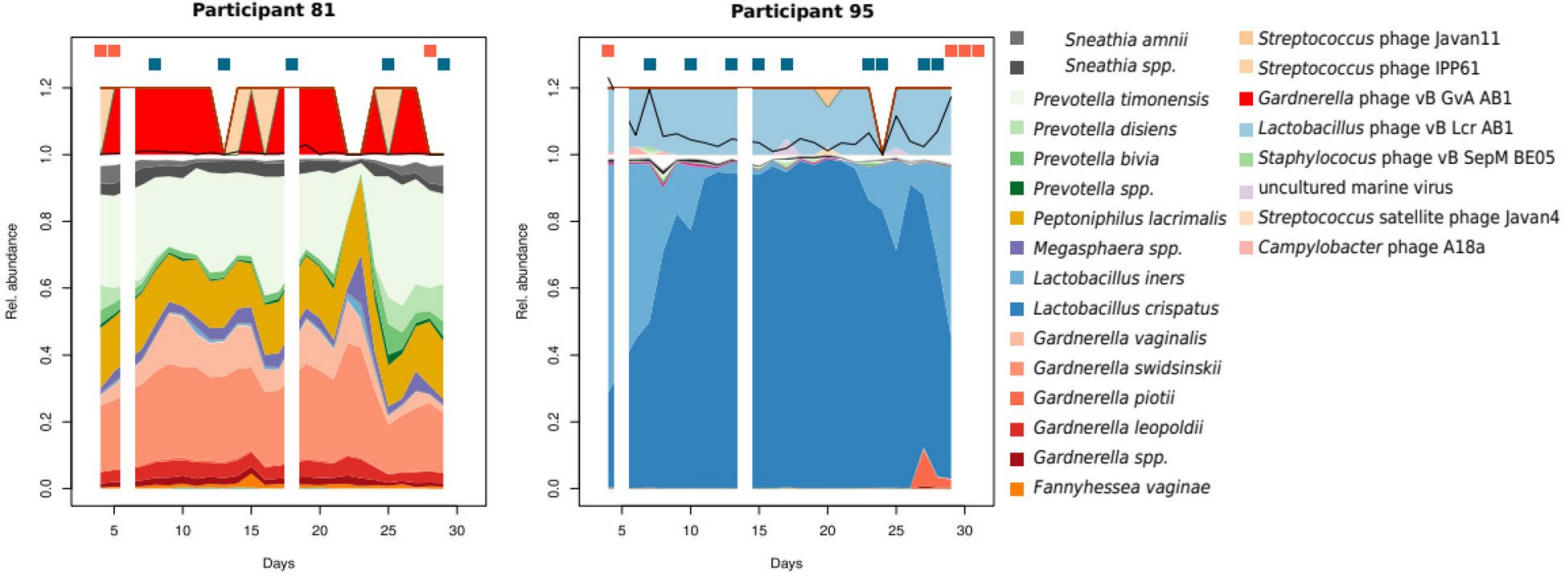
Phage profiles follow roughly the bacterial profiles but can fall below the detection limit in samples of lower coverage. Two representative individuals’ vaginal bacteriomes and phageomes are shown during a menstrual cycle, starting from cycle day 4. The red dots above the area chart represent days with menstrual bleeding or spotting, and the blue dots represent days with vaginal intercourse. The black line overlapped with the phage profiles represents the ratio between phage reads and bacterial reads. Days with missing data are omitted

Bacteriophages are also known to affect the stability of bacterial communities directly, sometimes very rapidly. In this cohort, the taxonomic profile of phages mostly followed their host bacteria (Fig. 7). This was expected, since samples were not enriched for viral particles, so presumably, most viral DNA originates from internalized or integrated phages. Still, we also detected a variety of phages associated with species more typical of the skin, such as *Streptococcus* spp*., Staphylococcus* spp., and *Propionibacterium* spp*.* (Supplementary Figure S1).

The ratio of phage read counts to bacterial read counts was highly variable, ranging from the detection limit of c. 10E − 05 up to 2.7%. Phage counts typically spiked when samples presented another CST than the most prevalent for that subject. Samples with higher phage ratios had also a larger distance to their previous and following samples, with Jaccard’s and Aitchinson’s distances (Spearman’s rank correlation: Aitchinson’s, rho = 0.09, *p* = 0.02; Jaccard, rho = 0.13, *p* = 0.0002).

**Fig. 8:**
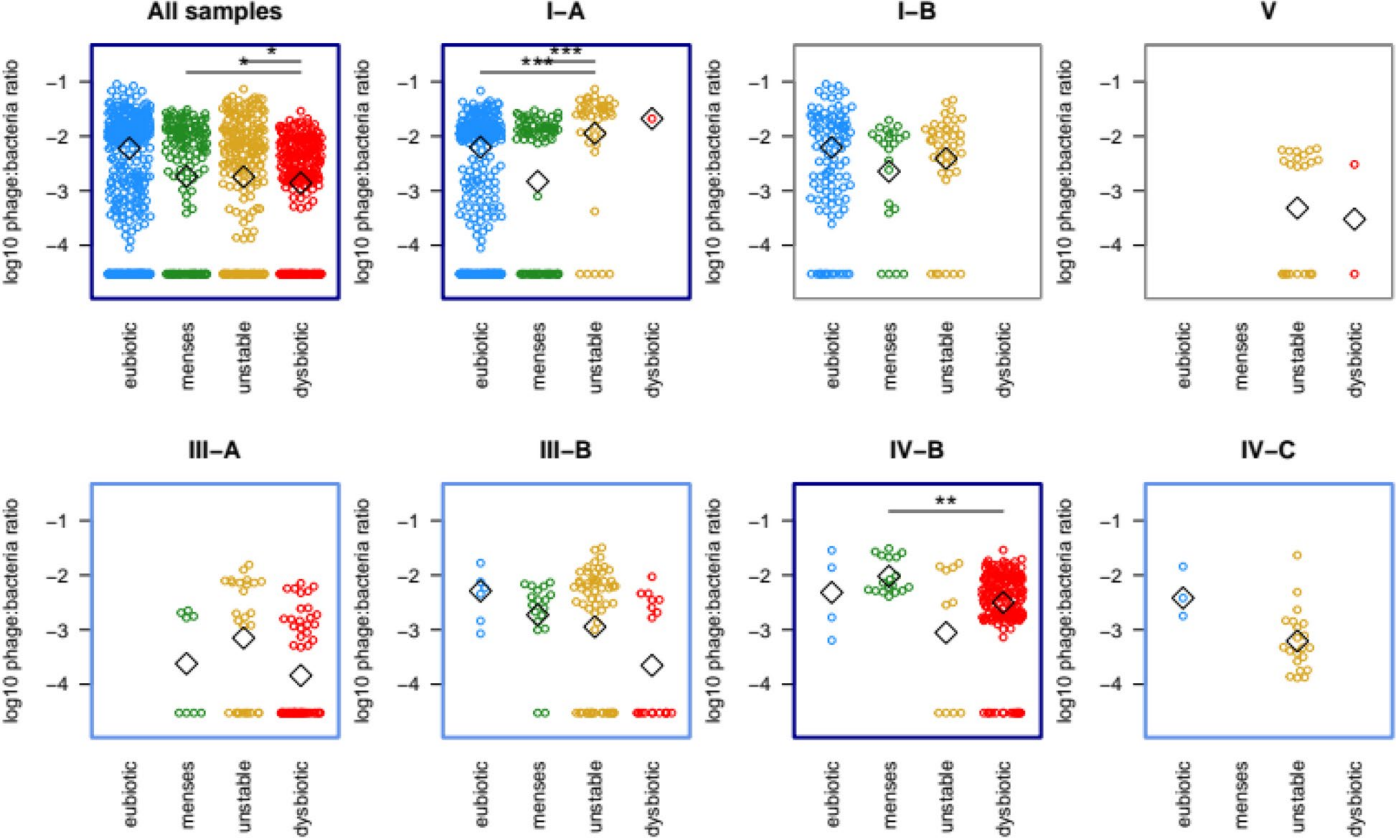
Relative abundance of phages is connected to CST and vaginal community dynamics. The *Y*-axis in each plot represents log10 of the ratio between phage reads and bacterial reads. The *X*-axis represents the VCDs and the top indicates the CSTs. Each open circle is a sample, open diamonds are medians. Results that are significant in Welch’s test are framed in blue, with dark blue marking those that are also significant when adjusting for participant ID. For these only, the results significant on a post-hoc test are marked with stars. *p < 0.05; **p < 0.01; ***p < 0.001

Phage ratios were also significantly different between VCD and, within them, CST. The constant eubiotic samples had the highest phage counts overall, c. tenfold higher than all others. Within CST-I and CST-III, the unstable and dysbiotic VCD had the highest phage counts, while menses-related dysbiotic only had a higher phage ratio in CST IV-B. Only the findings for all samples, CST-IA and CST-IVB were robust to adjusting for participant ID (Fig. 8).

## Discussion

The main aim of this study was to identify and map the dynamics of the vaginal microbiome composition in healthy, young, Caucasian women during the entire menstrual cycle. Several groups have tried to define types of vaginal compositions in women, only to ascertain an extreme variation between individuals [2, 21–24]. The initial phase of our study stressed the need for daily sampling but also indicated that a single menstrual cycle could be sufficient. One of the pivotal keys when refining our understanding of the categorization of the vaginal microbiome is to evaluate the composition dynamics during the menstrual cycle in each woman. As shown in this study, the vaginal microbiome composition can change rapidly, from day to day, and with marked alterations primarily driven by external factors such as menstrual bleeding or sexual intercourse, with both exposures also reported in previous studies [2, 23]. In contrast to previous works, we tried to identify the difference between women who constantly remain eubiotic or dysbiotic during an entire menstrual cycle. Since previous work did not fully account for the dynamic patterns in vaginal microbiome composition, it is unknown whether women with transient (menses-related or unstable) dysbiosis present a lower risk of reproductive health complications than women who are constantly eubiotic. The transient dysbiotic phenotype could reflect a stepwise increase in severity, with menses-related and unstable dysbiosis being potential precursors of eventually transiting into unhealthy dysbiosis. This study defined four Vaginal Community Dynamics (VCDs): (1) the constant eubiotic with non-*iners Lactobacillus* spp. dominance throughout the cycle and an apparent resilience against exposures, (2) the menses-related dysbiotic with a sudden drop in *Lactobacillus* spp. dominance during menstruation, (3) the unstable dysbiotic which changes community states for a short while, for example, after sexual intercourse, and reinstate *Lactobacillus* spp. dominance, (4) the constant dysbiotic, characterized by an overrepresentation of typical BV-associated bacteria such as *Gardnerella* spp., *Prevotella* spp., and *Fannyhessea vaginae* throughout the menstrual cycle. Women with menses-related dysbiosis present a qualitative change in their microbiome composition during menses, with the introduction and removal of several species. In this period, there is an overabundance of *Gardnerella* spp. or *L. iners, Prevotella* spp*.,* and *Sneathia* spp., with conversion back to *L. crispatus* dominance mid-cycle. Alterations in the mucosa could be driving this pattern, perhaps caused by decreasing estradiol levels. However, we also see a direct correlation between the number of days with menstrual bleeding and Jaccard’s distance, possibly implying a role of menstrual blood as a source of bacterial nutrients and increasing the vaginal pH value. Another interesting observation in this study is that the expansion of *Sneathia* spp. was almost only observed immediately after menstrual bleeding. *Sneathia* spp. are opportunistic pathogens that have been associated with adverse pregnancy outcomes such as premature rupture of membranes, preterm birth, and chorioamnionitis [25]. It was recently found that *Sneathia amnii* produces an exotoxin that can haemolyze erythrocytes and break down cellular barriers [26]. Additionally, the higher level of phages in the dysbiotic samples (CST-IVB) observed in women classified as menses-related VCD could contribute to the rapid eradication of BV-associated bacteria after menses. Due to methodological constraints, our analysis of phages focused on their abundance rather than their taxonomy. Nevertheless, these findings show that vaginal bacteriophages are linked to the bacterial community’s stability, consistent with previous research on bacteriophages and the vaginal microbiota [17, 27].

Women with unstable VCD may be particularly susceptible to pH changes, allowing a temporary overgrowth of anaerobic bacteria. Alternatively, this could be explained by a direct introduction of non-vaginal species by their sexual partner. Both scenarios would explain a strong correlation between total beta-diversity (measured as Aitchinson’s distance) and the number of intercourses. Moreover, the unstable group showed a higher prevalence of *L. iners*, which is associated with the formation of vaginal biofilms in women with bacterial vaginosis (BV) [28]. This biofilm could make the affected women more prone to an unstable vaginal microbiome, eventually leading to constant vaginal dysbiosis. Women classified as unstable VCD also had a higher ratio of phage DNA in their metagenomes in CST-IA and CST-III. If these phages can enter a lytic cycle under changing environmental conditions, they could contribute to a kill-the-winner dynamic, where neither *L. crispatus* nor *L. iners* can dominate the microbiome before having their population killed by phages [29]. Further research is needed to explore the role of bacteriophages in the development, maintenance, and recovery from dysbiosis.

The constant dysbiotic VCD is characterized by a highly diverse composition with many aberrant bacteria throughout the menstrual cycle. The critical difference between persisting dysbiosis and the two VCDs with a varying degree of dysbiosis (unstable and menses-related) could be the establishment of a resistant polymicrobial biofilm in the former. The biofilm can consume nutrients, produce metabolic waste products, and create an altered microenvironment that is more permissive to the growth of harmful bacteria [30]. At the same time, it hinders the re-colonization by *Lactobacillus* spp. Indeed, we found that both menses-related dysbiotic and unstable time series had more *Lactobacillus crispatus* and other non-*iners* species in their CST-III and CST-IV samples than the women classified as constant dysbiotic VCD.

We hypothesized that specific genomic strains of species might be more prone to promote either permanent dysbiosis or favor a stable *Lactobacillus* spp. dominance with a special focus on the prevalent and abundant genera *Gardnerella, Prevotella,* and *Lactobacillus.* The metagenomic assembly of the pangenomes revealed that despite all *Gardnerella* sequencing reads initially being assigned to be *G. vaginalis,* this species was the least prevalent genomic *Gardnerella* spp, which prompted our reassignment of these reads by mapping, as described in the methods. Some studies have suggested that the production of bacteriocins by *Gardnerella* spp. may contribute to the pathogenesis of BV by inhibiting the growth of lactobacilli spp. and other beneficial bacteria in the vaginal microbiome. *Gardnerella* spp. can produce antagonistic substances against eubiotic indicator strains in BV conditions [31, 32]. In this study, bacteriocin-like genes in *G. leopoldii*, such as lctA and lagD, affect Gram-positive bacteria cytoplasmic membranes and inhibit cell wall synthesis, resulting in a broad action spectrum in the vaginal niche [33, 34]. As a result, this could create an environment conducive to the overgrowth of pathogenic bacteria and dysbiosis. Indeed, we identified three bacteriocins produced by *G. leopoldii,* which were almost exclusive to the VCDs constant dysbiotic and unstable, supporting a role for bacteriocins in maintaining dysbiosis.

The new time series classification presented here extends the complexity of vaginal dysbiosis and provides a more nuanced characterization of the vaginal ecological system. This framework may aid interdisciplinary translational teams working to improve reproductive outcomes. Further research is needed to identify markers of VCDs that could help reduce the need for consecutive sampling. Based on the data presented here, the clinicians need to sample two or three times mid-cycle and two times during menses to be able to classify a dysbiotic sample into the accurate VCD.

We acknowledge that while our classification approach represents an advancement in understanding temporal trajectories of vaginal microbiota, it is an initial step rather than a definitive clustering. The classification of VCDs is grounded in observed patterns within our dataset, which provides valuable insights into the dynamics of vaginal microbiota. However, like all novel frameworks, it requires further validation and refinement through larger, more diverse studies to capture the full spectrum of vaginal microbiota dynamics. This initial classification serves as a robust foundation for future research, aiming to develop more precise and comprehensive classification methods that can be reliably applied in clinical settings.

## Conclusion

Women have been excluded from clinical trials primarily due to the complexity related to the menstrual cycle changes. This approach has left a black box in medicine that will require substantial focus to overcome. Indeed, this study confirms the complexity related to the cycle and based on the data presented here we propose four Vaginal Community Dynamics based on the specific dynamics of the bacterial composition over time. This categorization of the time series of vaginal samples enables comparisons at individual and population levels. It will assist in identifying the drivers behind the different dynamic profiles of the vaginal microbiome by gaining a better ecological understanding of the role of *Lactobacillus* spp. and their interaction with the host and other components of the vaginal microbiota. To further understand why some women are resilient to exposures such as menses and intercourse while others are not, there is a need for detailed research on the bacterial, fungal, and viral populations of the four dynamic categories. These findings could develop into both prevention and rescue strategies against bacterial vaginosis. Future research should also assess whether transient dysbiosis, either menses-related or unstable, presents a risk profile similar to either constant eubiosis or dysbiosis.

## Methods

### Participants and samples

The participants of this study were recruited for the MiMens study, aiming to understand the interplay between hormonal contraceptives, the menstrual cycle, and the human microbiome [24, 35]. The samples analyzed in this study were collected at home by the participants daily for 42 days, starting on the first day of menses. Women without regular menses started their sampling on a random day (participants 6, 46, 91, 126, 160 in the 16S analysis and 56, 60, 84, 144 in the shotgun analysis). Samples were collected with a FLOQSwab (COPAN diagnostics) and preserved in DNA/RNA-shield (Zymo Research) in the participant's house until the end of the 42 days when they were taken to the clinic and frozen at − 80 °C. For the first part of this paper, 15 participants were selected, five each on combined oral contraceptives (COC), levonogestrel-intrauterine system (LNG-IUS), or not using hormonal contraceptives (NHC), and all samples were analyzed. For the second part, an additional 49 participants were selected from the MiMens cohort, 15–17 from each contraceptive group. The included participants had collected a minimum of 22 out of 25 samples from cycle day 4 and 28 days onwards (cycle day 32 or early in the next cycle) and preference was given to those with regular menses.

### DNA extraction, library preparation, and sequencing

DNA was extracted with a Quick-DNA Magbead Plus kit, as previously described [36]. Libraries of 16S rRNA gene fragments were based on the V3–V4 region, using 80 ng of input DNA and primers 341f-805r [37] prepared with a construct containing Illumina adapters and double barcodes: 341f 5′-CAA GCA GAA GAC GGC ATA CGA GAT N8 GTC TCG TGG GCT CGG AGA TGT GTA TAA GAG ACA GGA CTA CHV GGG TAT CTA ATC C-3′ and 805r 5′-AAT GAT ACG GCG ACC ACC GAG ATC N8 TCG TCG GCA GCG TCA GAT GTG TAT AAG AGA CAG CCT ACG GGN GGC WGC AG-3′, where N8 represents an eight bp long barcode. PCR was conducted for 25 cycles of 98 °C for denaturation, 53 °C for annealing, and 72 °C for the extension. Shotgun metagenomic libraries were prepared with the MGI FS DNA library prep kit (MGI, Shenzhen, China) with the alterations described in [36] and sequenced on a DNBSEQ-G400 sequencer (MGI) using the high-throughput sequencing set (PE150 1000016952; MGI) with DNA libraries loaded onto the flow cell using the DNB loader MGIDL-200 (MGI).

### Taxonomic annotation

16S rRNA gene amplicons were processed and annotated with the DADA2 pipeline [38] with the following settings: max_n = 0, trunc_q = 2, max_ee = 2, trunc_len_fwd = 274, trunc_len_rev = 250, min_overlap = 30, max_mismatch = 0, –min_lencutoff = 380. Taxonomy was based on the SILVA 128 database [39] with the assign_taxonomy and add_species functions, with multi_species set to True.

Shotgun libraries were annotated by mapping to the OptiVag DB v2 with kraken2 [40], as previously described^36^. The taxonomic tables, both 16S-based and shotgun-based, were filtered using decontam on the prevalence mode [41]. This resulted in the removal of 17 16S ASV and 5 metagenomic species. Since kraken2 could not differentiate *Gardnerella* strains, we prepared a reference library containing all Gardnerella genomes in GTDB that represent a unique genomospecies (i.e., GCF_003293675.1, GCF_003397585.1, GCF_003397705.1, GCF_001042655.1, GCF_001563665.1, GCF_001546455.1, GCF_000263635.1, GCF_001546485.1, GCF_000263595.1, GCF_002896555.1, GCF_003408845.1) and mapped the reads to them using bbmap with standard parameters. The proportion of reads assigned to *Gardnerella* by kraken2 was then distributed among *Gardnerella* species in the proportion of their mapping. Viruses, including phages, were annotated with kmcp [42] against the genbank-viral database with kmcp search –try-se (to attempt single-end classification where necessary) and parsed with kmcp profile at the species level and in mode 3 (standard).

### Metagenomic assembly, binning, and annotation

Before assembly, metagenomic read libraries were normalized with bbnorm [43] to discard reads with a coverage < 3 and subsample those with a coverage > 80. All available samples for each participant were co-assembled using Spades (v.3.10.1) in metagenomic mode (metaspades.py) [44]. Reads were mapped back to contigs using bbmap [43]. Before mapping, contigs were filtered to retain those with > 1 kbp and contigs longer than 20 kbp were broken up into 10 kbp segments with a 100-bp overlap. The mapping and composition information was used for metagenomic binning using CONCOCT v1.1.0 [45]. Proteins were called and annotated using Prokka [42]. Bins were then analyzed with checkm [46] and retained if they presented > 90% completeness and < 2% contamination. Phylogenetic trees were built with FastTree with standard parameters [47] and plotted with Roary [48] using the gene_presence_absence matrix. Phylogenomic and pangenomic analyses were run in Panaroo [49] with standard settings, albeit gene enrichment analysis was run with Scoary [50]. Since Scoary only accepts dichotomous variables, we classified both “constantly eubiotic" and “menses-related dysbiotic” as “mid-cycle eubiosis”, in contrast to the “constant dysbiosis” and “unstable” groups.

### Statistics and figures

All figures were generated in R v4.2.2. Alpha- and Beta-diversity statistics were calculated with Vegan v.2.6.4 Alpha-diversity was calculated as inverse Simpson’s, and beta-diversity as Aitchinson’s distance unless specified as Jaccard’s. Differences in prevalence were calculated as chi-square tests, considering a minimal of 0.5% of reads as “presence” and corrected for multiple testing with the Benjamini–Hochberg procedure (BH). Associations of specific bacteria to CST and/or VCD were calculated in ANCOM-BC v2.0.1, treating participant ID as a random factor, and corrected by BH [51]. CSTs were assigned with VALENCIA, github commit c41897d [22]. Time series were further classified into four VCDs (constantly eubiotic, menses-related dysbiotic, unstable, and constantly dysbiotic) as described in the main text and in www.github.com/ctmrbio/valody. Differences in total phage content between groups were calculated with Kruskal–Wallis tests. Since groups were too small to allow treating participant ID as a random effect, we minimized the effect of specific participants by subsampling each participant to 10 (all samples combined) or 5 (each VCD) samples/participant, 10 times. If *p* < 0.05 in at least 7 of the 10 trials, the test was considered significant also when adjusting for random effects. For these, a post-hoc Dunn’s test with BH correction was used to pinpoint differences between VCD.

### Supplementary Information

**Additional file 1:** Supplementary Figure S1. Bacterial and viral profiles for each sample over one menstrual cycle. Each participant’s bacterial and viral profile are depicted as area plots. Sexual intercourse is overlaid as blue dots and vaginal bleedings as red dots. Log10 of the ratio of viral to bacterial reads is shown as a black line over the viral profiles, for time-series with sufficient data (> 5 samples with detectable phages). Missing data is omitted. Next to each taxonomic profile is an ordination showing all samples in the study as gray circles, and the samples for the relevant participant as numbers, following the days of their menstrual cycle. Days with vaginal bleedings are shown in red, days with sexual intercourse in blue and days with both events in purple. Supplementary Figure S2. CST distribution and time-series dynamics for the 16S samples. CSTs are shown as colored dots as per the legend in the second part. The outline of each box depicts the assignment to vaginal community dynamics. Missing samples are omitted. Bleedings are marked as red dots. Blue: constant eubiotic. Green: menses-related dysbiotic. Yellow: unstable. Red: constant dysbiotic. Supplementary Figure S3. CST distribution and time-series dynamics for the shotgun samples. CSTs are marked as colored dots above the taxonomic profiles as per the legend. The outline of each box depicts its dynamic group. Bleedings are marked as light red dots. Missing samples are omitted. Blue: constant eubiotic. Green: menses-related dysbiotic. Yellow: Unstable. Red: constant dysbiotic. Supplementary Figure S4. Log-fold change of bacterial species in samples from CST-I. Samples in CST-IA and CST-IB from menses-related dysbiotic or unstable individuals were compared to constant eubiotic individuals. The heatmap shows the log-fold change of all significant differences. Gray fields represent no significant change. Supplementary Figure S5. Log-fold change of bacterial species in samples from CST-III. Samples in CST-IIIA and CST-IIIB from menses-related dysbiotic or unstable individuals were compared with constant dysbiotic individuals. The heatmap shows the log-fold change of all significant differences. White fields represent no significant change. Supplementary Figure S6. Volcano plots for the vaginal community dynamics compared to either constant eubiotic or constant dysbiotic. Supplementary Figure S7. Histograms showing gene cluster prevalence in nine relevant pangenomes. For each species, the prevalence (number of genomes containing each gene cluster) of each gene cluster is shown as a histogram. Gene clusters present in most or all genomes are considered “core”, while those in one or very few genomes can be considered “cloud”. The “shell” genomes, present in many, but not all genomes, are less frequent in this dataset. Supplementary Figure S8. Phylogenomic analysis of *Lactobacillus* genomes. Phylogenomic analysis of all detected *Lactobacillus* species does not find a correlation between the womens’ vaginal community dynamics and the observed phylogeny. The presence of a gene is represented in dark blue and its absence in light blue. Blue: constant eubiotic. Red: constant dysbiotic. Yellow: unstable. Green: menses-related dysbiotic. Supplementary Figure S9. Phylogenomic analysis of *Prevotella* genomes. Phylogenomic analysis of all detected *Prevotella* species does not find a correlation between the womens’ vaginal community dynamics and the observed phylogeny. The presence of a gene is represented in dark blue and its absence in light blue. Blue: constant eubiotic. Red: constant dysbiotic. Yellow: unstable. Green: menses-related dysbiotic.

**Additional file 2:** Supplementary Table S1. Full ASV table for samples sequenced by 16S marker gene sequencing. Each sample is in a column, named by individual ID and cycle day, and each ASV in a row. Taxonomic annotations are in the second-to-last column and centroid sequence in the last. Supplementary Table S2. Full taxonomic annotation and feature counts for the samples sequenced by shotgun. Each sample is in a column, named by participant and cycle day, and each taxon in a row. Supplementary Table S3. Differential abundance results for samples in CST-IA from individuals with menses-related dysbiotic or unstable VCD compared to constant eubiotic VCD. Supplementary Table S4. Differential abundance results for samples in CST-IB from individuals with menses-related dysbiotic or unstable VCD compared to constant eubiotic VCD. Supplementary Table S5. Differential abundance results for samples in CST-IIIA from individuals with menses-related dysbiotic or unstable VCD compared to constant dysbiotic VCD. Supplementary Table S6. Differential abundance results for samples in CST-IIIB from individuals with menses-related dysbiotic or unstable VCD compared to constant dysbiotic VCD. Supplementary Table S7. Differential abundance results for all samples in menses-related dysbiotic, unstable and constant dysbiotic VCD against constant eubiotic. Supplementary Table S8. Differential abundance results for all samples in menses-related dysbiotic, unstable and constant eubiotic VCD against constant dysbiotic. Supplementary Table S9. Differential frequency of gene clusters in *Lactobacillus* spp.*,* contrasting constant eubiotic and menses-related dysbiotic vs. unstable and constant dysbiotic. Supplementary Table S10. Differential frequency of gene clusters in *Gardnerella* spp.*,* contrasting constant eubiotic and menses-related dysbiotic vs. unstable and constant dysbiotic. Supplementary Table S11. Differential frequency of gene clusters in *Prevotella* spp.*,* contrasting constant eubiotic and menses-related eubiotic vs. unstable and constant dysbiotic.

## Data Availability

All sequencing data has been submitted to the European Nucleotide Archive under project PRJEB37731. The 16S samples have accession ID ERS14866734-ERS14867253, and the shotgun samples, after human DNA removal, have ID ERS14864440-ERS14865713. The code is available at https://github.com/ctmrbio/valody/commit/17cb300a4571819260daa54319473f8a5dc9161a.
