# Supplementary material for: Defining Vaginal Community Dynamics: daily microbiome transitions, the role of menstruation, bacteriophages, and bacterial genes
Source: Microbiome. 2024 Aug 19;12:153. doi: 10.1186/s40168-024-01870-5 (PMC11331738; doi:10.1186/s40168-024-01870-5)

Table of contents

Figure S1……………………………………………………………………………..p. 2-28

Figure S2……………………………………………………………………………..p. 29-31

Figure S3……………………………………………………………………………..p. 32-38

Figure S4……………………………………………………………………………..p. 39-40

Figure S5……………………………………………………………………………..p. 41-42

Figure S6……………………………………………………………………………..p. 43

Figure S7……………………………………………………………………………..p. 44

Figure S8……………………………………………………………………………..p. 45

Figure S9……………………………………………………………………………..p. 46

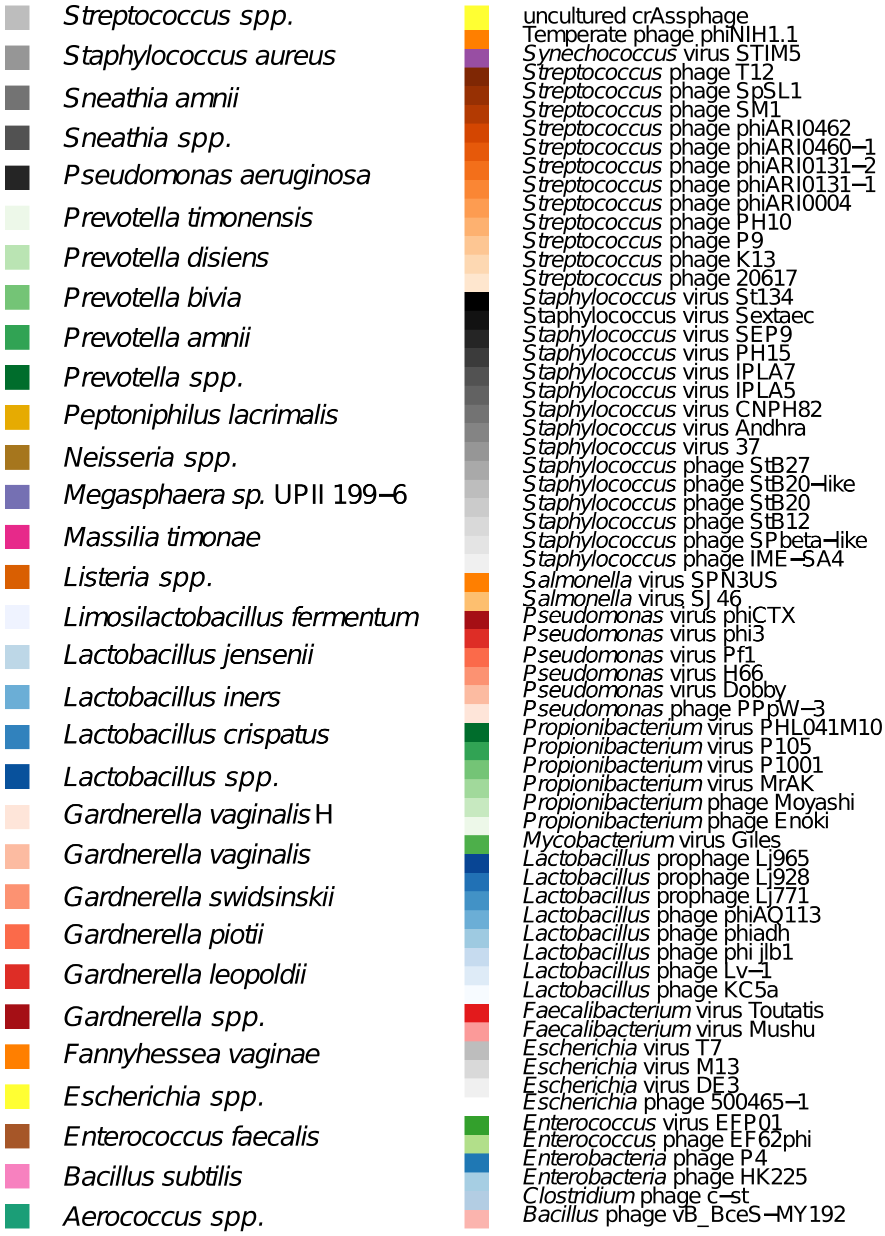

Streptococcus spp.
Staphylococcus aureus
Sneathia amnii
Sneathia spp.
Pseudomonas aeruginosa
Prevotella timonensis
Prevotella disiens
Prevotella bivia
Prevotella amnii
Prevotella spp.
Peptoniphilus lacrimalis
Neisseria spp.
Megasphaera sp. UPII 199−6
Massilia timonae
Listeria spp.
Limosilactobacillus fermentum
Lactobacillus jensenii
Lactobacillus iners
Lactobacillus crispatus
Lactobacillus spp.
Gardnerella vaginalis H
Gardnerella vaginalis
Gardnerella swidsinskii
Gardnerella piotii
Gardnerella leopoldii
Gardnerella spp.
Fannyhessea vaginae
Escherichia spp.
Enterococcus faecalis
Bacillus subtilis
Aerococcus spp.
uncultured crAssphage
Temperate phage phiNIH1.1
Synechococcus virus STIM5
Streptococcus phage T12
Streptococcus phage SpSL1
Streptococcus phage SM1
Streptococcus phage phiARI0462
Streptococcus phage phiARI0460−1
Streptococcus phage phiARI0131−2
Streptococcus phage phiARI0131−1
Streptococcus phage phiARI0004
Streptococcus phage PH10
Streptococcus phage P9
Streptococcus phage K13
Streptococcus phage 20617
Staphylococcus virus St134
Staphylococcus virus Sextaec
Staphylococcus virus SEP9
Staphylococcus virus PH15
Staphylococcus virus IPLA7
Staphylococcus virus IPLA5
Staphylococcus virus CNPH82
Staphylococcus virus Andhra
Staphylococcus virus 37
Staphylococcus phage StB27
Staphylococcus phage StB20−like
Staphylococcus phage StB20
Staphylococcus phage StB12
Staphylococcus phage SPbeta−like
Staphylococcus phage IME−SA4
Salmonella virus SPN3US
Salmonella virus SJ46
Pseudomonas virus phiCTX
Pseudomonas virus phi3
Pseudomonas virus Pf1
Pseudomonas virus H66
Pseudomonas virus Dobby
Pseudomonas phage PPpW−3
Propionibacterium virus PHL041M10
Propionibacterium virus P105
Propionibacterium virus P1001
Propionibacterium virus MrAK
Propionibacterium phage Moyashi
Propionibacterium phage Enoki
Mycobacterium virus Giles
Lactobacillus prophage Lj965
Lactobacillus prophage Lj928
Lactobacillus prophage Lj771
Lactobacillus phage phiAQ113
Lactobacillus phage phiadh
Lactobacillus phage phi jlb1
Lactobacillus phage Lv−1
Lactobacillus phage KC5a
Faecalibacterium virus Toutatis
Faecalibacterium virus Mushu
Escherichia virus T7
Escherichia virus M13
Escherichia virus DE3
Escherichia phage 500465−1
Enterococcus virus EFP01
Enterococcus phage EF62phi
Enterobacteria phage P4
Enterobacteria phage HK225
Clostridium phage c−st
Bacillus phage vB_BceS−MY192

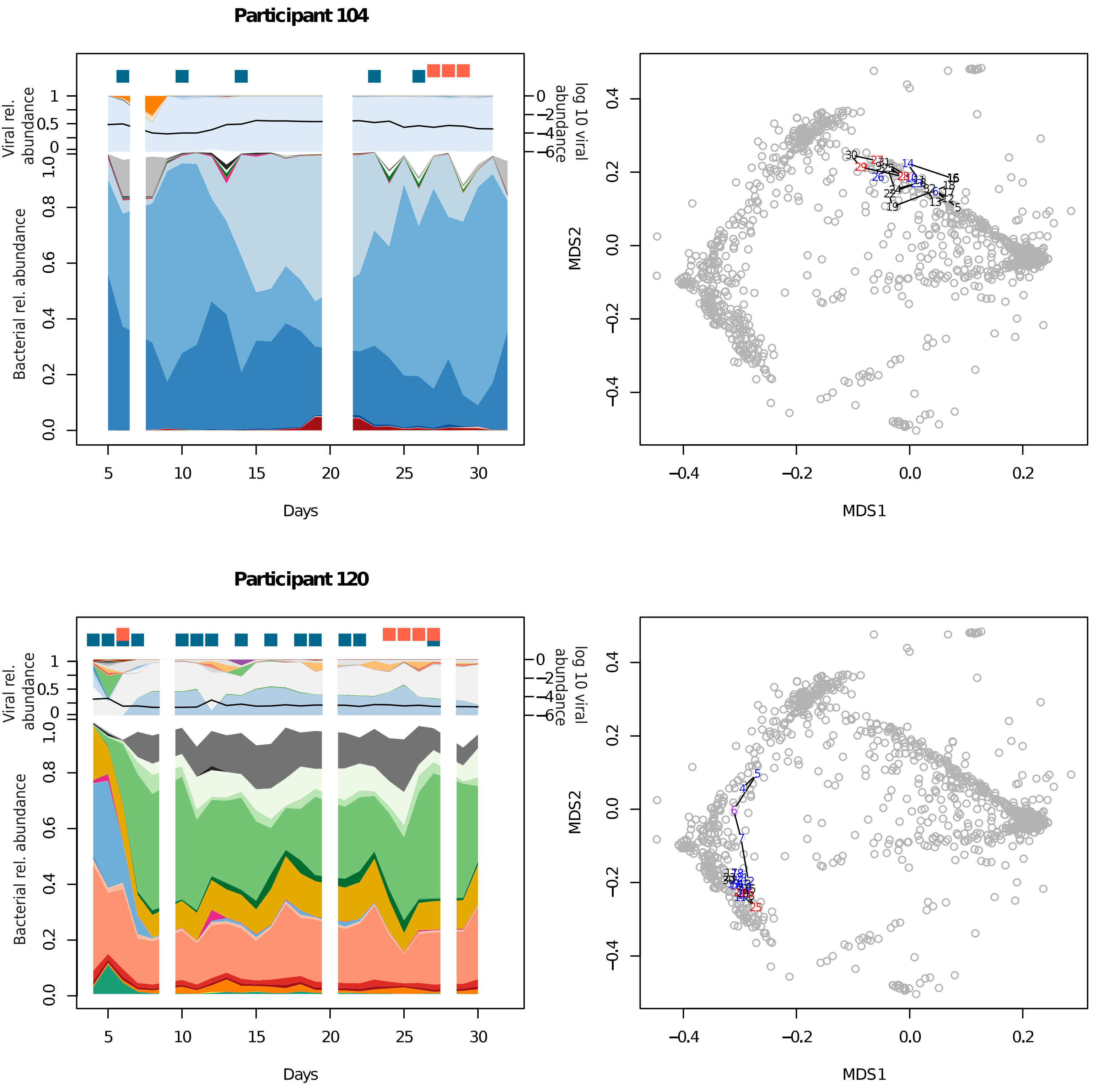
Participant 104
Viral rel. abundance
Bacterial rel. abundance
log 10 viral abundance
Days
MDS2
MDS1
Participant 120
Viral rel. abundance
Bacterial rel. abundance
log 10 viral abundance
Days
MDS2
MDS1

Participant 141

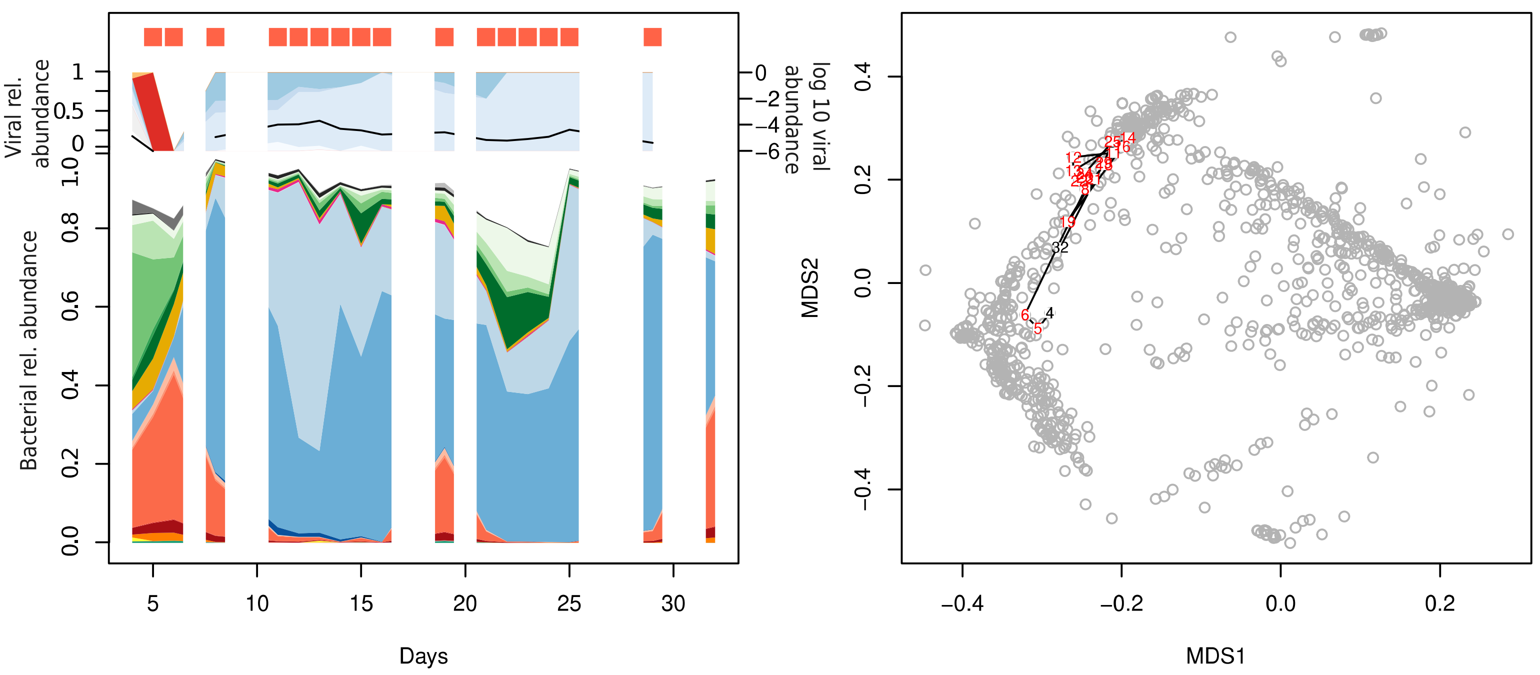

Participant 156

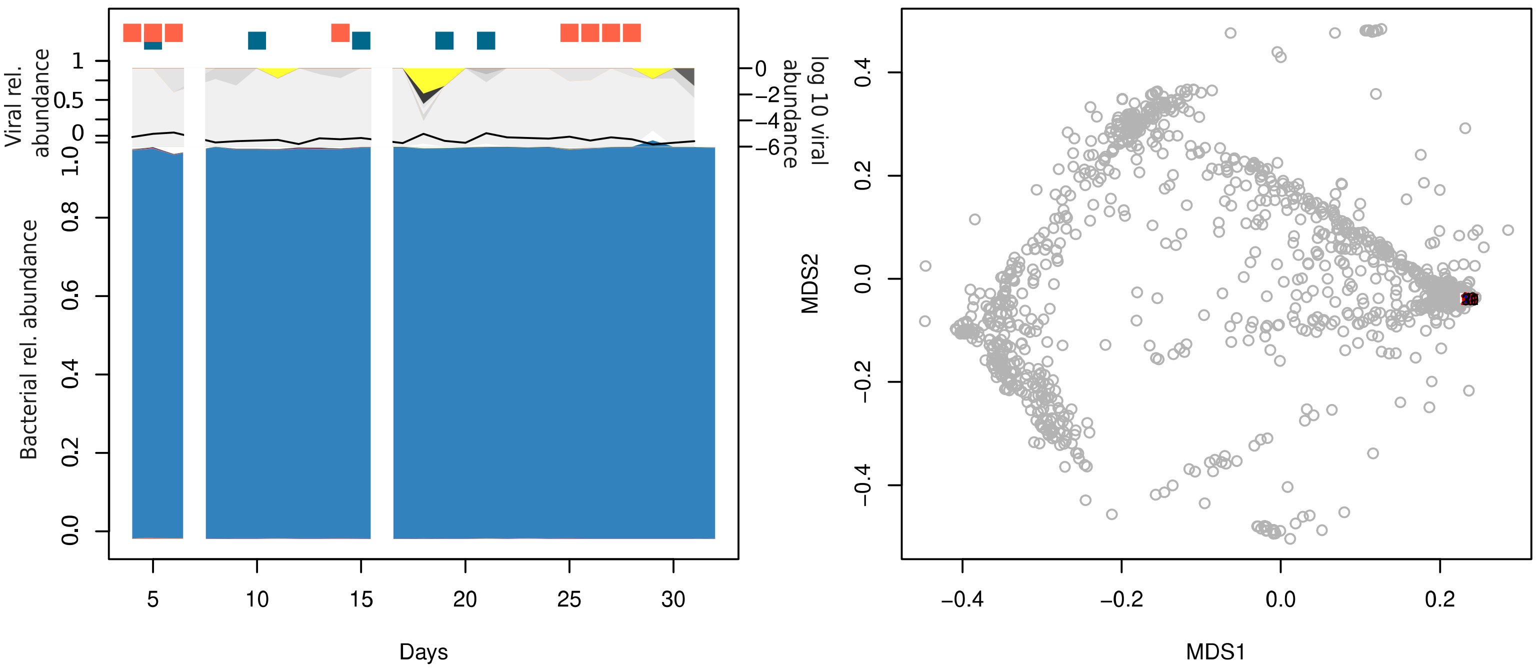

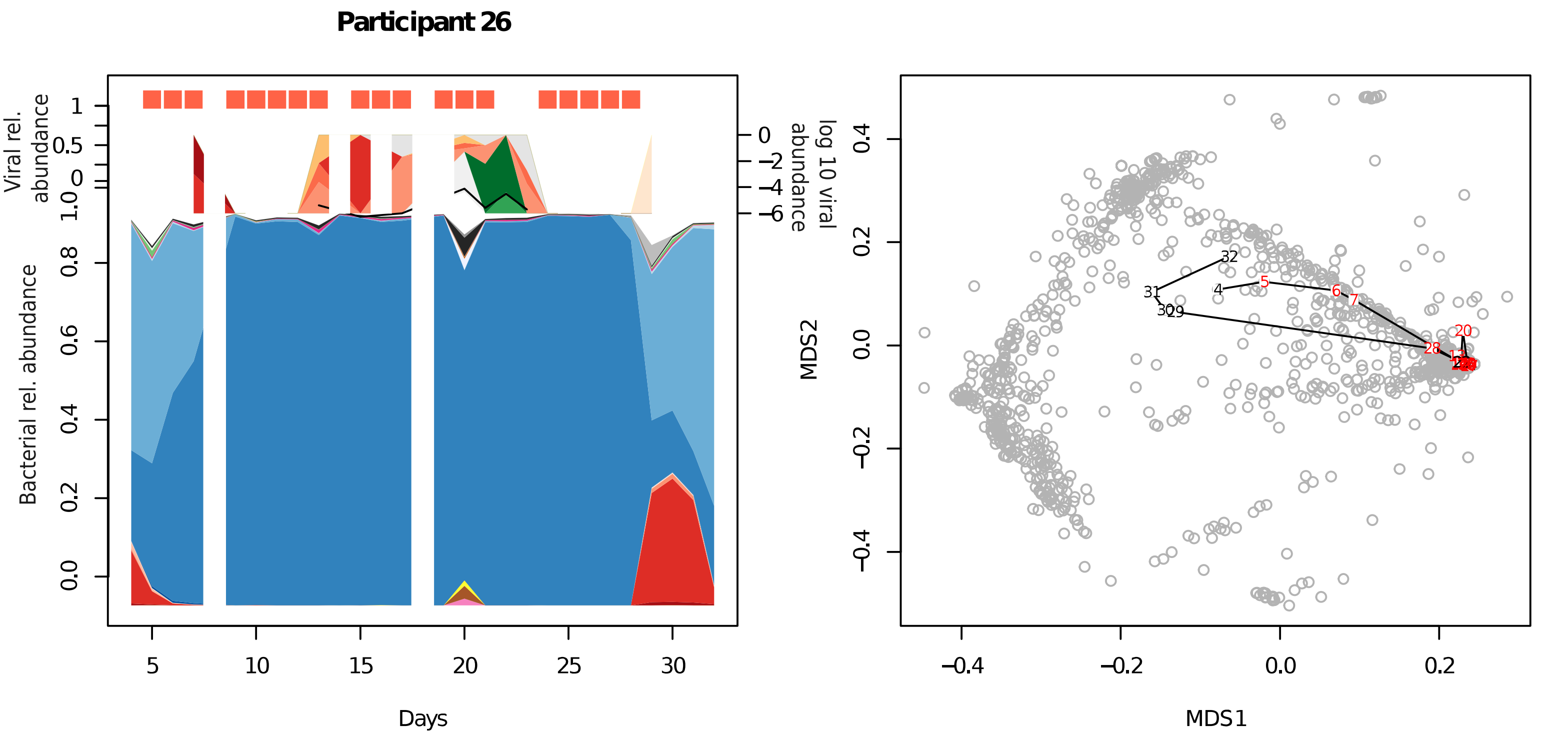

Participant 26
Viral rel. abundance
Bacterial rel. abundance
log 10 viral abundance
Days
MDS1
MDS2

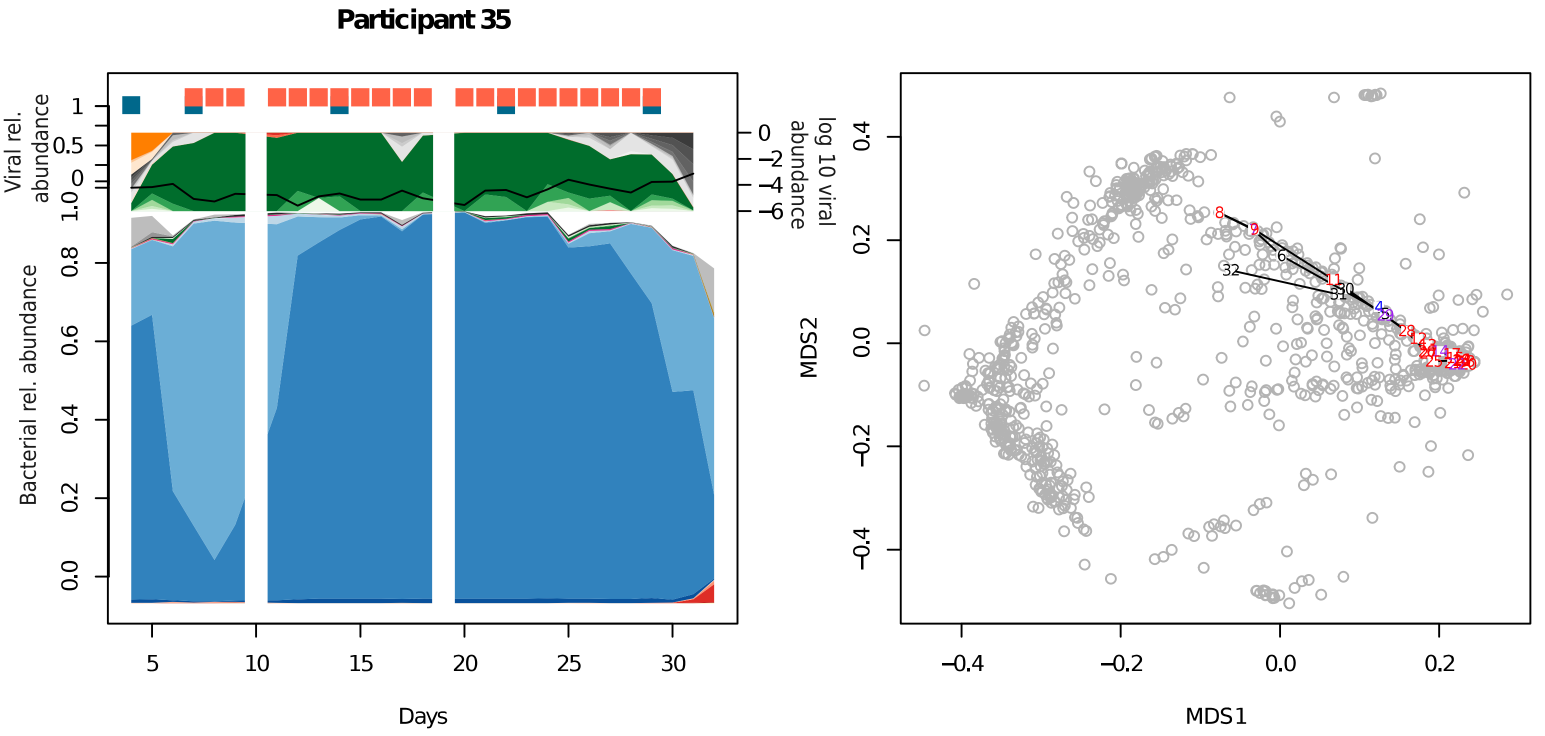

Participant 35
Viral rel. abundance
Bacterial rel. abundance
log 10 viral abundance
Days
MDS1
MDS2

Participant 58

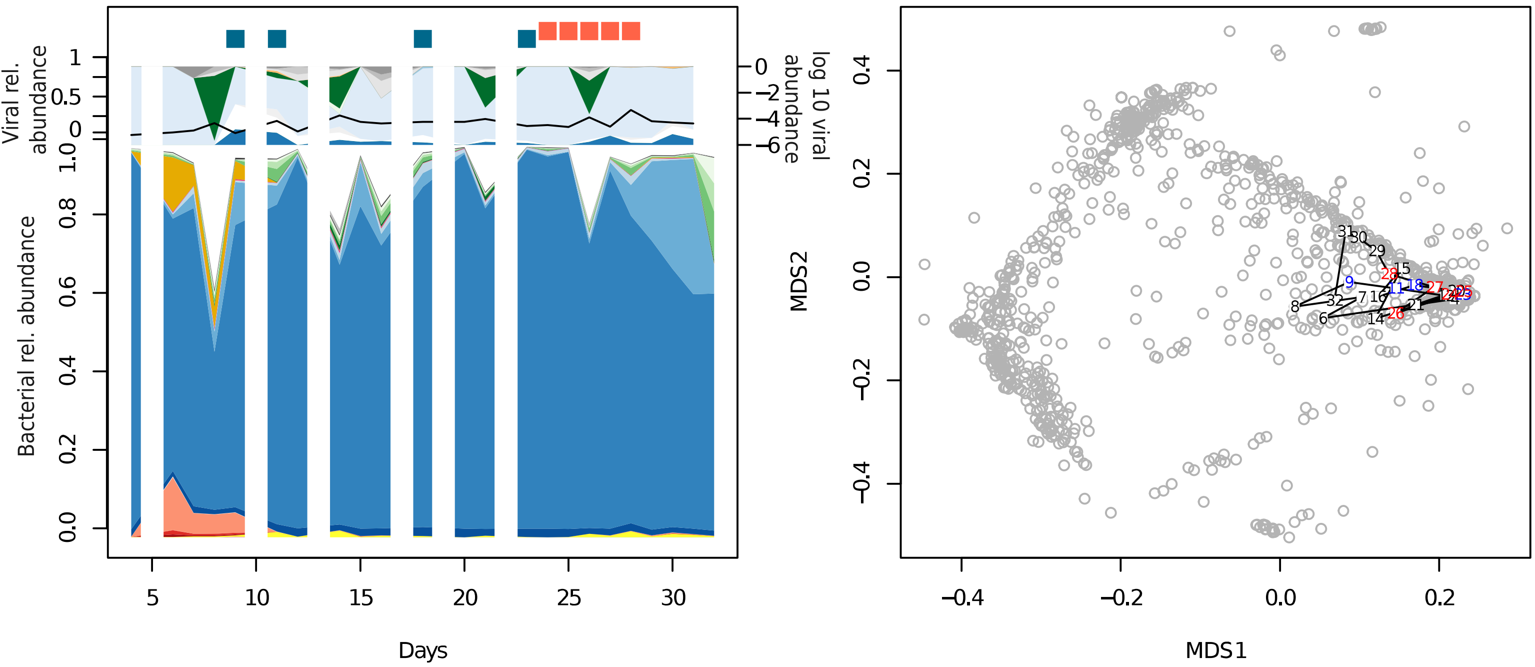

Participant 81

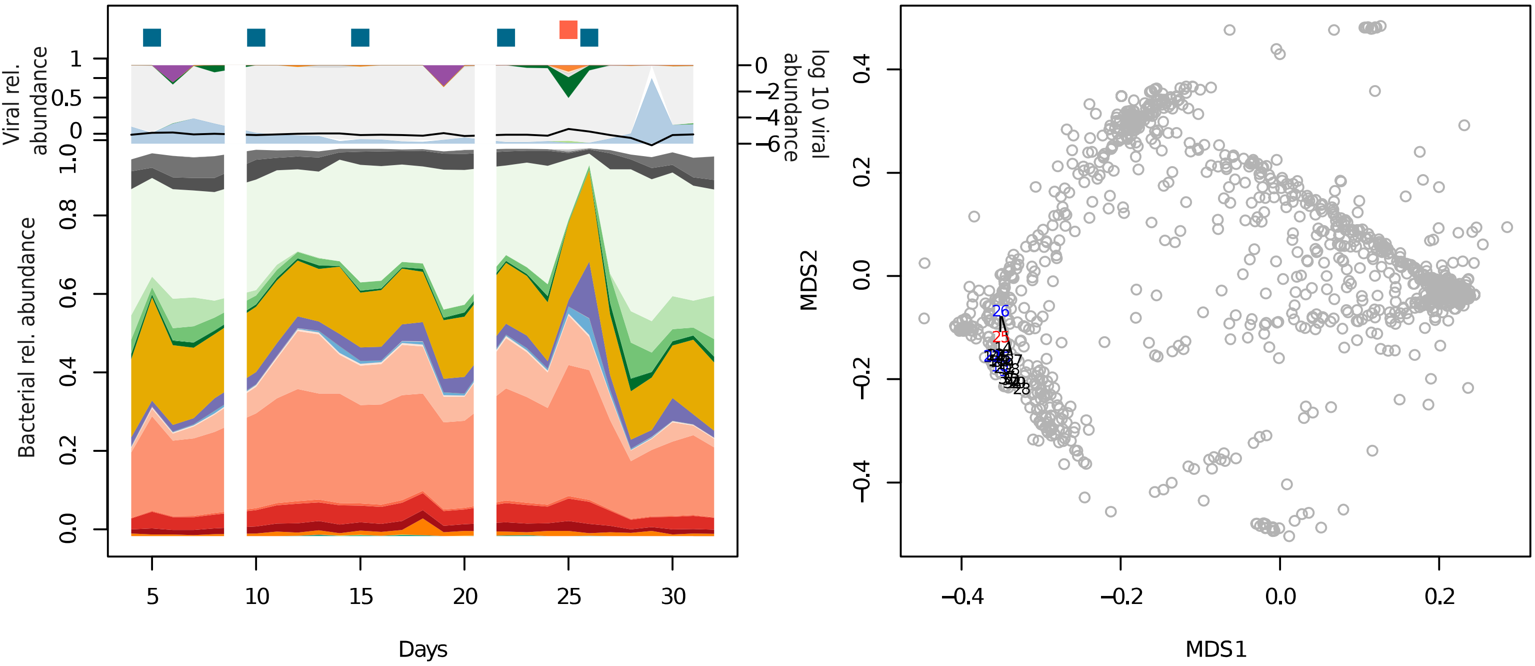

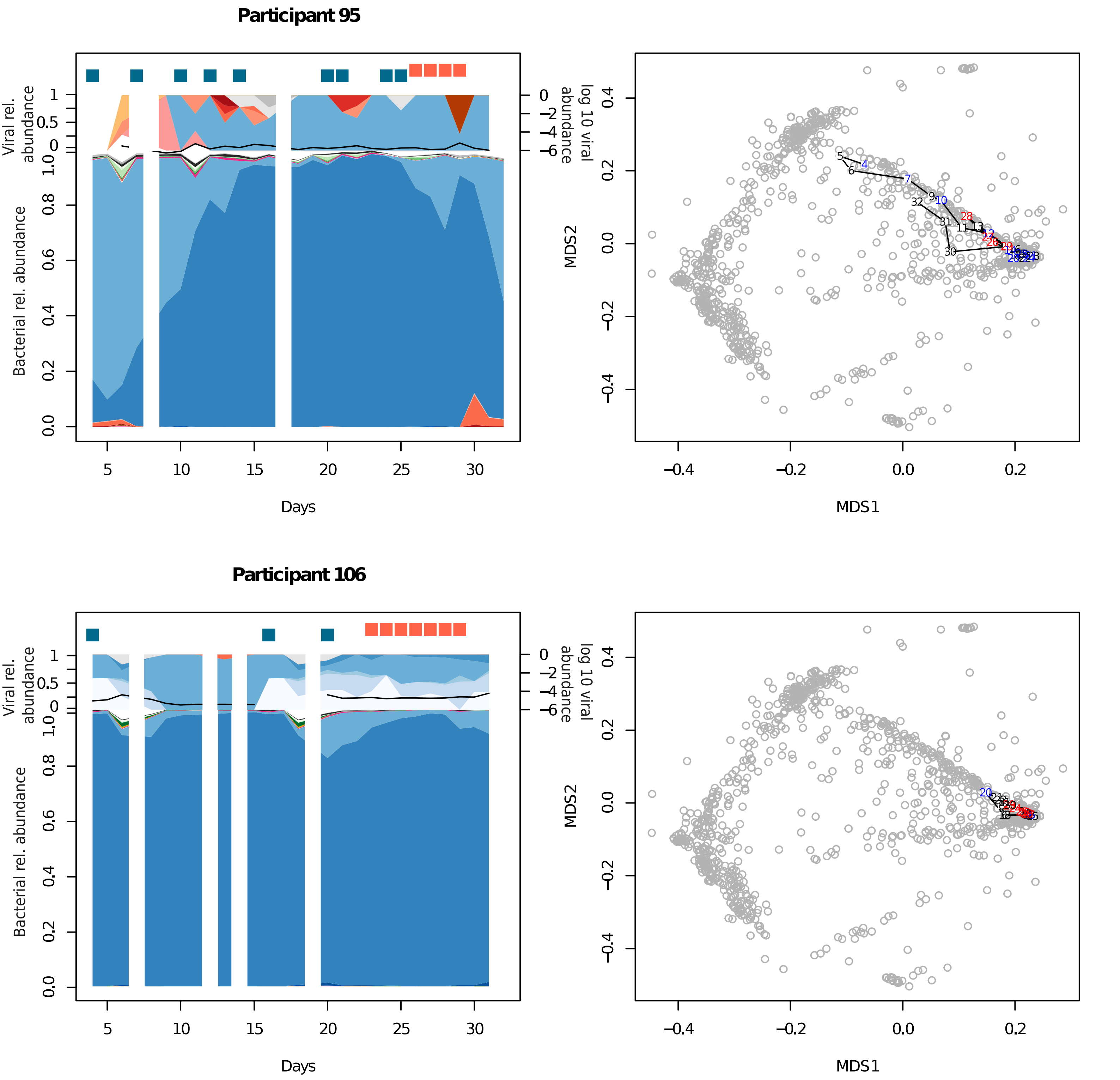
Participant 95
Viral rel. abundance
Bacterial rel. abundance
log 10 viral abundance
Days
MDS1
MDS2
Participant 106
Viral rel. abundance
Bacterial rel. abundance
log 10 viral abundance
Days
MDS1
MDS2

Participant 124

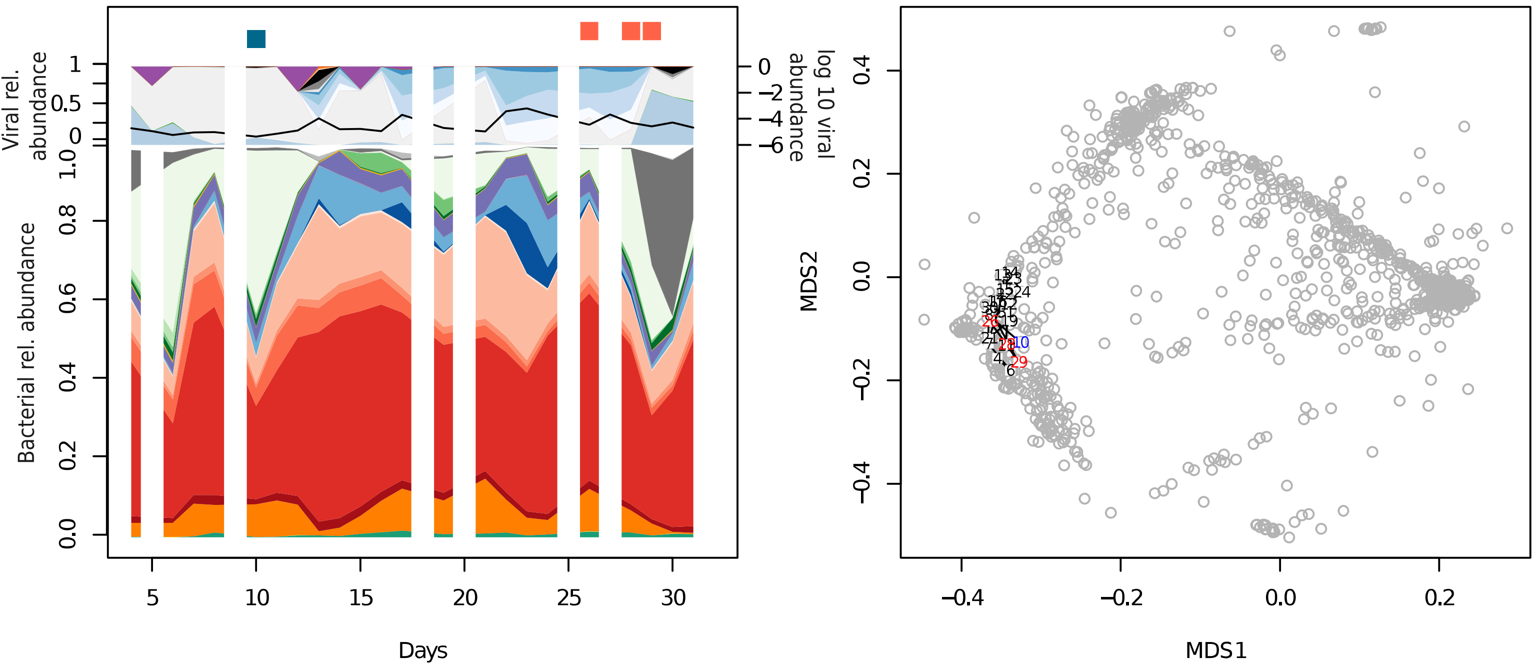

Participant 144

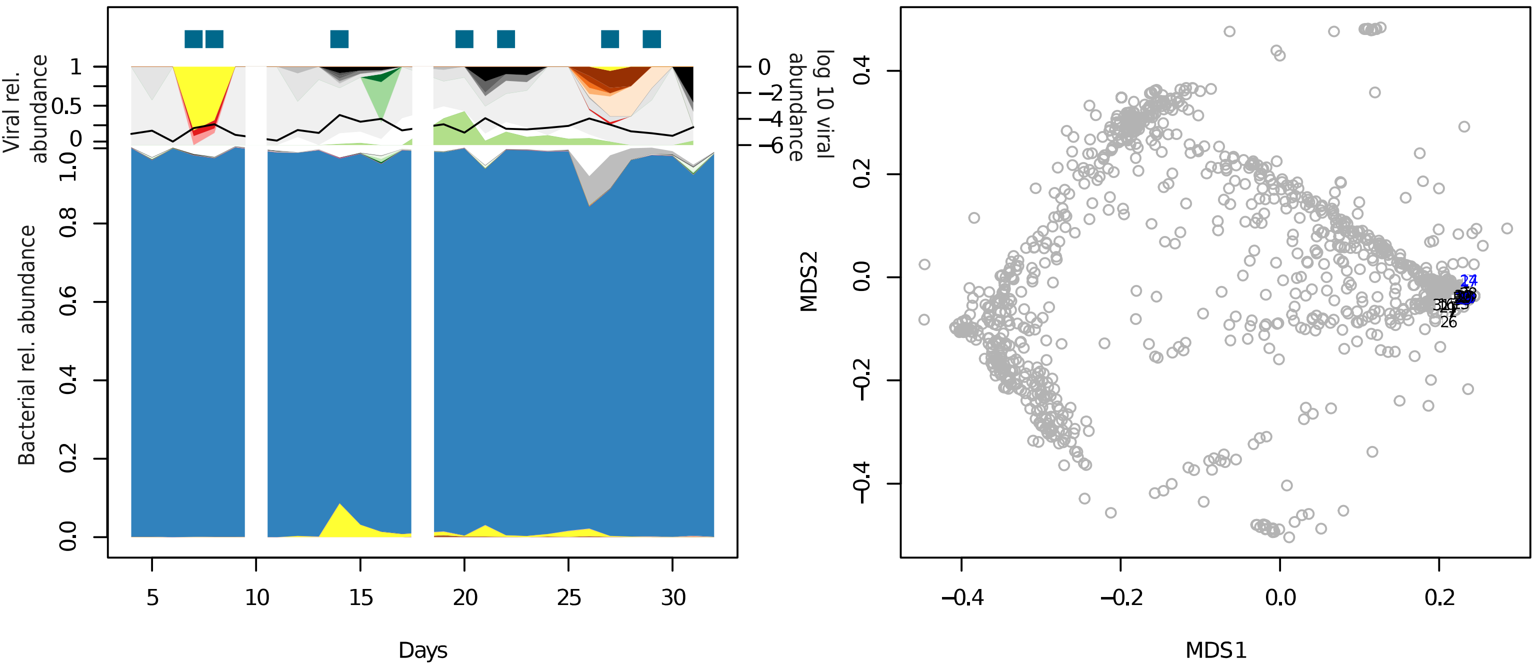

Participant 28

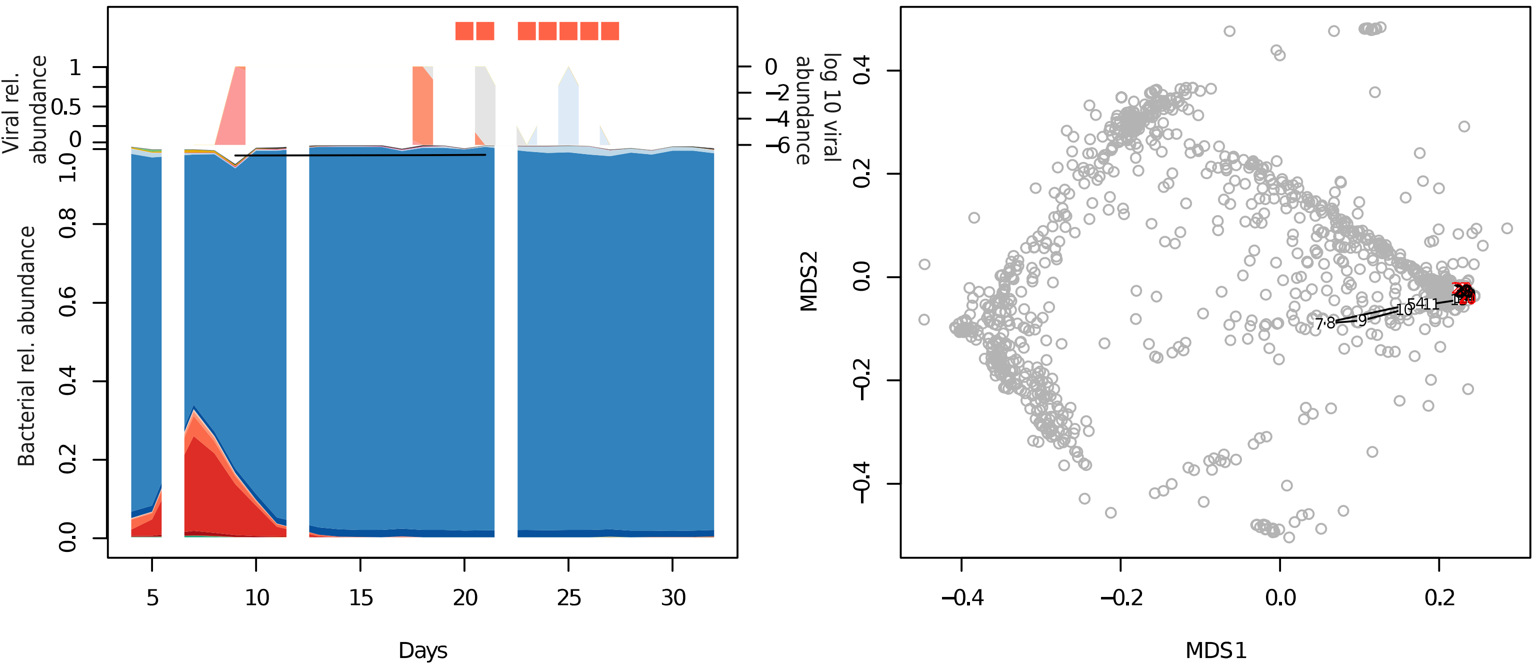

Participant 45

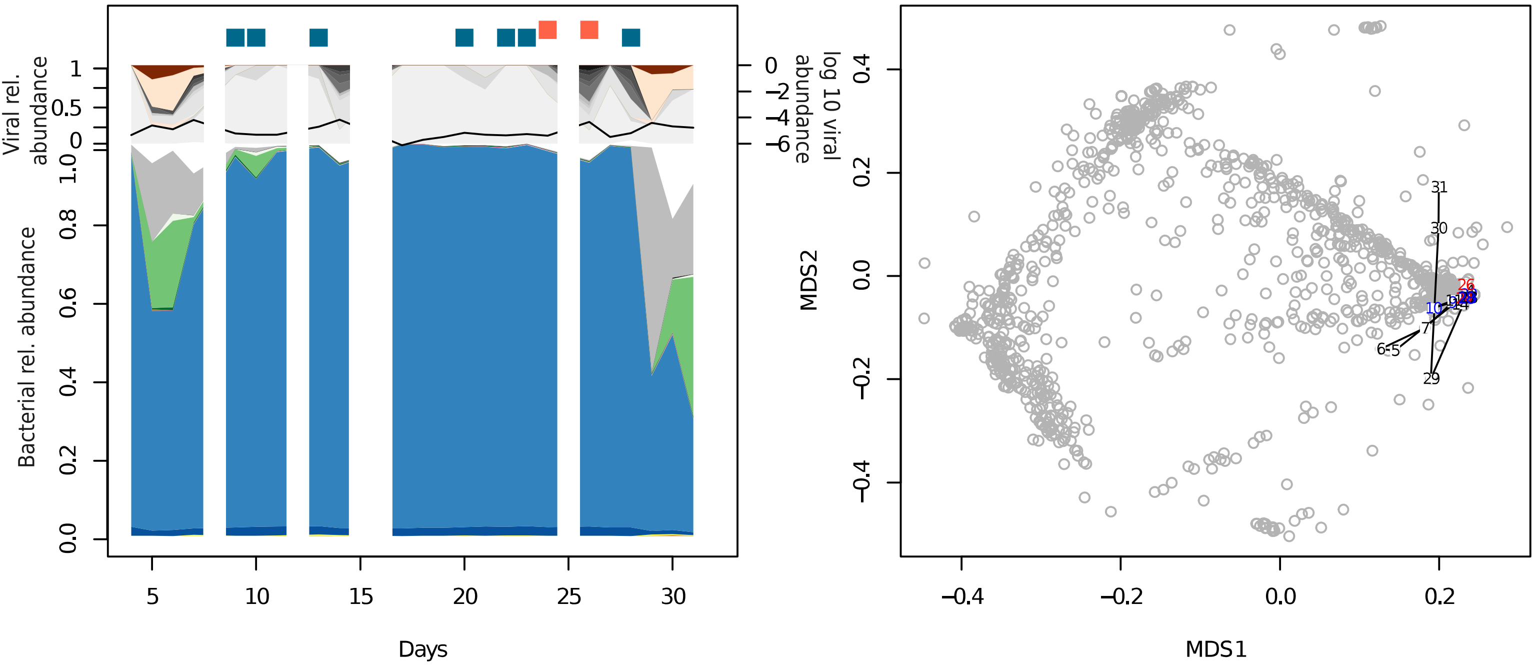

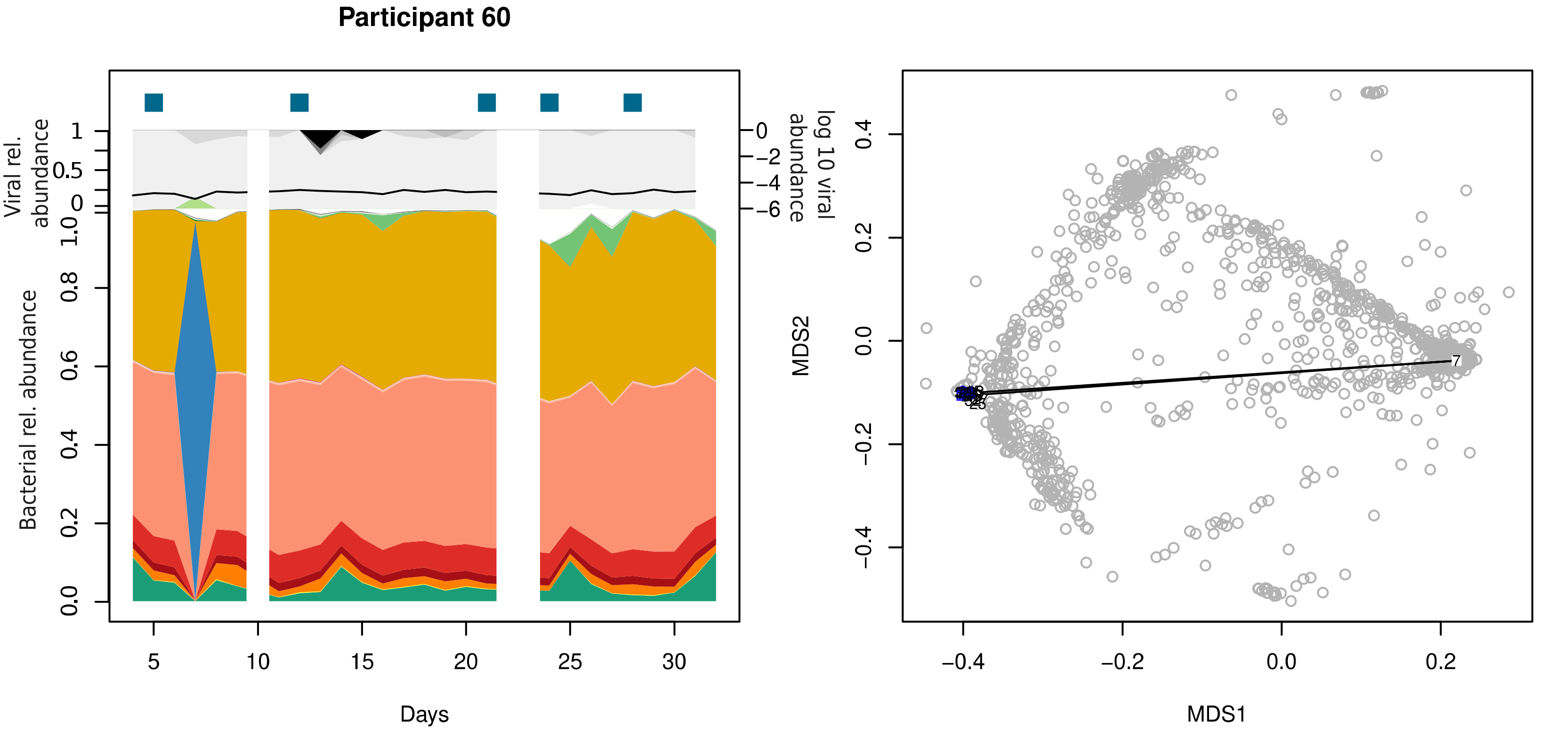
Participant 60
Viral rel. abundance
Bacterial rel. abundance
log 10 viral abundance
Days
MDS2
MDS1

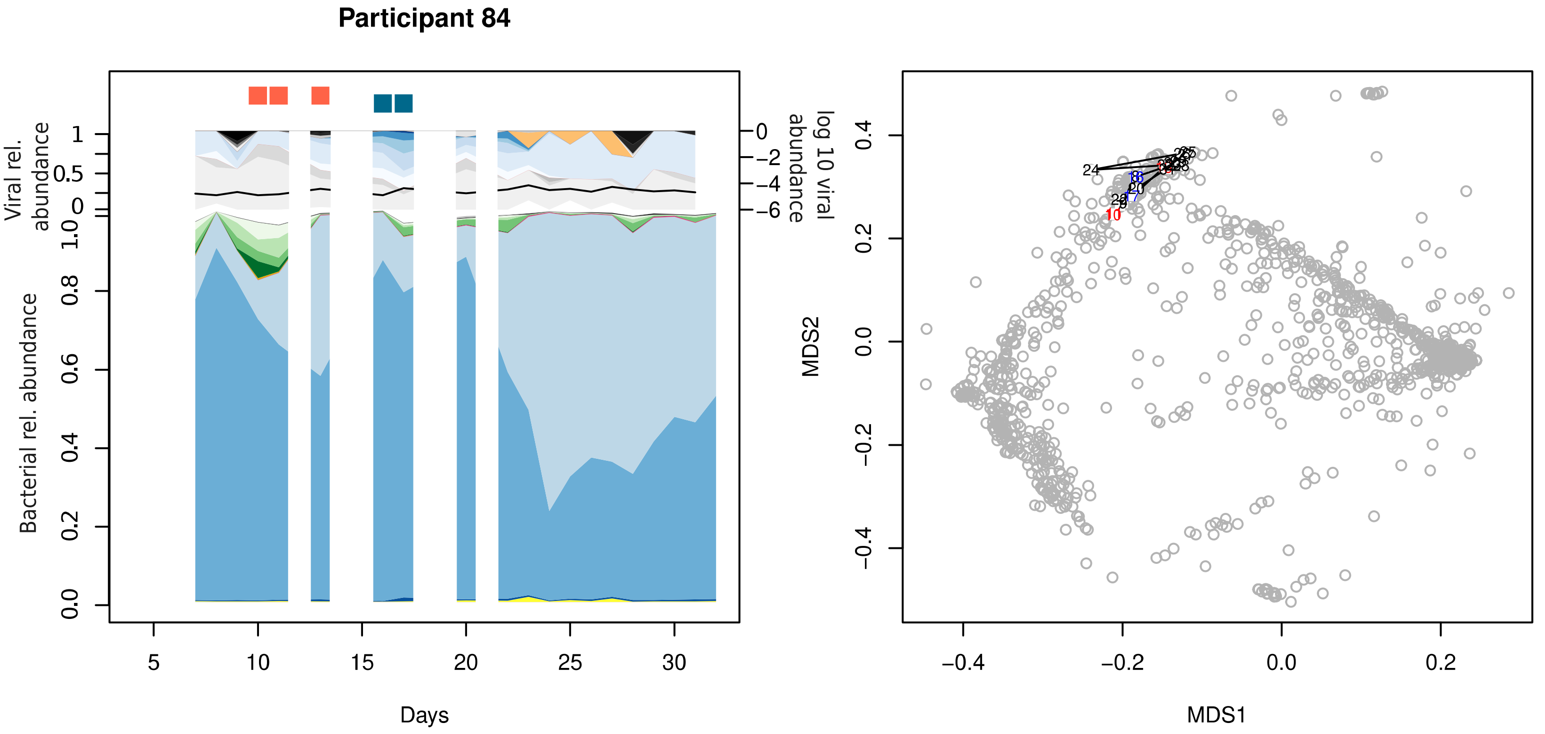
Participant 84
Viral rel. abundance
Bacterial rel. abundance
log 10 viral abundance
Days
MDS2
MDS1

Participant 97

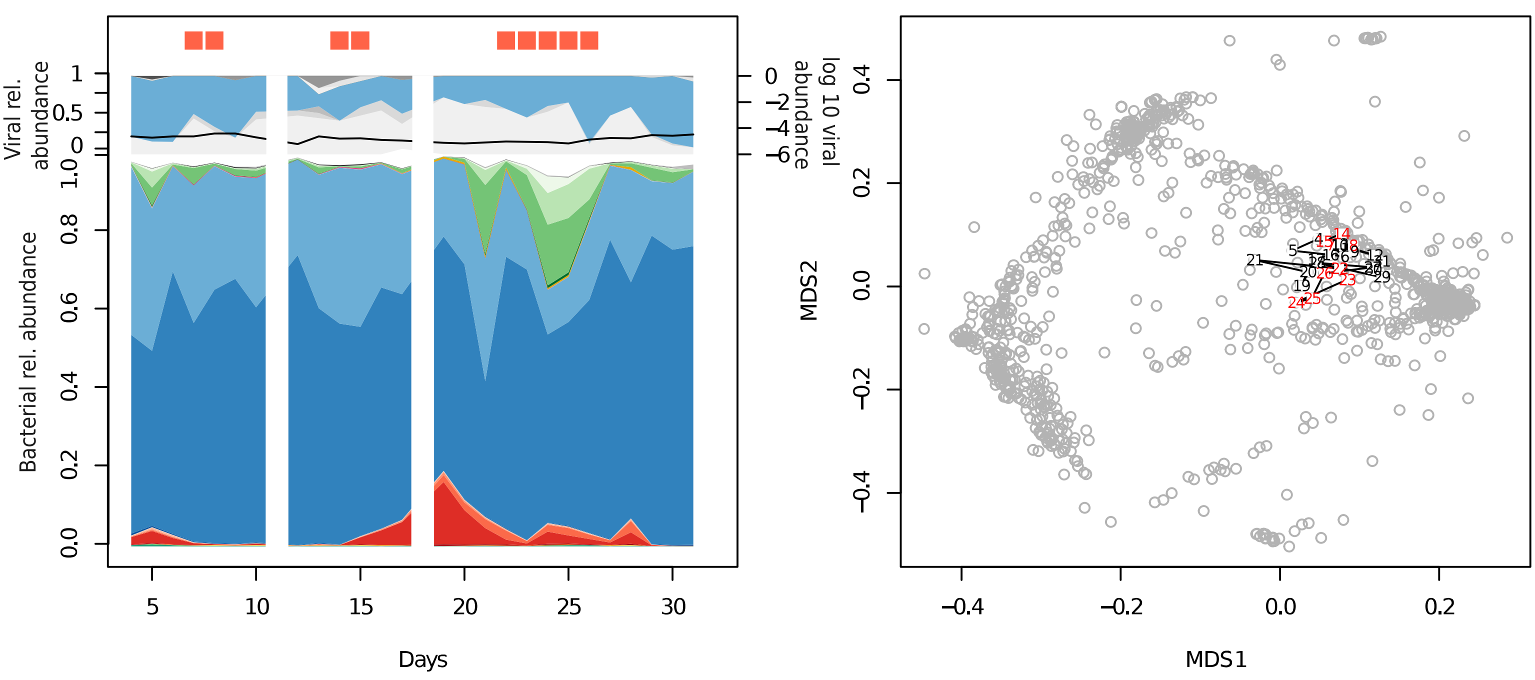

Participant 110

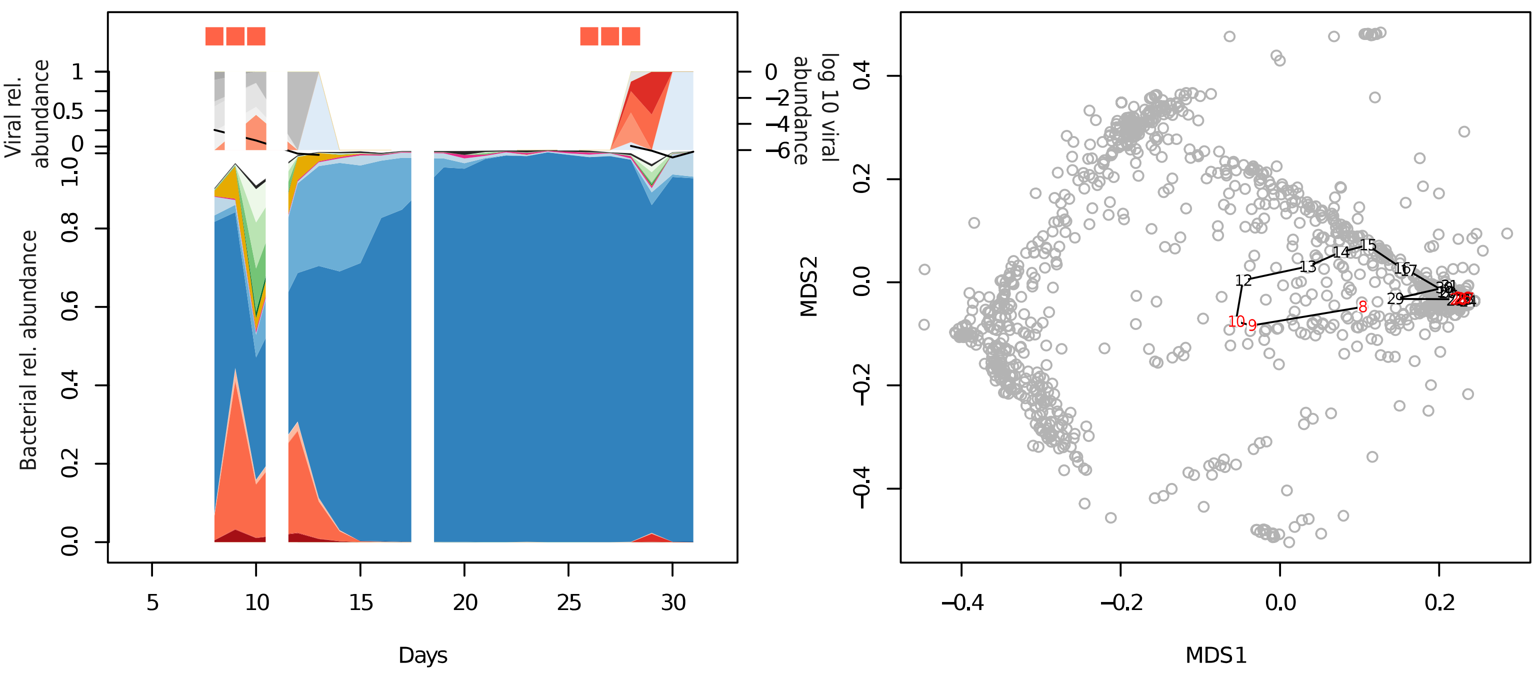

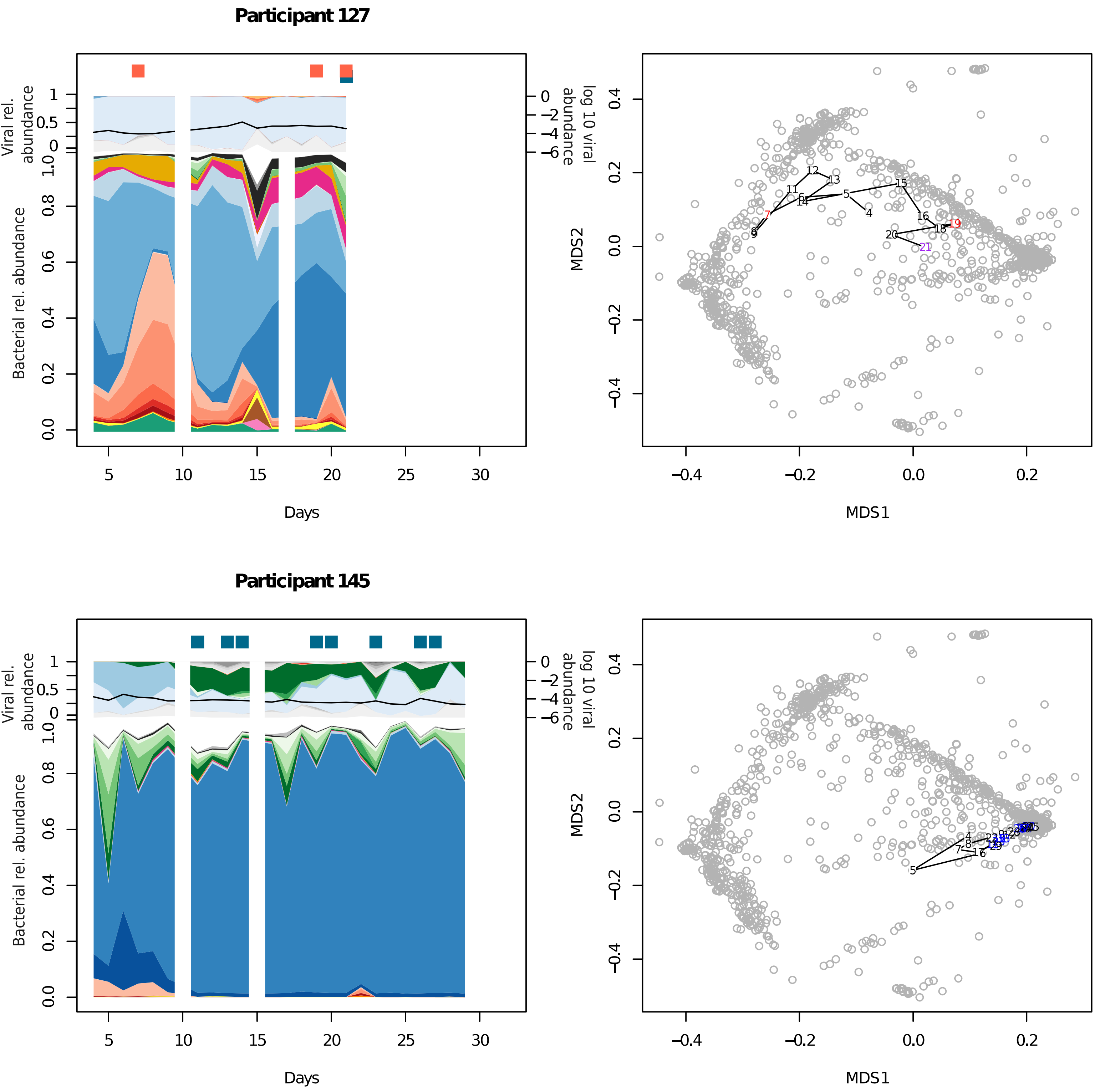
Participant 127
Viral rel. abundance
Bacterial rel. abundance
log 10 viral abundance
Days
MDS1
MDS2
Participant 145
Viral rel. abundance
Bacterial rel. abundance
log 10 viral abundance
Days
MDS1
MDS2

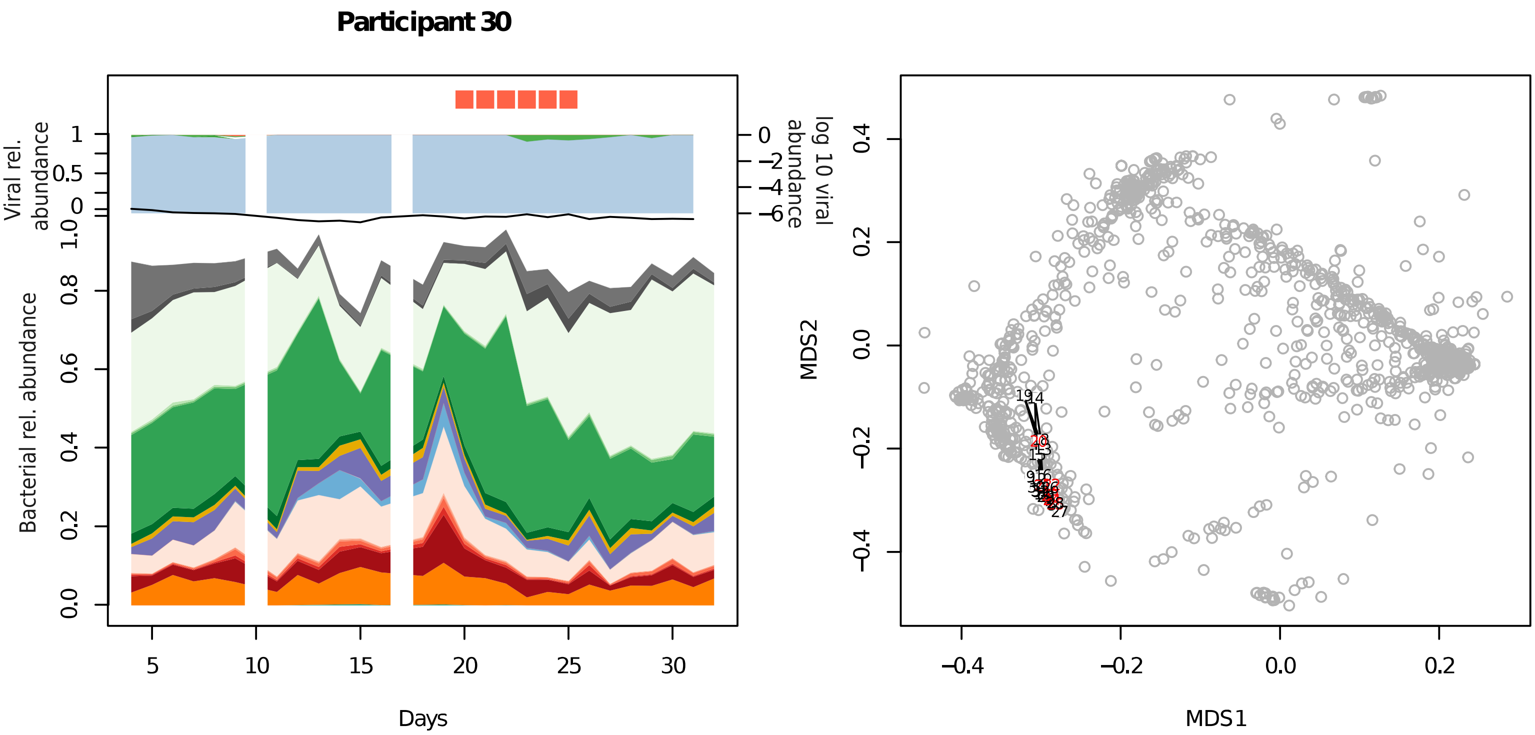
Participant 30
Viral rel. abundance
Bacterial rel. abundance
log 10 viral abundance
Days
MDS1
MDS2

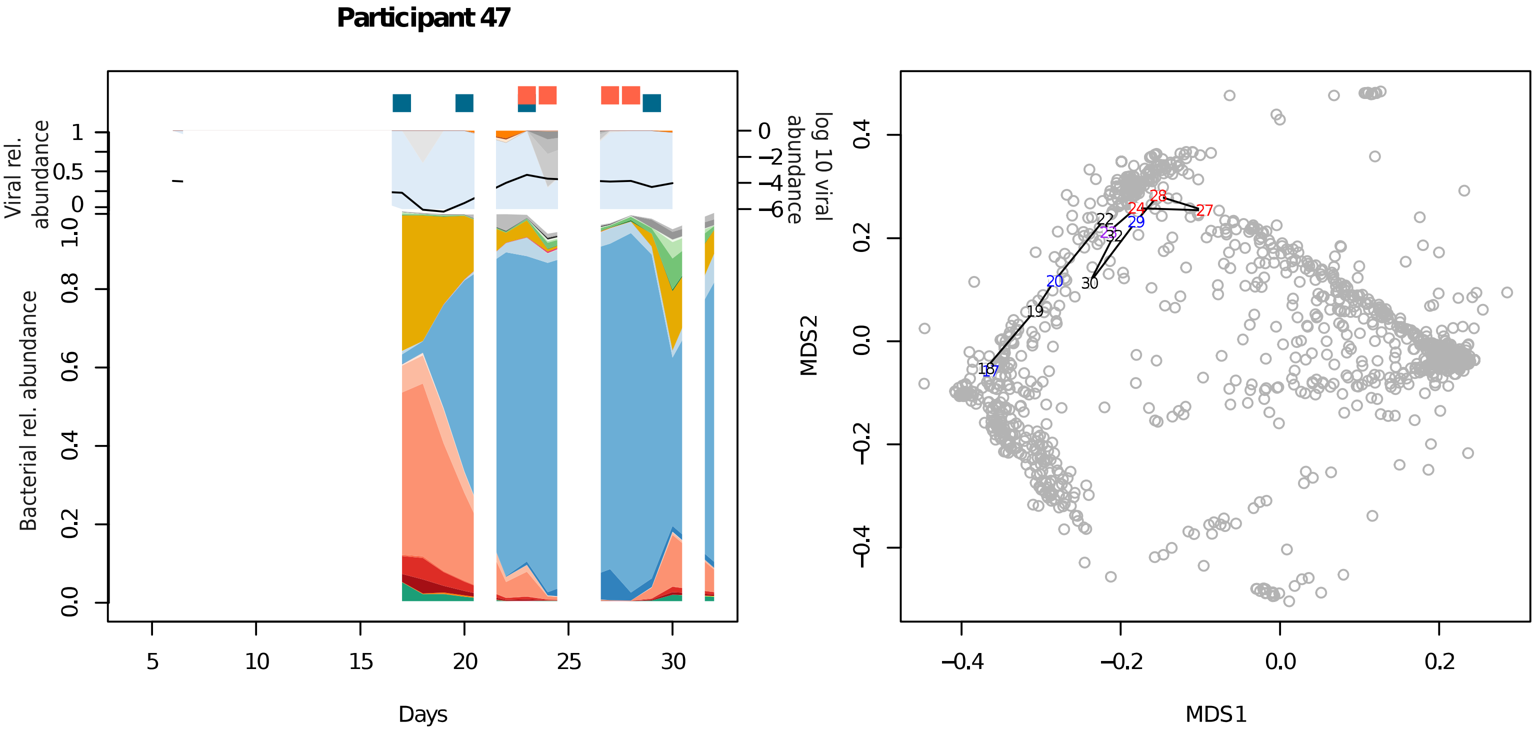
Participant 47
Viral rel. abundance
Bacterial rel. abundance
log 10 viral abundance
Days
MDS1
MDS2

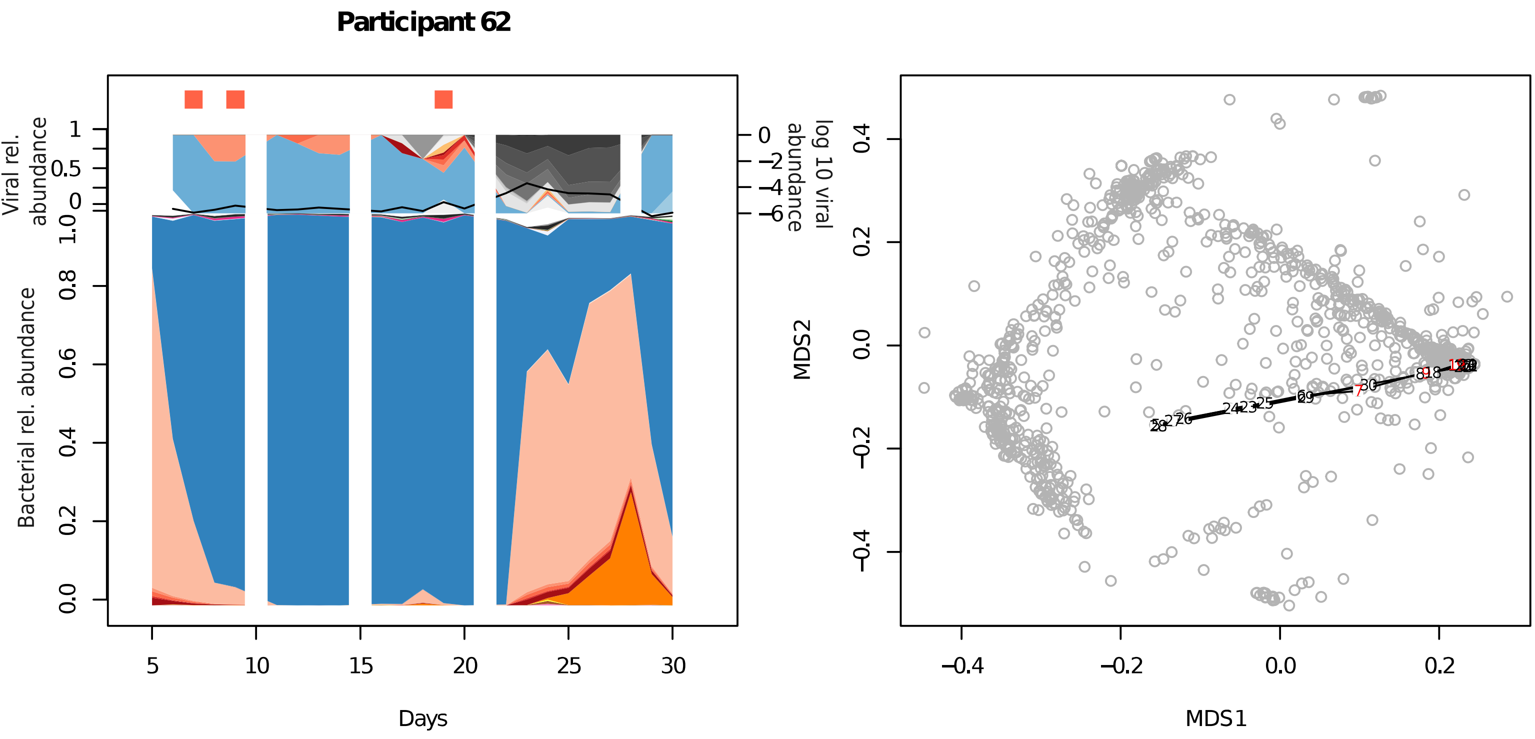
Participant 62
Viral rel. abundance
Bacterial rel. abundance
log 10 viral abundance
Days
MDS2
MDS1

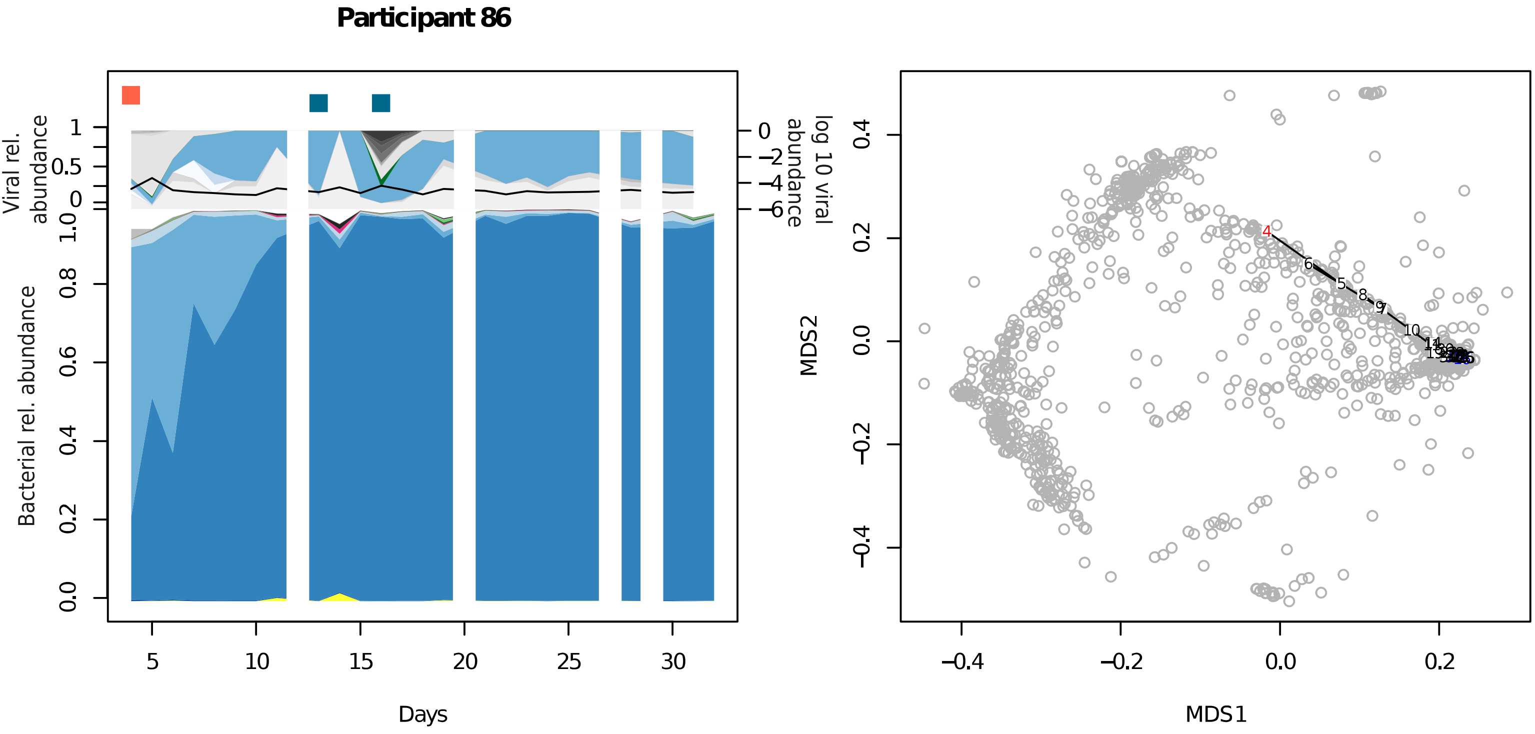
Participant 86
Viral rel. abundance
Bacterial rel. abundance
log 10 viral abundance
Days
MDS2
MDS1

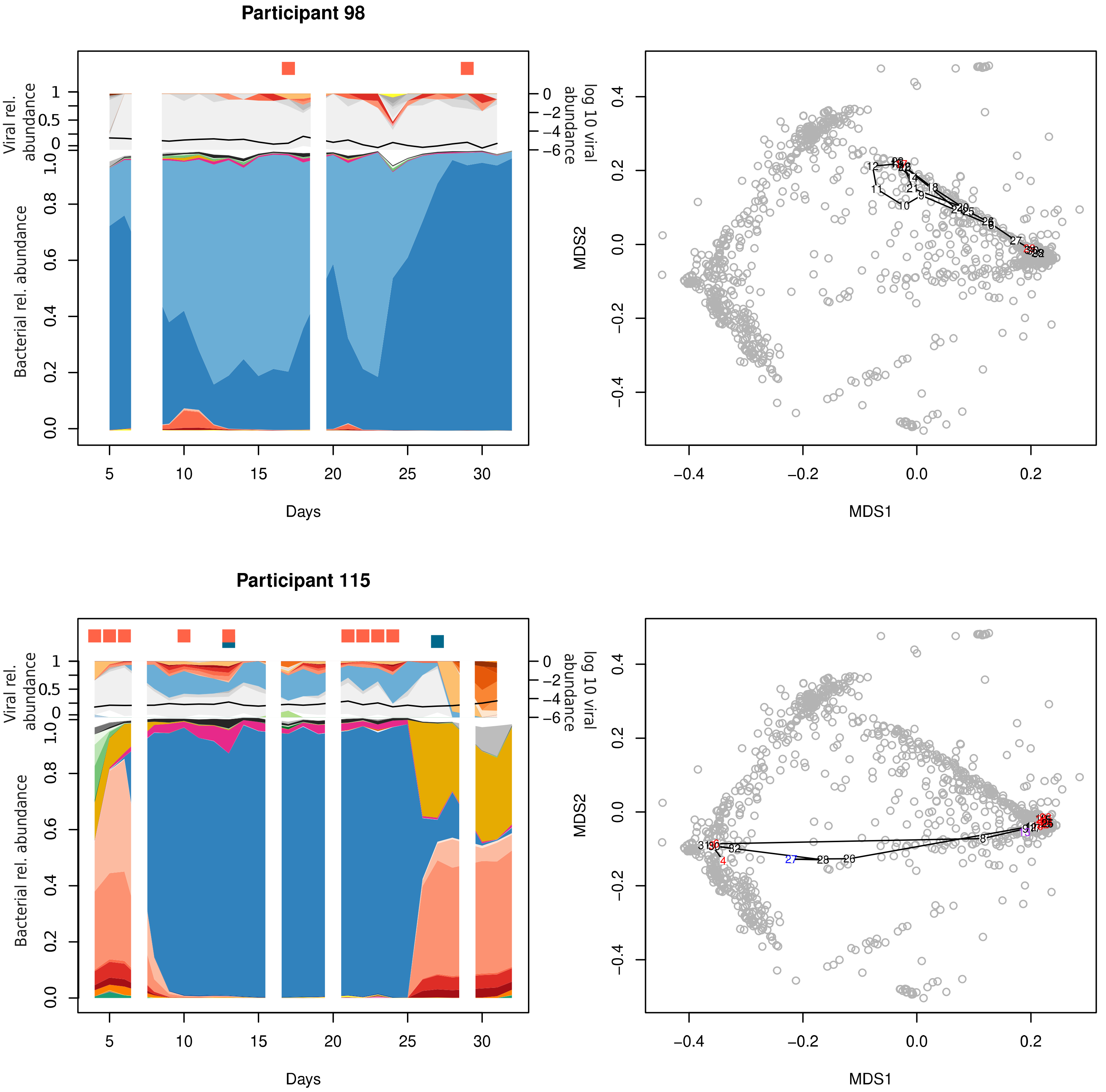

Participant 98
Participant 115
Viral rel. abundance
Bacterial rel. abundance
log 10 viral abundance
Days
MDS1
MDS2

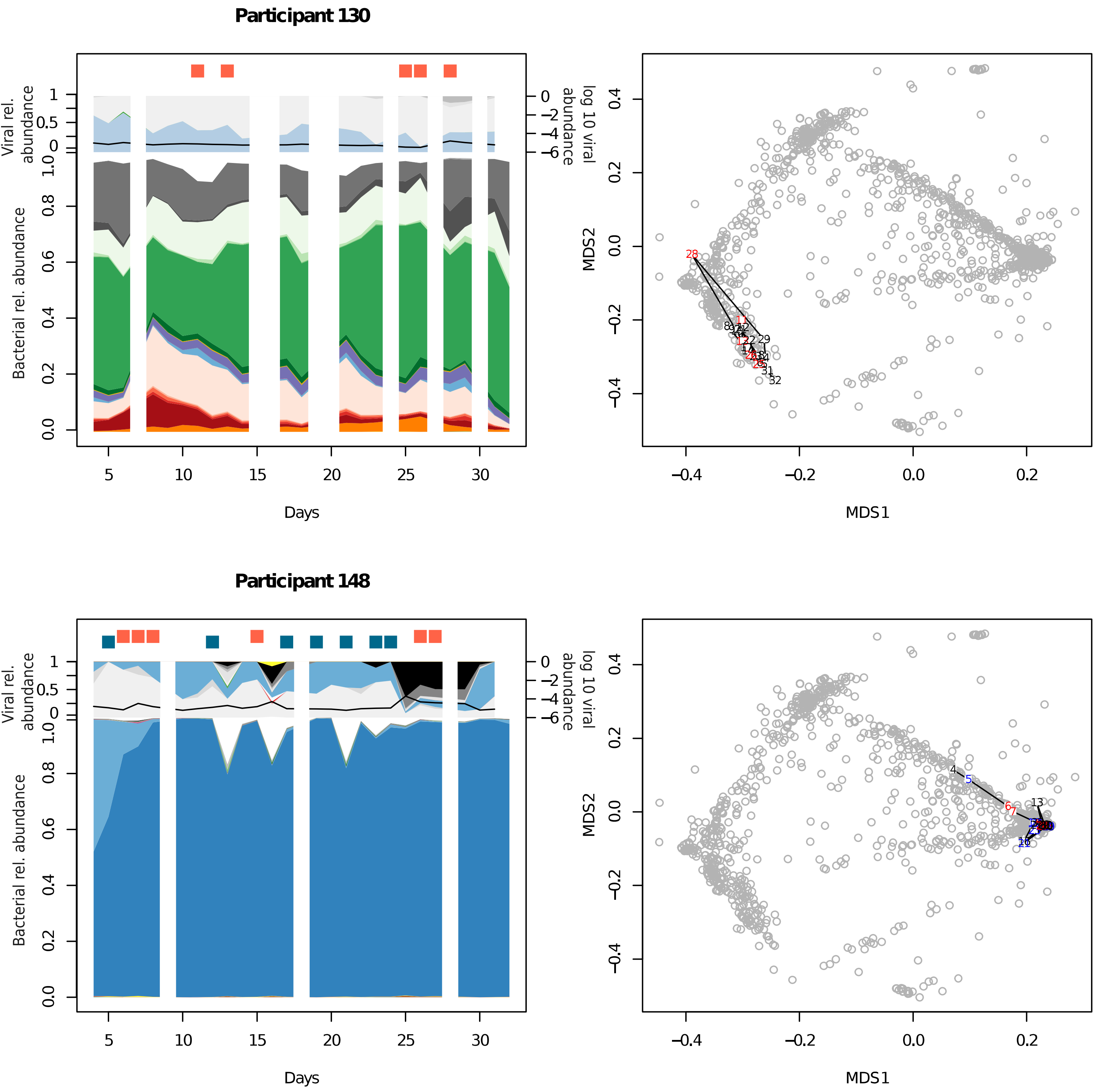

Participant 130
Participant 148
Viral rel. abundance
Bacterial rel. abundance
log 10 viral abundance
Days
MDS1
MDS2

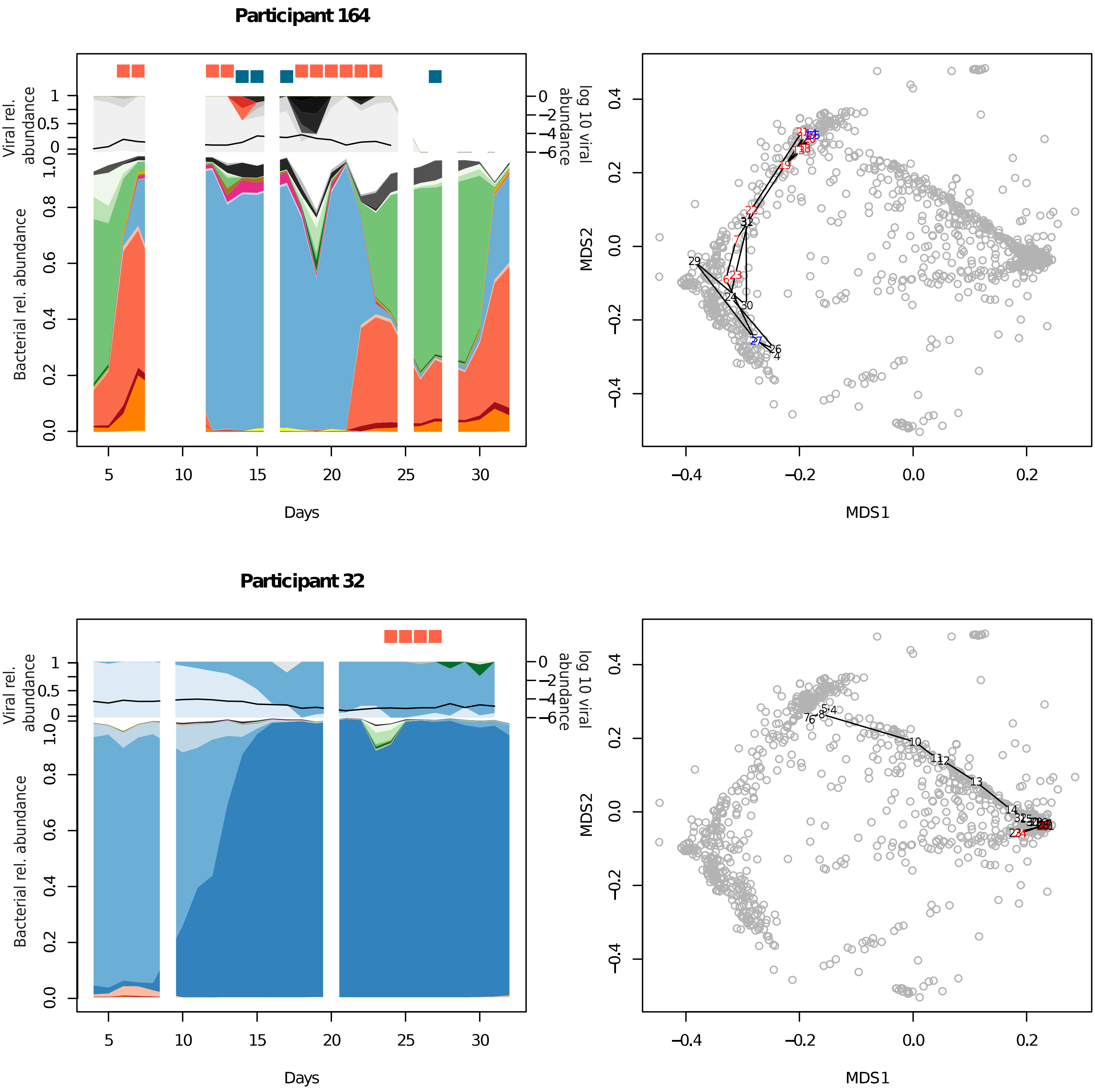
Participant 164
Participant 32
Viral rel. abundance
Bacterial rel. abundance
log 10 viral abundance
Days
MDS1
MDS2
0.0
0.2
0.4
0.6
0.8
1.0
0.5
0
1
−2
−4
−6
5
10
15
20
25
30
−0.4
−0.2
0.0
0.2
0.4

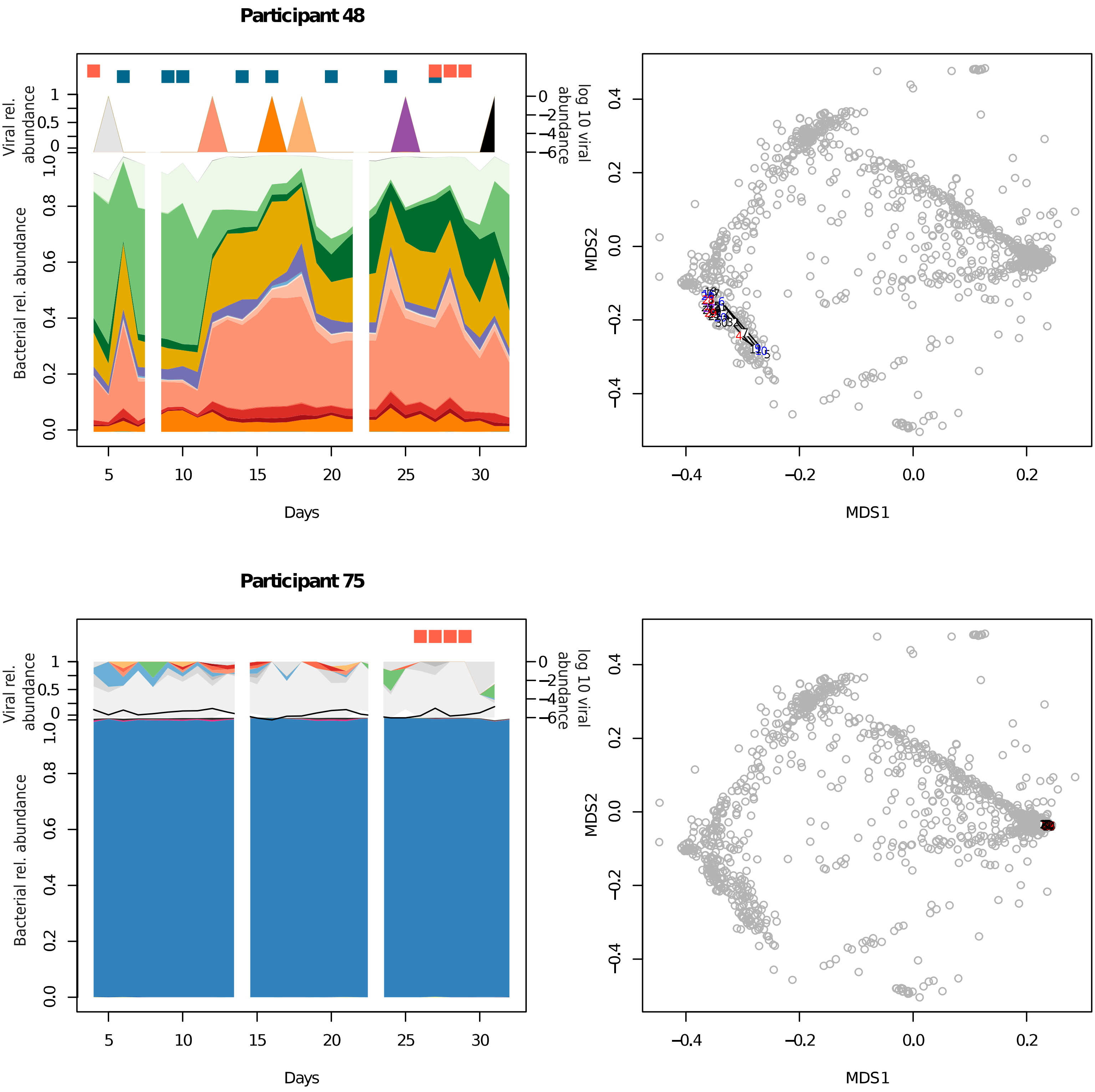

Participant 48
Viral rel. abundance
Bacterial rel. abundance
log 10 viral abundance
Days
MDS1
MDS2
Participant 75
Viral rel. abundance
Bacterial rel. abundance
log 10 viral abundance
Days
MDS1
MDS2

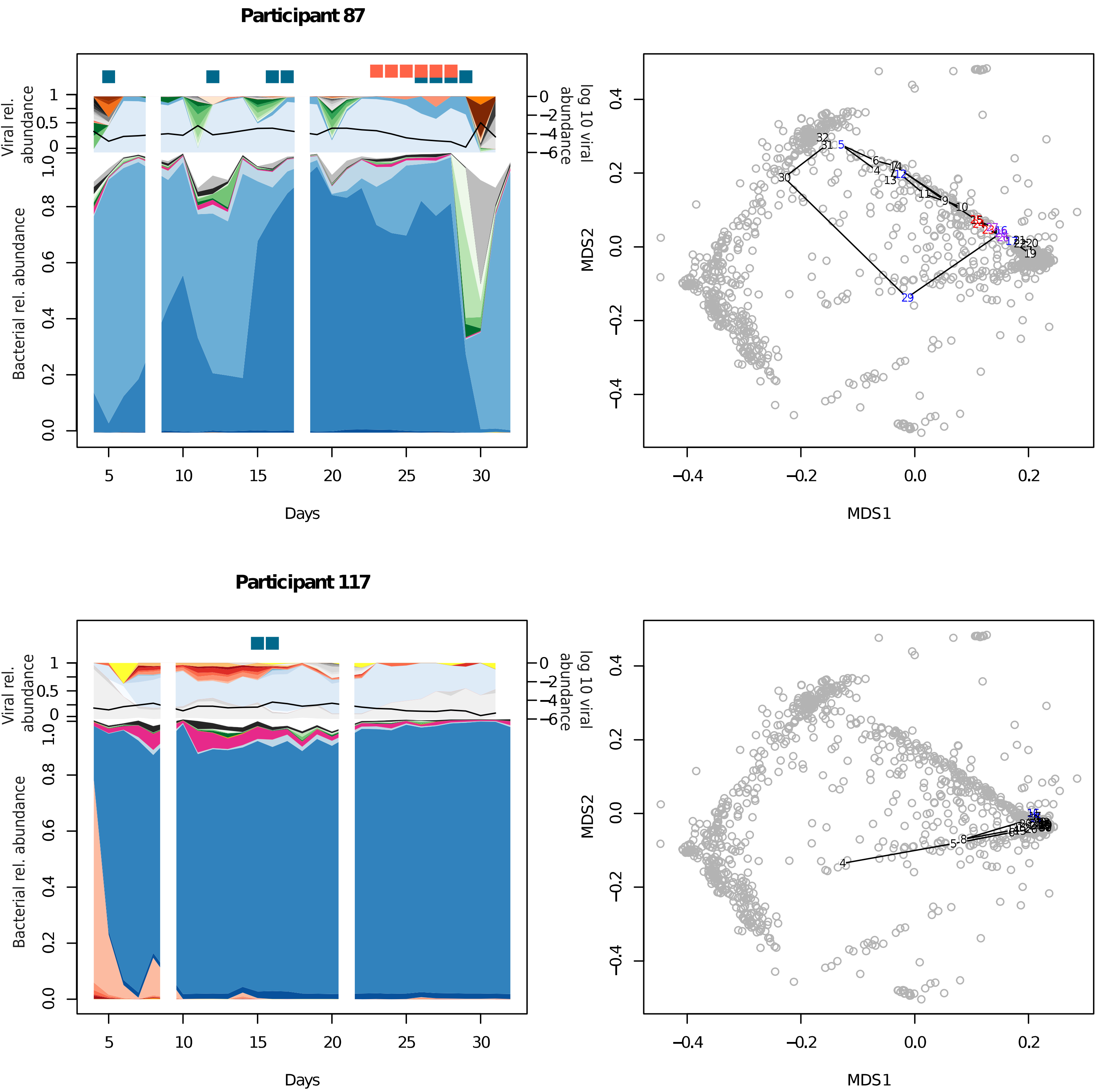
Participant 87
Viral rel. abundance
Bacterial rel. abundance
log 10 viral abundance
Days
MDS1
MDS2
Participant 117
Viral rel. abundance
Bacterial rel. abundance
log 10 viral abundance
Days
MDS1
MDS2

Participant 137

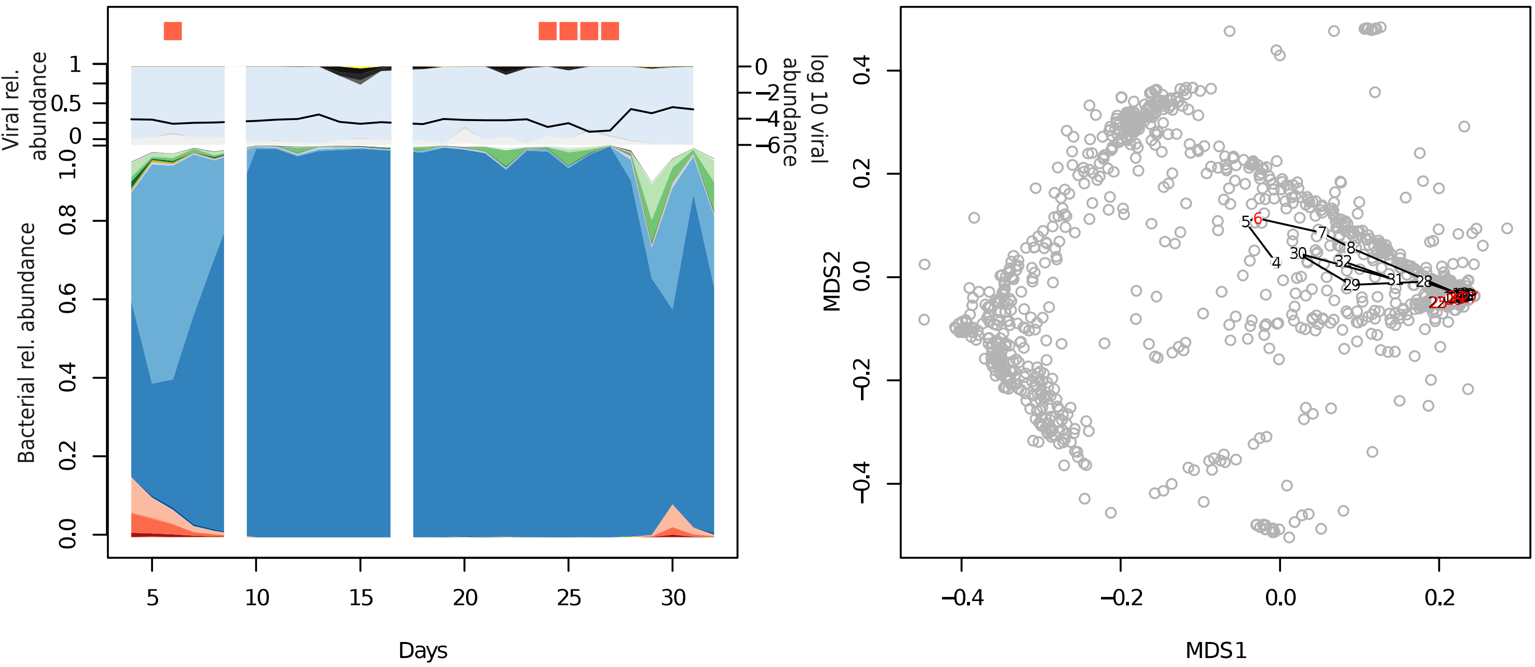

Participant 151

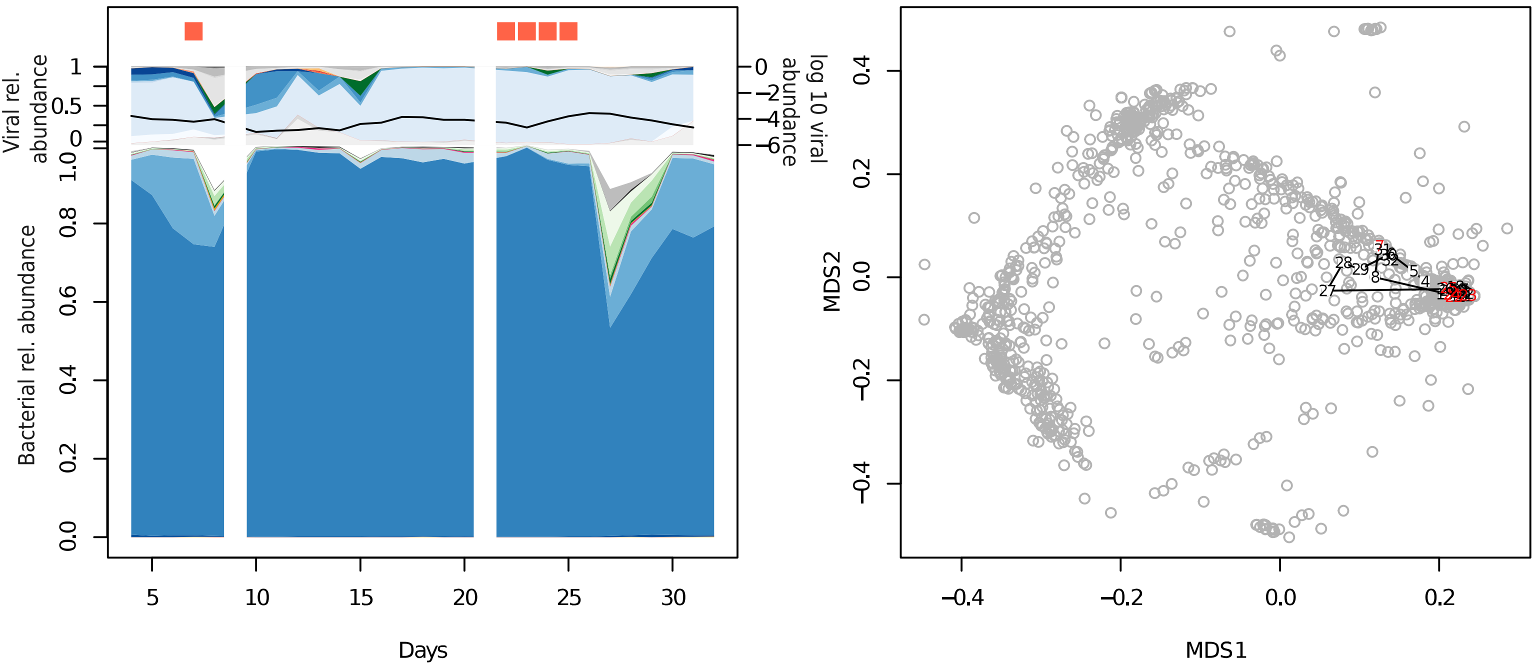

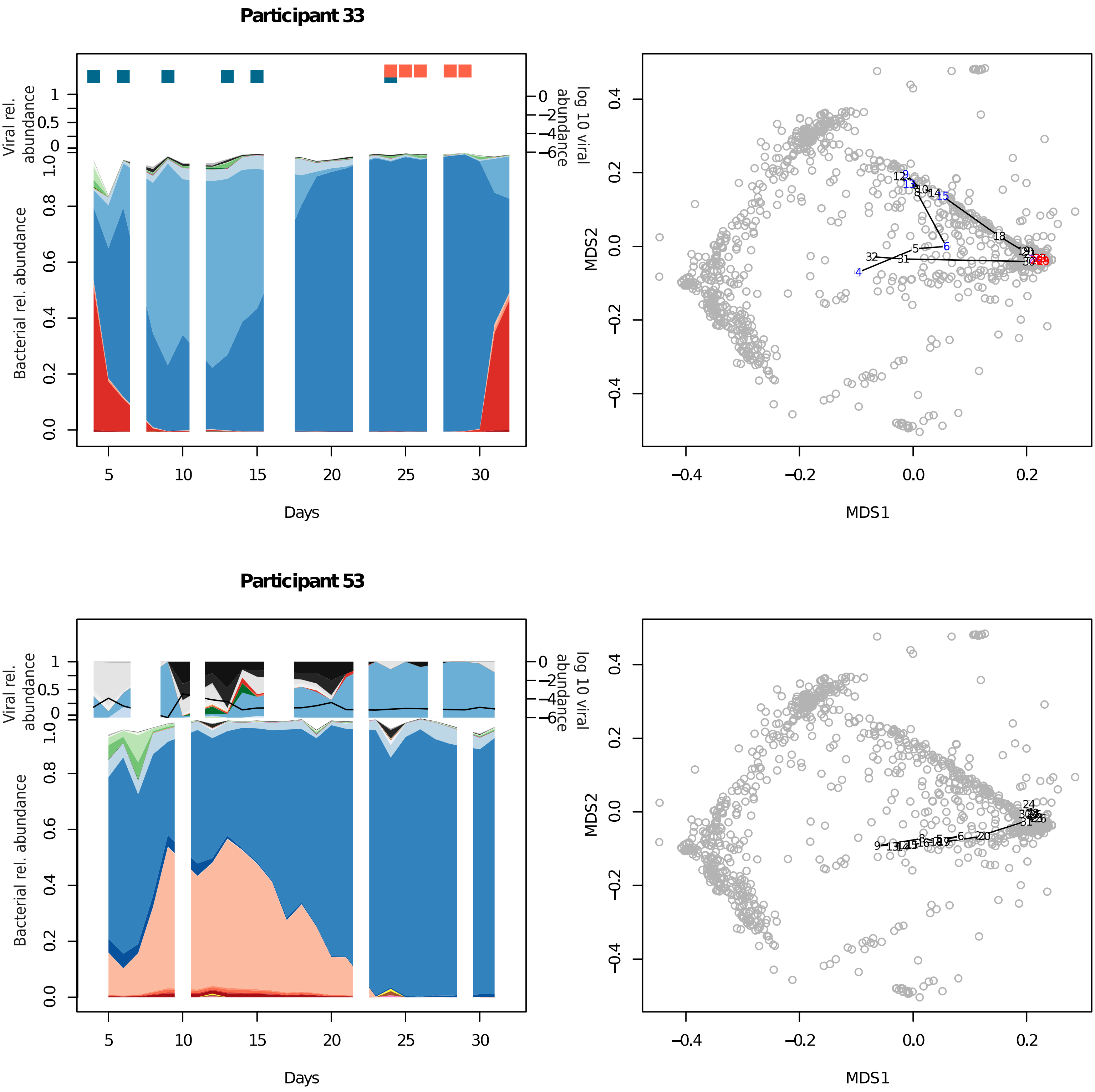
Participant 33
Viral rel. abundance
Bacterial rel. abundance
log 10 viral abundance
Days
MDS1
MDS2
Participant 53
Viral rel. abundance
Bacterial rel. abundance
log 10 viral abundance
Days
MDS1
MDS2

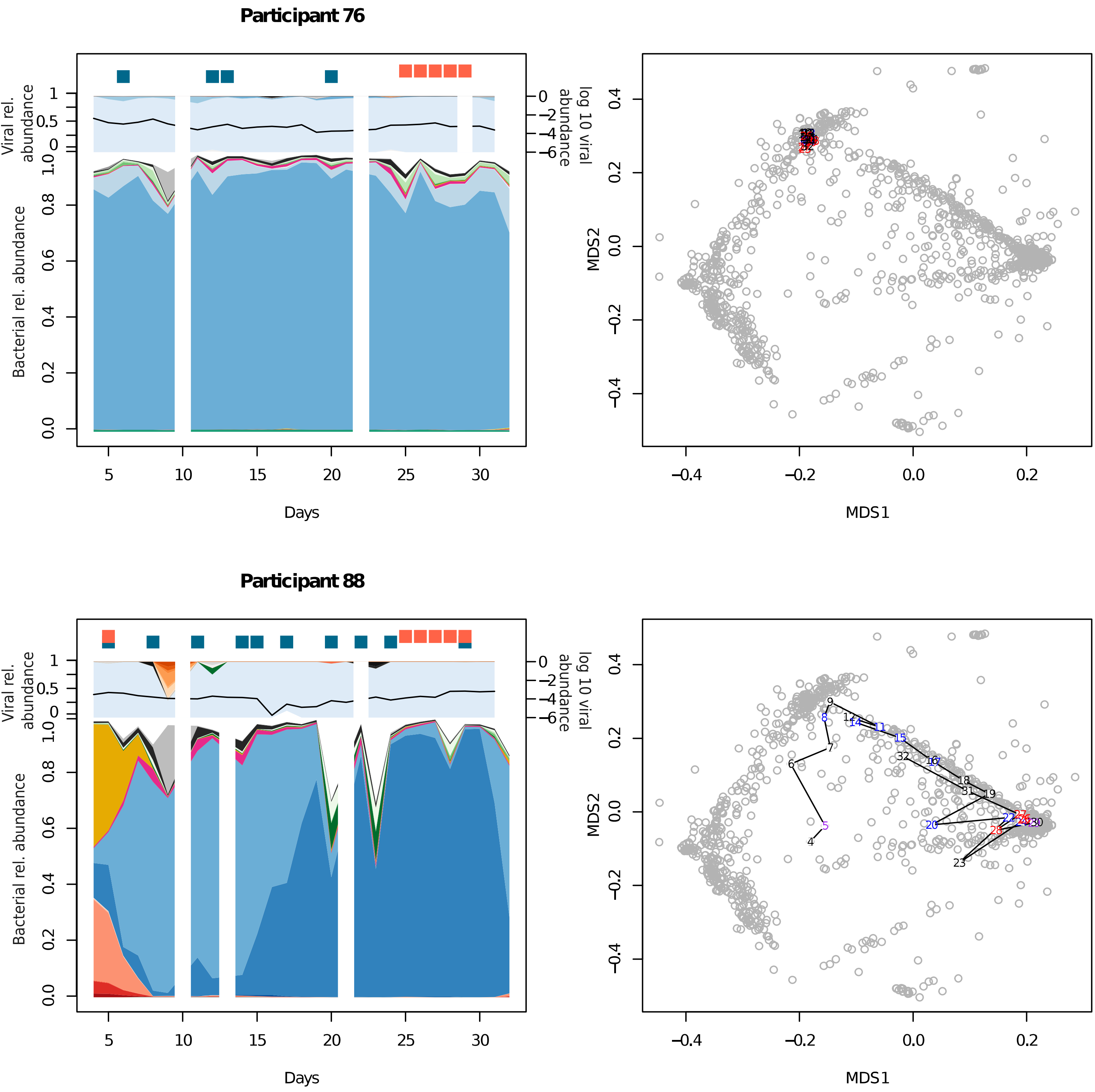

Participant 76
Viral rel. abundance
Bacterial rel. abundance
log 10 viral abundance
Days
MDS1
MDS2
Participant 88
Viral rel. abundance
Bacterial rel. abundance
log 10 viral abundance
Days
MDS1
MDS2

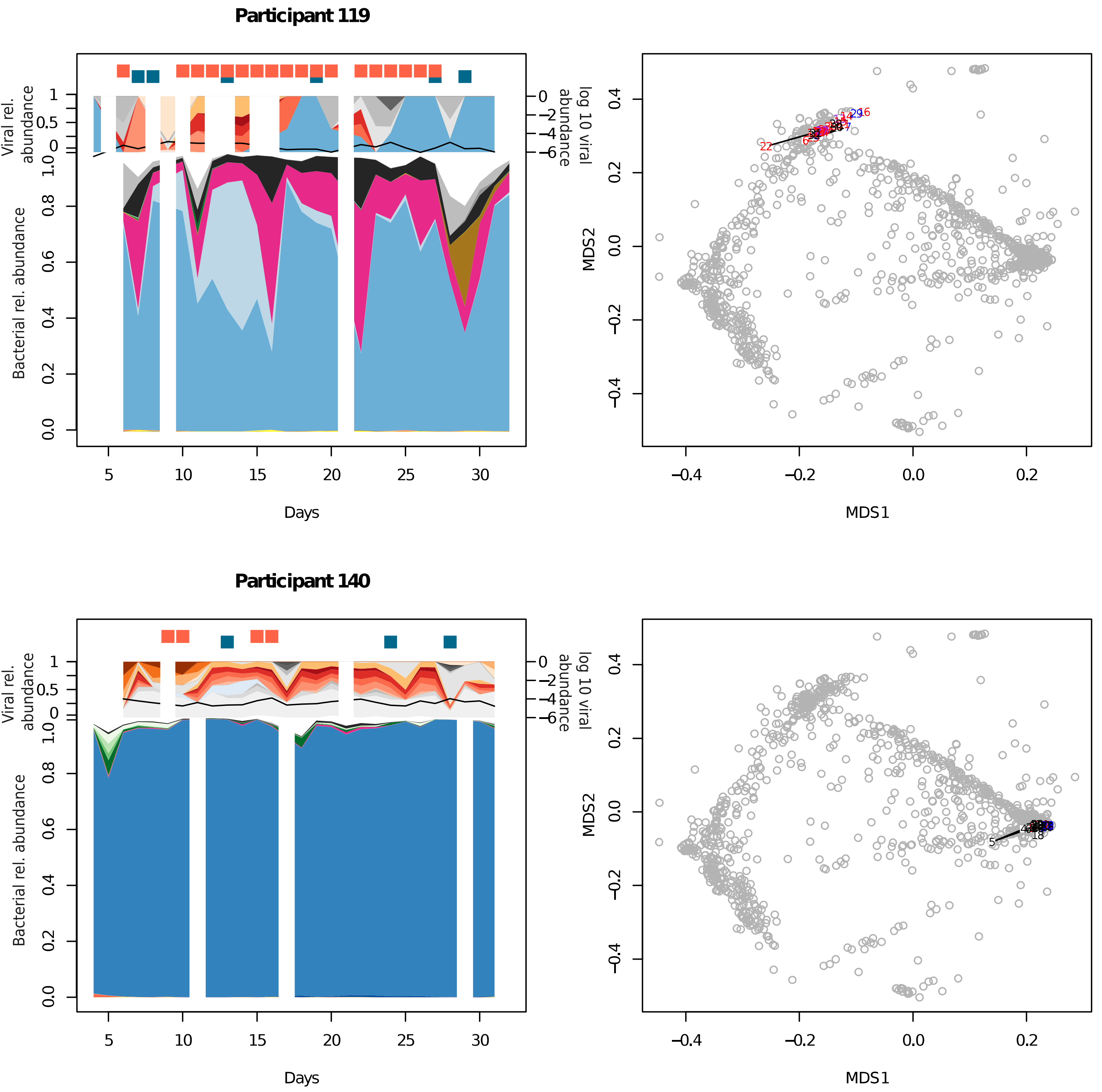

Participant 119
Viral rel. abundance
Bacterial rel. abundance
log 10 viral abundance
Days
MDS1
MDS2
Participant 140
Viral rel. abundance
Bacterial rel. abundance
log 10 viral abundance
Days
MDS1
MDS2

Participant 153

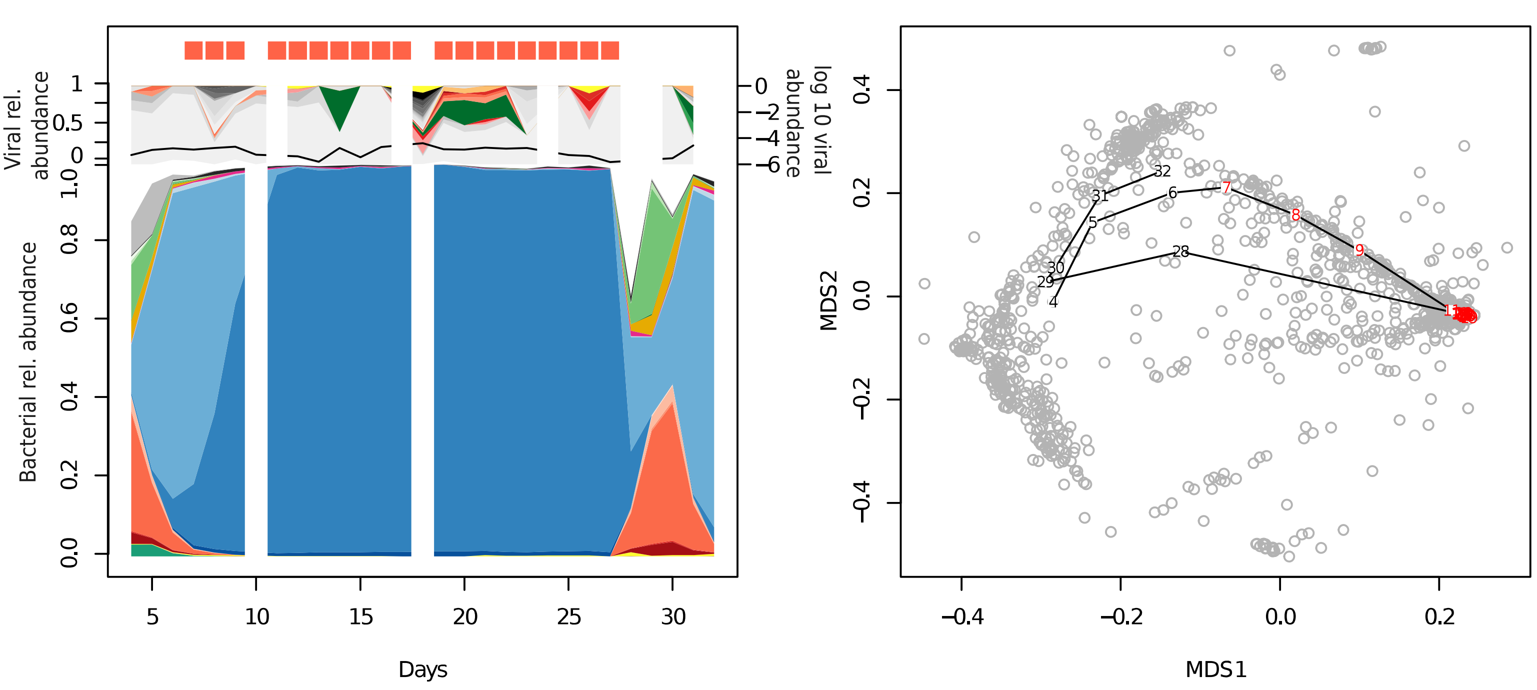

Participant 34

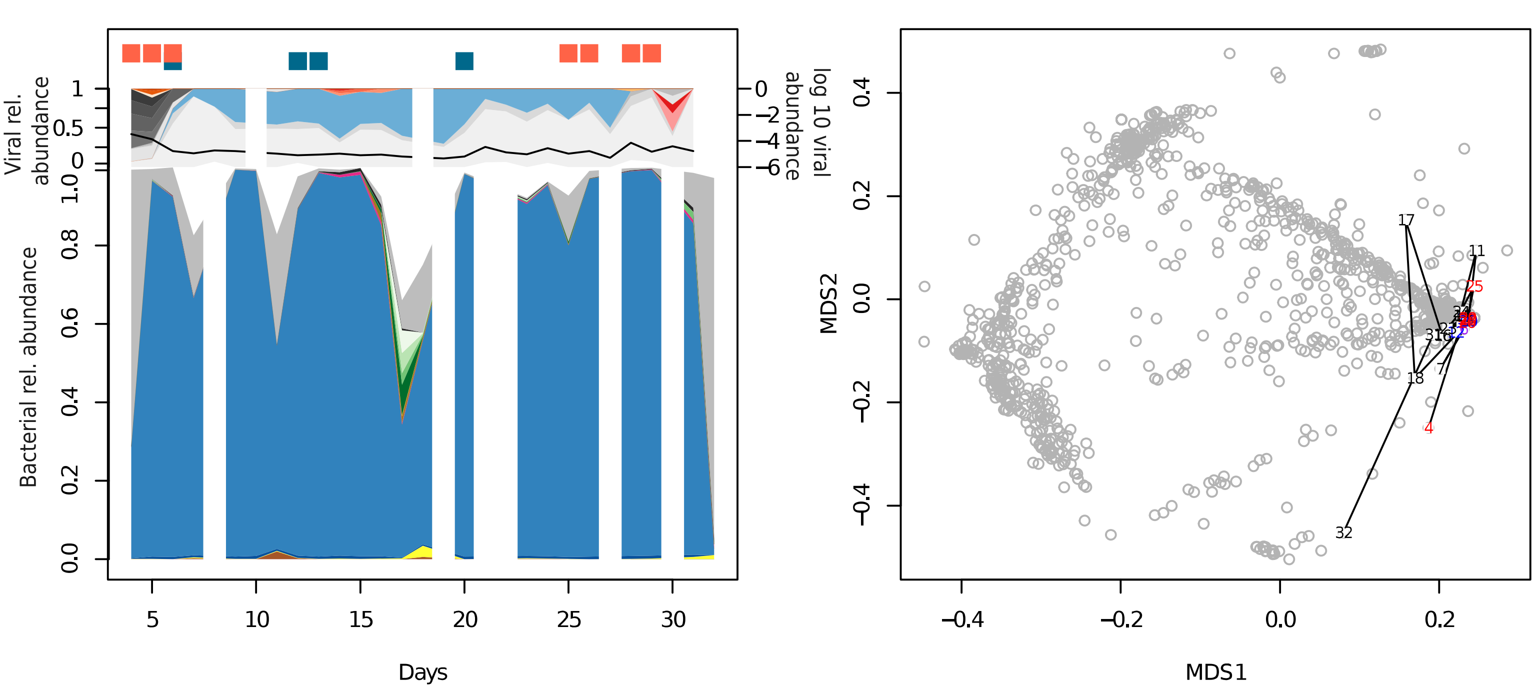

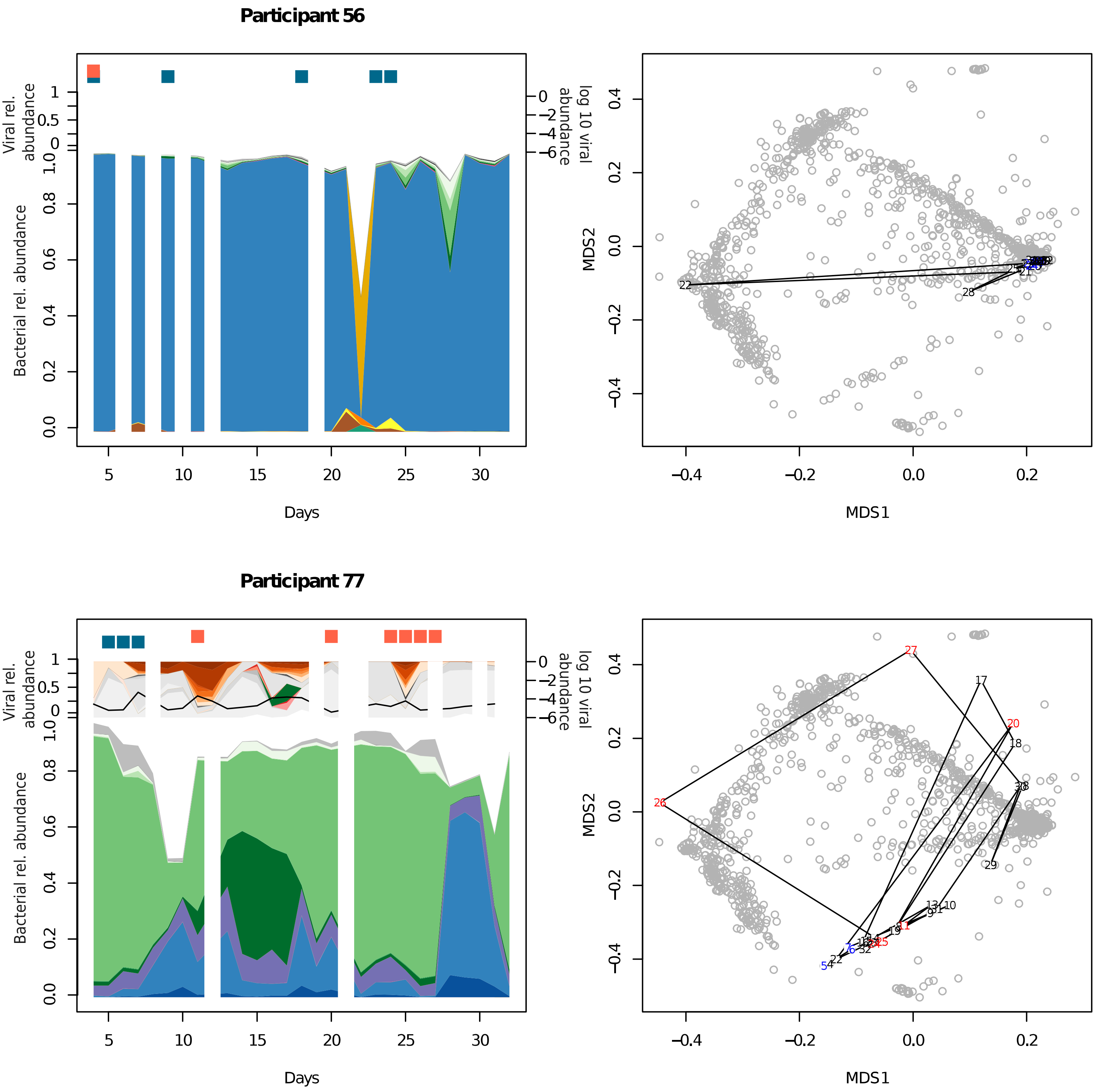
Participant 56
Viral rel. abundance
Bacterial rel. abundance
log 10 viral abundance
Days
MDS1
MDS2
Participant 77
Viral rel. abundance
Bacterial rel. abundance
log 10 viral abundance
Days
MDS1
MDS2

Participant 93

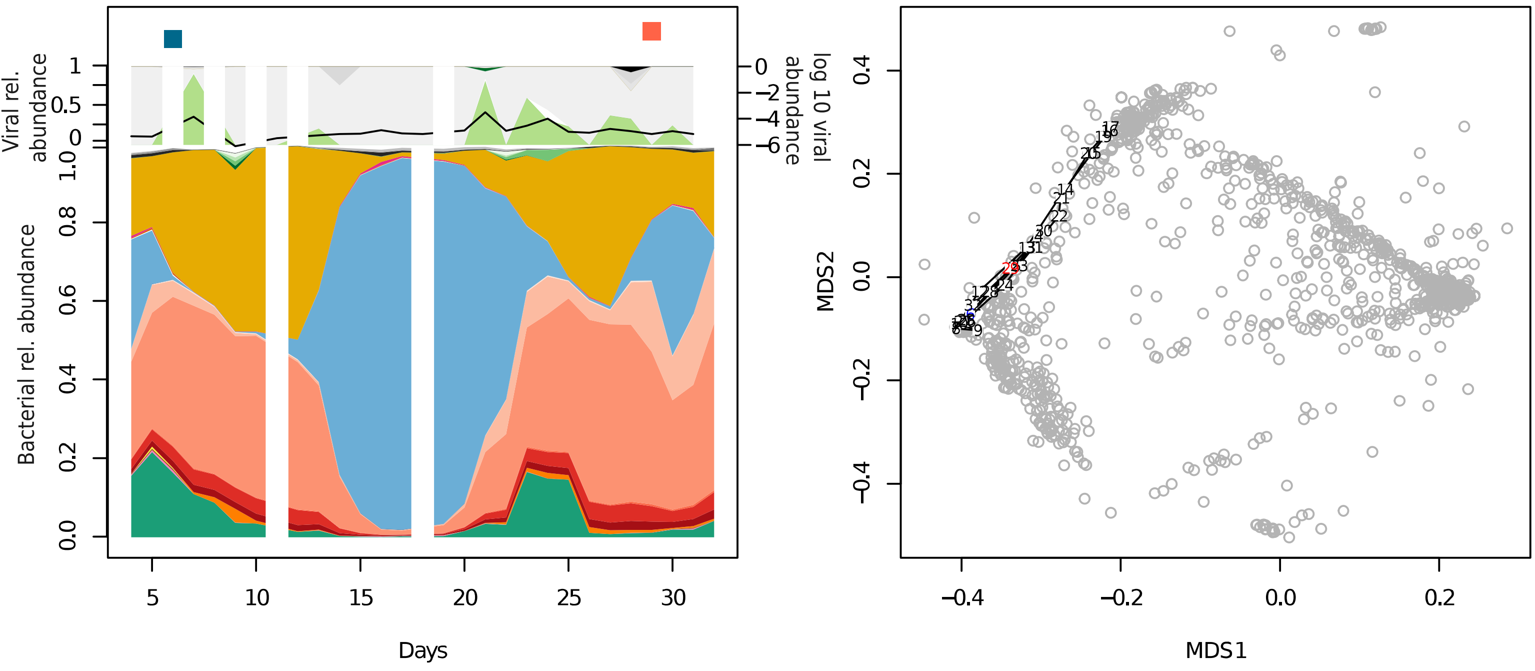

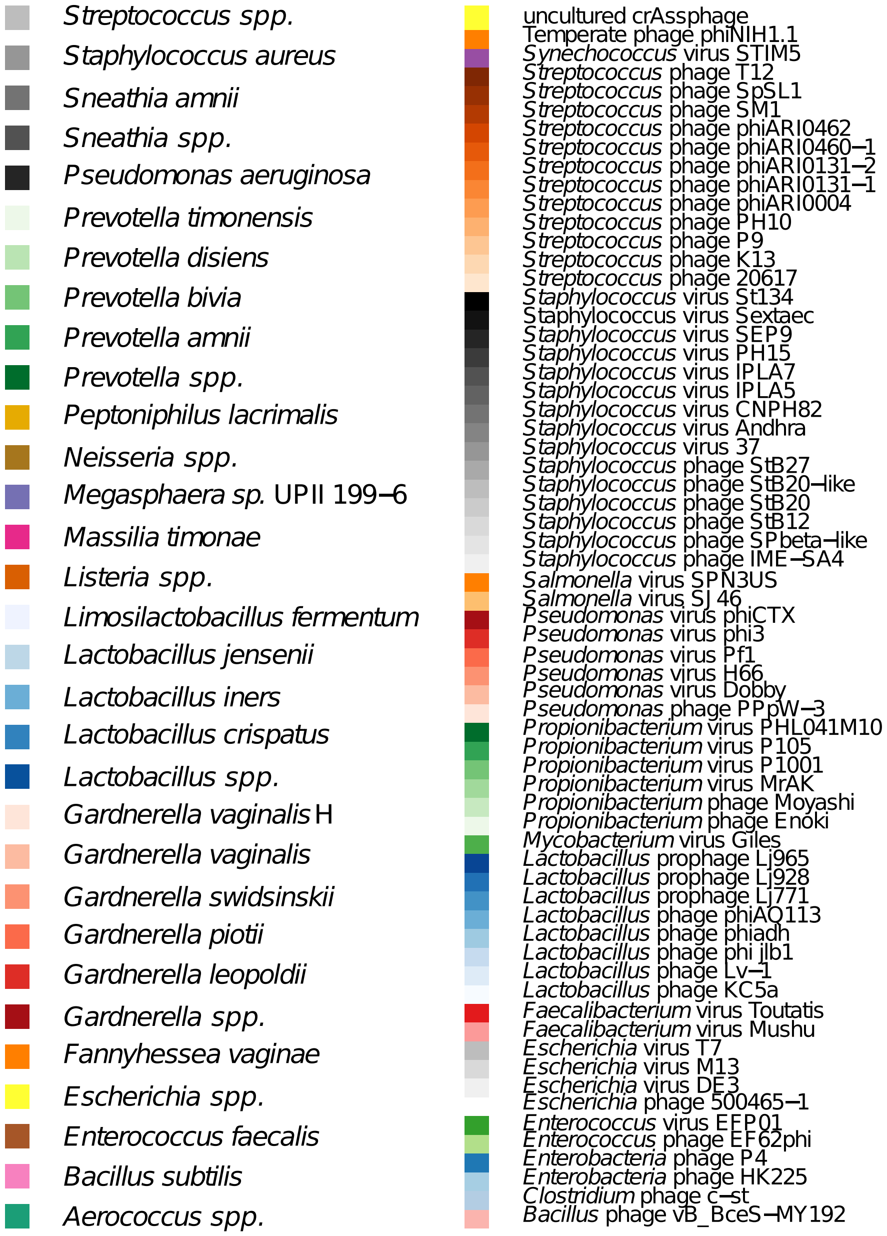

Streptococcus spp.
Staphylococcus aureus
Sneathia amnii
Sneathia spp.
Pseudomonas aeruginosa
Prevotella timonensis
Prevotella disiens
Prevotella bivia
Prevotella amnii
Prevotella spp.
Peptoniphilus lacrimalis
Neisseria spp.
Megasphaera sp. UPII 199−6
Massilia timonae
Listeria spp.
Limosilactobacillus fermentum
Lactobacillus jensenii
Lactobacillus iners
Lactobacillus crispatus
Lactobacillus spp.
Gardnerella vaginalis H
Gardnerella vaginalis
Gardnerella swidsinskii
Gardnerella piotii
Gardnerella leopoldii
Gardnerella spp.
Fannyhessea vaginae
Escherichia spp.
Enterococcus faecalis
Bacillus subtilis
Aerococcus spp.
uncultured crAssphage
Temperate phage phiNIH1.1
Synechococcus virus STIM5
Streptococcus phage T12
Streptococcus phage SpSL1
Streptococcus phage SM1
Streptococcus phage phiARI0462
Streptococcus phage phiARI0460−1
Streptococcus phage phiARI0131−2
Streptococcus phage phiARI0131−1
Streptococcus phage phiARI0004
Streptococcus phage PH10
Streptococcus phage P9
Streptococcus phage K13
Streptococcus phage 20617
Staphylococcus virus St134
Staphylococcus virus Sextaec
Staphylococcus virus SEP9
Staphylococcus virus PH15
Staphylococcus virus IPLA7
Staphylococcus virus IPLA5
Staphylococcus virus CNPH82
Staphylococcus virus Andhra
Staphylococcus virus 37
Staphylococcus phage StB27
Staphylococcus phage StB20−like
Staphylococcus phage StB20
Staphylococcus phage StB12
Staphylococcus phage SPbeta−like
Staphylococcus phage IME−SA4
Salmonella virus SPN3US
Salmonella virus SJ46
Pseudomonas virus phiCTX
Pseudomonas virus phi3
Pseudomonas virus Pf1
Pseudomonas virus H66
Pseudomonas virus Dobby
Pseudomonas phage PPpW−3
Propionibacterium virus PHL041M10
Propionibacterium virus P105
Propionibacterium virus P1001
Propionibacterium virus MrAK
Propionibacterium phage Moyashi
Propionibacterium phage Enoki
Mycobacterium virus Giles
Lactobacillus prophage Lj965
Lactobacillus prophage Lj928
Lactobacillus prophage Lj771
Lactobacillus phage phiAQ113
Lactobacillus phage phiadh
Lactobacillus phage phi jlb1
Lactobacillus phage Lv−1
Lactobacillus phage KC5a
Faecalibacterium virus Toutatis
Faecalibacterium virus Mushu
Escherichia virus T7
Escherichia virus M13
Escherichia virus DE3
Escherichia phage 500465−1
Enterococcus virus EFP01
Enterococcus phage EF62phi
Enterobacteria phage P4
Enterobacteria phage HK225
Clostridium phage c−st
Bacillus phage vB_BceS−MY192

Supplementary figure 2: CST distribution and time-series dynamics for the 16S samples

CSTs are shown as colored dots as per the legend in the page 31. The outline of each box depicts the assignment to vaginal community dynamics. Missing samples are omitted. Bleedings are marked as red dots. Blue: constant eubiotic. Green: menses-related dysbiotic. Yellow: unstable. Red: constant dysbiotic.

Figure S2 extends for two pages, the first one with the data, and the second with the colour key.

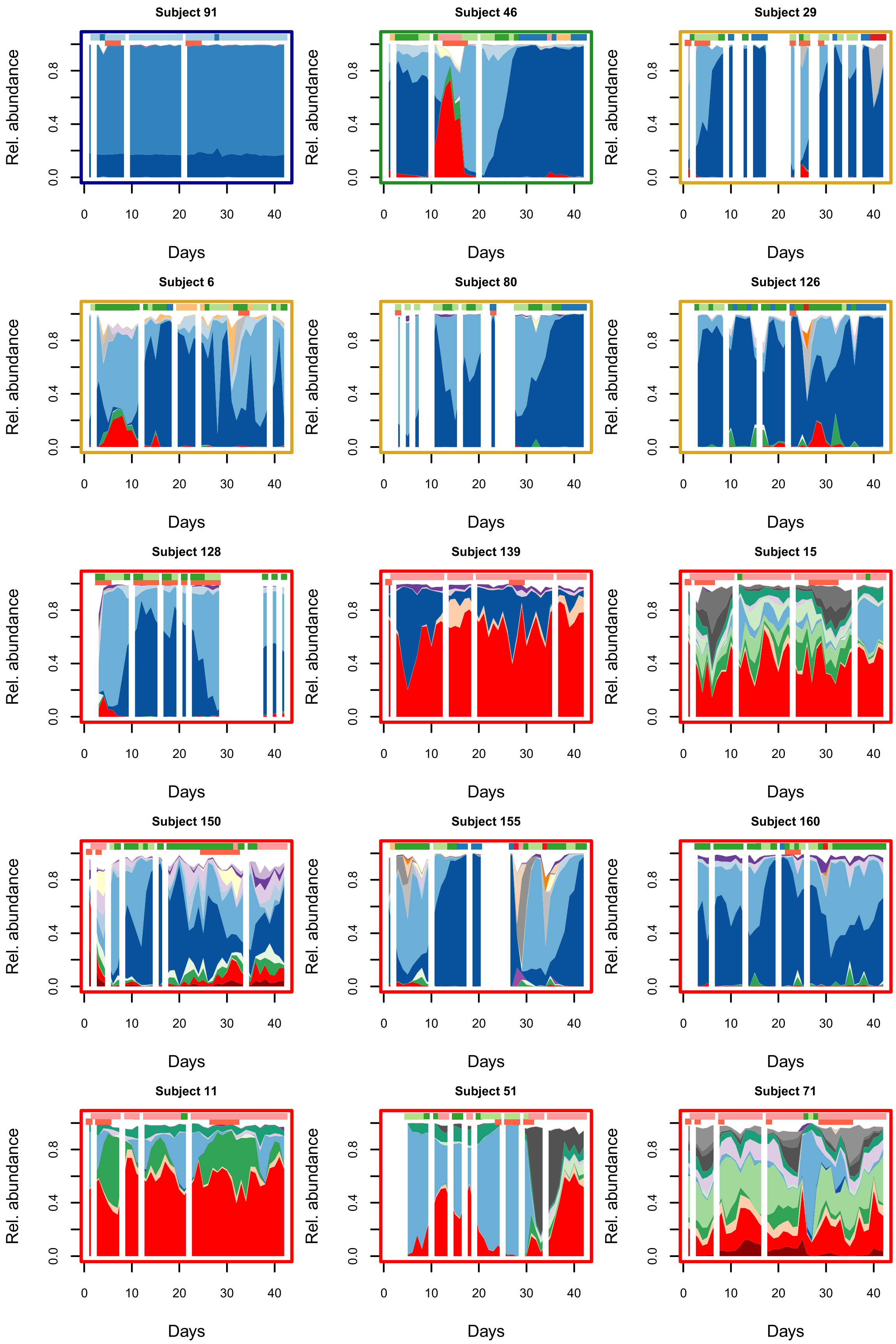
Subject 91
Subject 46
Subject 29
Subject 6
Subject 80
Subject 126
Subject 128
Subject 139
Subject 15
Subject 150
Subject 155
Subject 160
Subject 11
Subject 51
Subject 71
Rel. abundance
0.0
0.4
0.8
0
10
20
30
40
Days

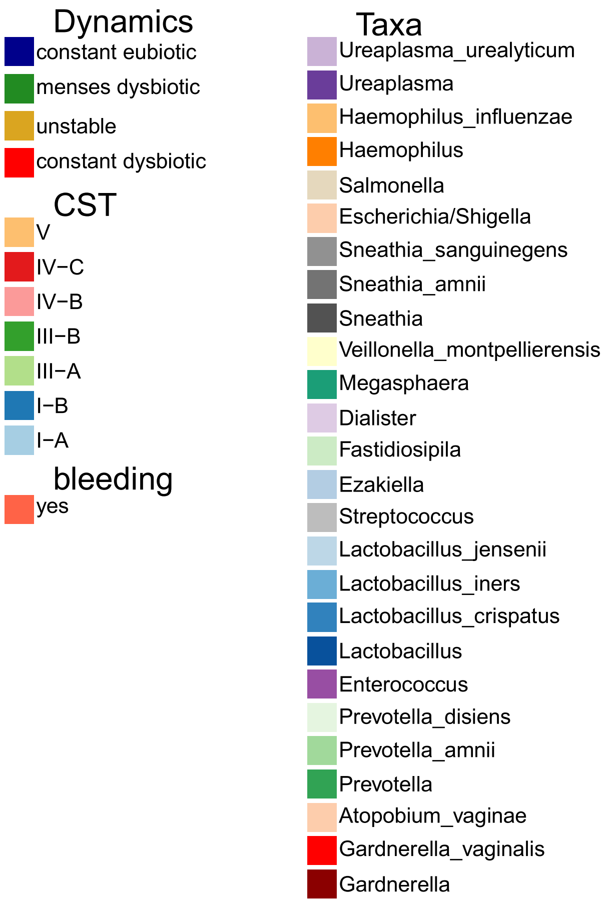
Dynamics
constant eubiotic
menses dysbiotic
unstable
constant dysbiotic
CST
V
IV−C
IV−B
III−B
III−A
I−B
I−A
bleeding
yes
Taxa
Ureaplasma_urealyticum
Ureaplasma
Haemophilus_influenzae
Haemophilus
Salmonella
Escherichia/Shigella
Sneathia_sanguinegens
Sneathia_amnii
Sneathia
Veillonella_montpellierensis
Megasphaera
Dialister
Fastidiosipila
Ezakiella
Streptococcus
Lactobacillus_jensenii
Lactobacillus_iners
Lactobacillus_crispatus
Lactobacillus
Enterococcus
Prevotella_disiens
Prevotella_amnii
Prevotella
Atopobium_vaginae
Gardnerella_vaginalis
Gardnerella

Supplementary figure 3: CST distribution and time-series dynamics for the shotgun samples

CSTs are marked as colored dots above the taxonomic profiles as per the legend. The outline of each box depicts its dynamic group. Bleedings are marked as light red dots. Missing samples are omitted. Blue: constant eubiotic. Green: menses-related dysbiotic. Yellow: Unstable. Red: constant dysbiotic.

Fig. S3 extends from page 32 to page 38. The color scheme is repeated in both these pages.

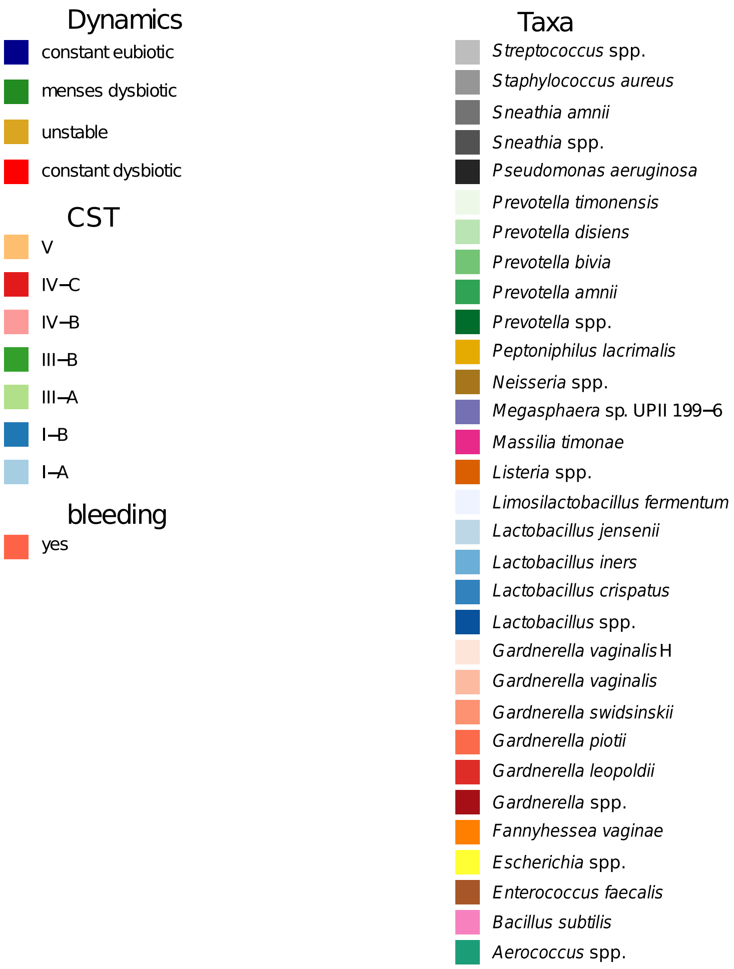
Dynamics
constant eubiotic
menses dysbiotic
unstable
constant dysbiotic
CST
V
IV–C
IV–B
III–B
III–A
I–B
I–A
bleeding
yes
Taxa
Streptococcus spp.
Staphylococcus aureus
Sneathia amnii
Sneathia spp.
Pseudomonas aeruginosa
Prevotella timonensis
Prevotella disiens
Prevotella bivia
Prevotella amnii
Prevotella spp.
Peptoniphilus lacrimalis
Neisseria spp.
Megasphaera sp. UPII 199–6
Massilia timonae
Listeria spp.
Limosilactobacillus fermentum
Lactobacillus jensenii
Lactobacillus iners
Lactobacillus crispatus
Lactobacillus spp.
Gardnerella vaginalis H
Gardnerella vaginalis
Gardnerella swidsinskii
Gardnerella piotii
Gardnerella leopoldii
Gardnerella spp.
Fannyhessea vaginae
Escherichia spp.
Enterococcus faecalis
Bacillus subtilis
Aerococcus spp.

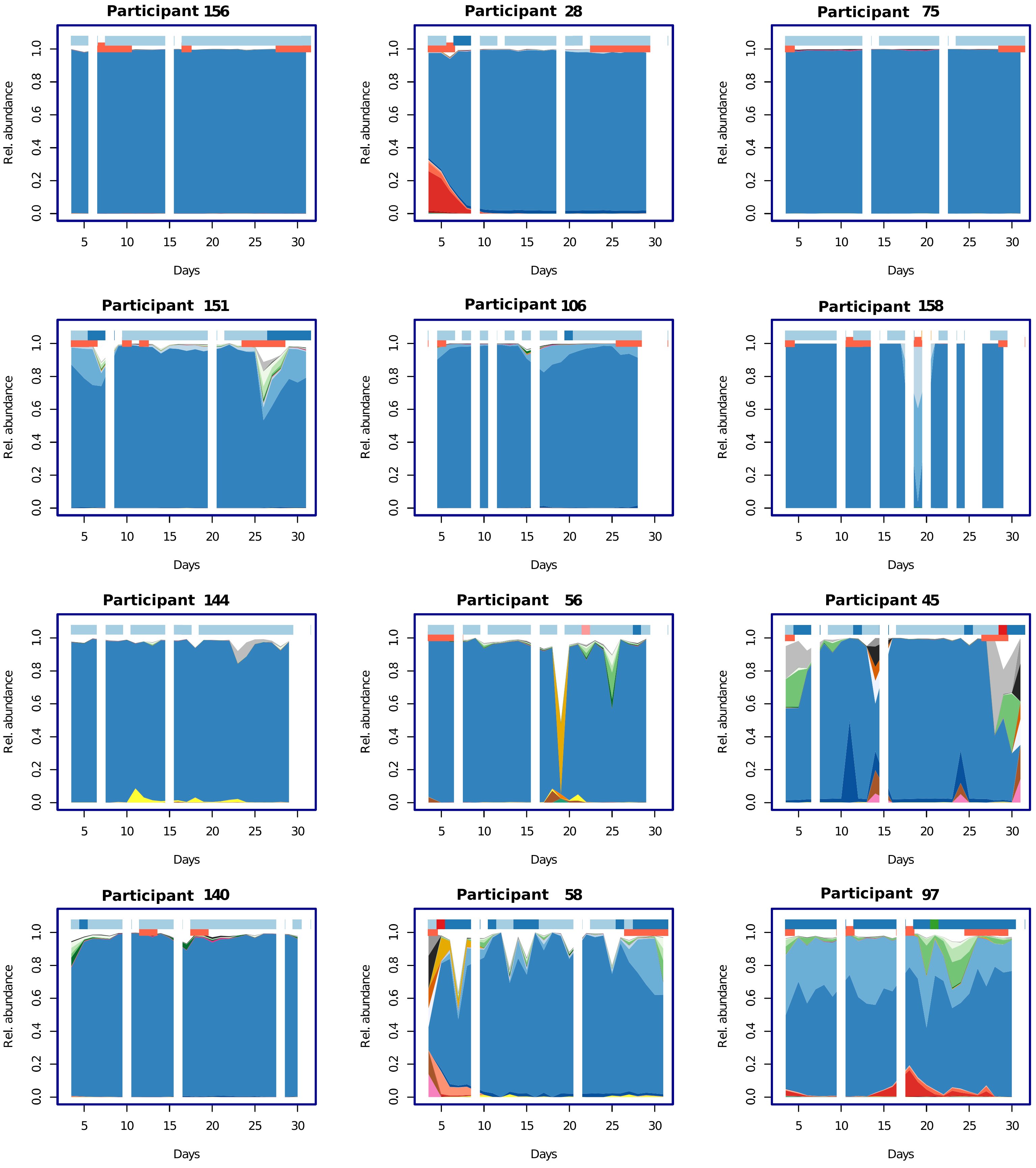
Participant 156
Participant 28
Participant 75
Participant 151
Participant 106
Participant 158
Participant 144
Participant 56
Participant 45
Participant 140
Participant 58
Participant 97
Rel. abundance
0.0
0.2
0.4
0.6
0.8
1.0
5
10
15
20
25
30
Days

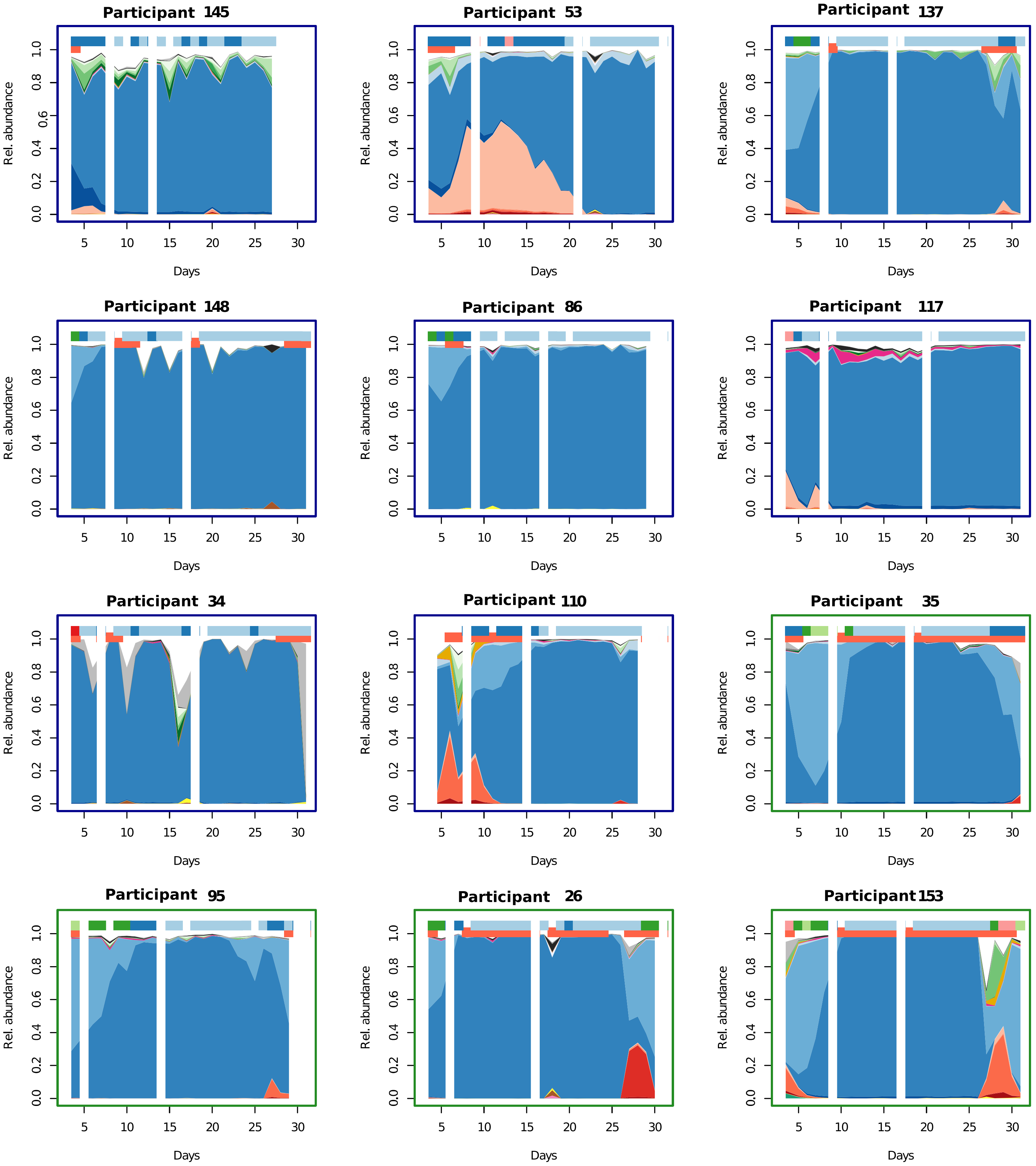
Participant 145
Participant 53
Participant 137
Participant 148
Participant 86
Participant 117
Participant 34
Participant 110
Participant 35
Participant 95
Participant 26
Participant 153
Rel. abundance
Days
0.0
0.2
0.4
0.6
0.8
1.0
5
10
15
20
25
30

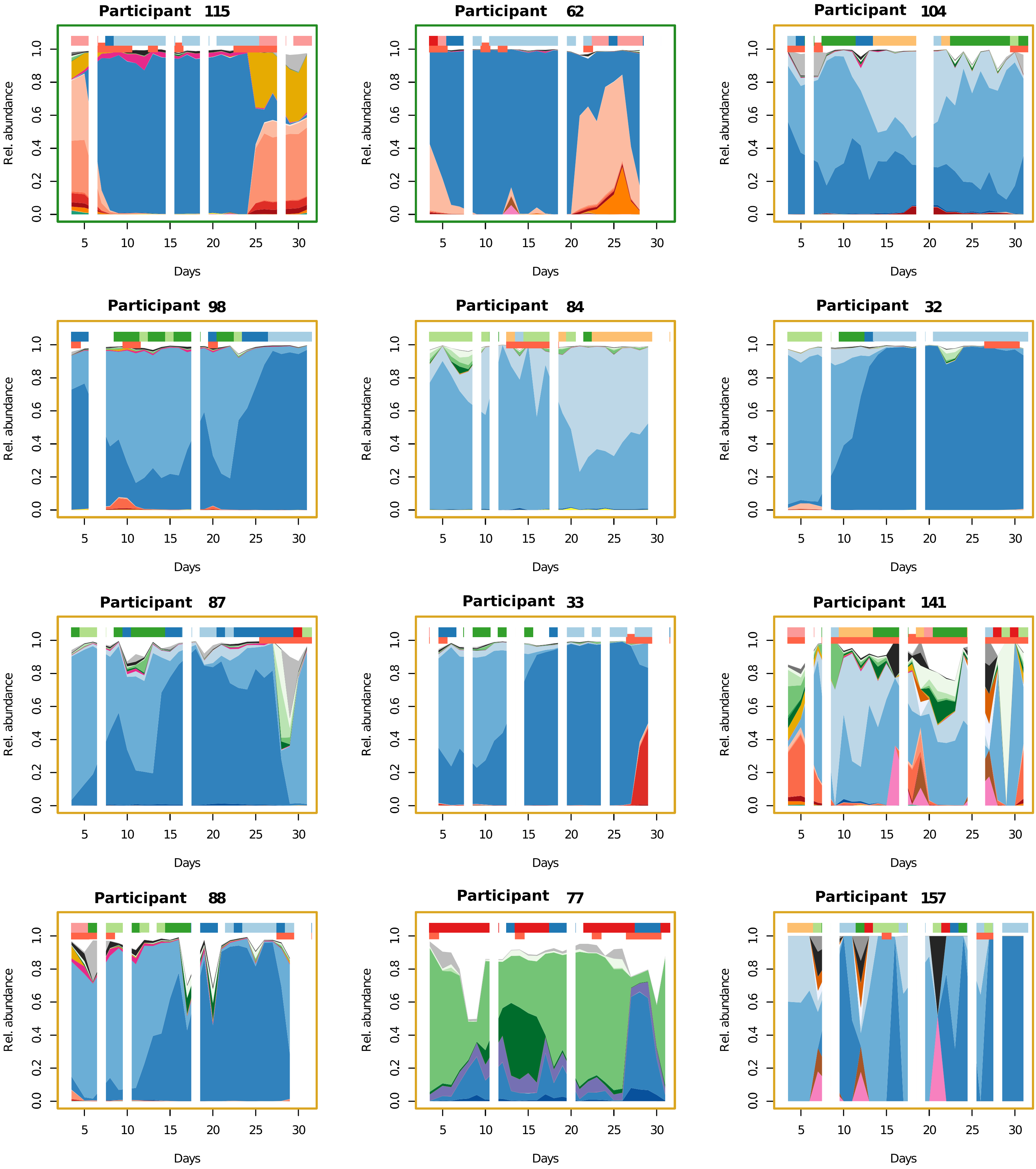
Participant 115
Participant 62
Participant 104
Participant 98
Participant 84
Participant 32
Participant 87
Participant 33
Participant 141
Participant 88
Participant 77
Participant 157
Rel. abundance
0.0
0.2
0.4
0.6
0.8
1.0
5
10
15
20
25
30
Days

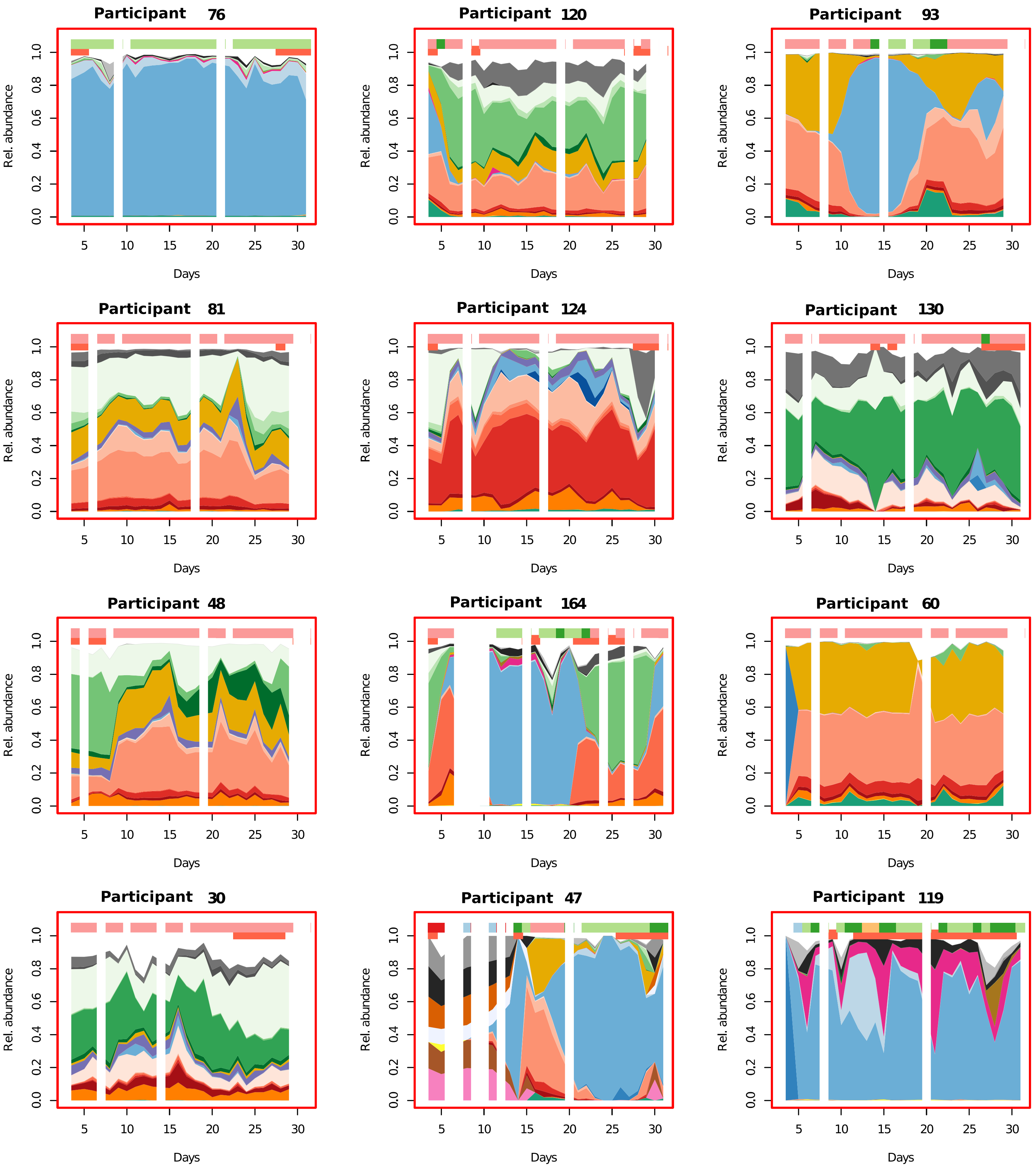
Participant 76
Participant 120
Participant 93
Participant 81
Participant 124
Participant 130
Participant 48
Participant 164
Participant 60
Participant 30
Participant 47
Participant 119
Rel. abundance
Days

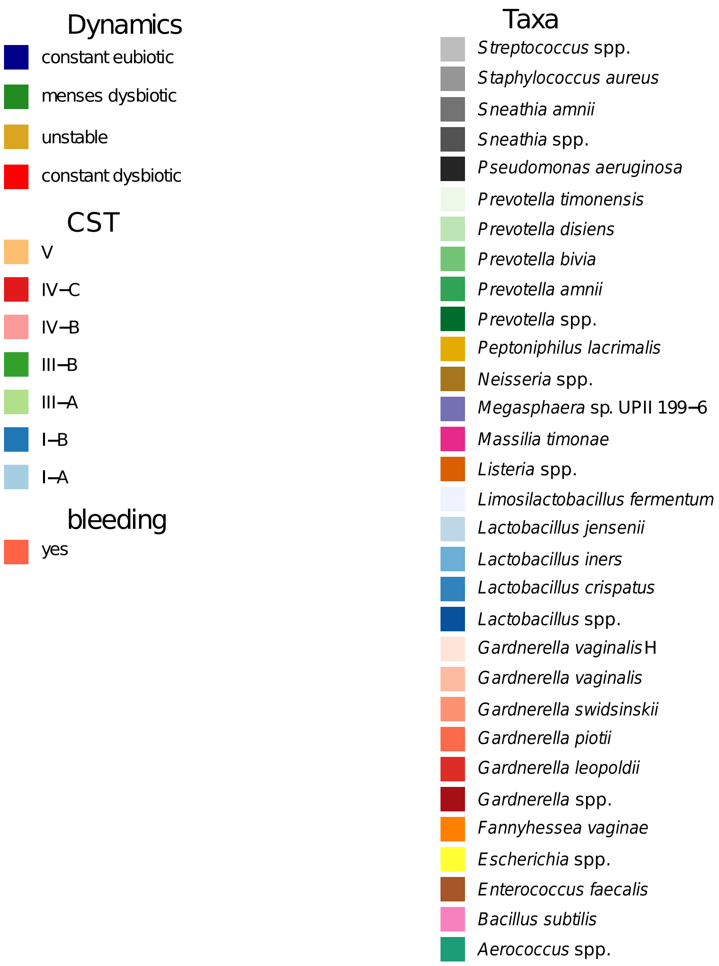
Dynamics
constant eubiotic
menses dysbiotic
unstable
constant dysbiotic
CST
V
IV−C
IV−B
III−B
III−A
I−B
I−A
bleeding
yes
Taxa
Streptococcus spp.
Staphylococcus aureus
Sneathia amnii
Sneathia spp.
Pseudomonas aeruginosa
Prevotella timonensis
Prevotella disiens
Prevotella bivia
Prevotella amnii
Prevotella spp.
Peptoniphilus lacrimalis
Neisseria spp.
Megasphaera sp. UPII 199−6
Massilia timonae
Listeria spp.
Limosilactobacillus fermentum
Lactobacillus jensenii
Lactobacillus iners
Lactobacillus crispatus
Lactobacillus spp.
Gardnerella vaginalis H
Gardnerella vaginalis
Gardnerella swidsinskii
Gardnerella piotii
Gardnerella leopoldii
Gardnerella spp.
Fannyhessea vaginae
Escherichia spp.
Enterococcus faecalis
Bacillus subtilis
Aerococcus spp.

Supplementary figure 4: Log-fold change of bacterial species in samples from CST-I

Samples in CST-IA and CST-IB from menses-related dysbiotic or unstable individuals were compared to constant eubiotic individuals. The heatmap shows the log-fold change of all significant differences. Gray fields represent no significant change.

This figure occupies a single page.

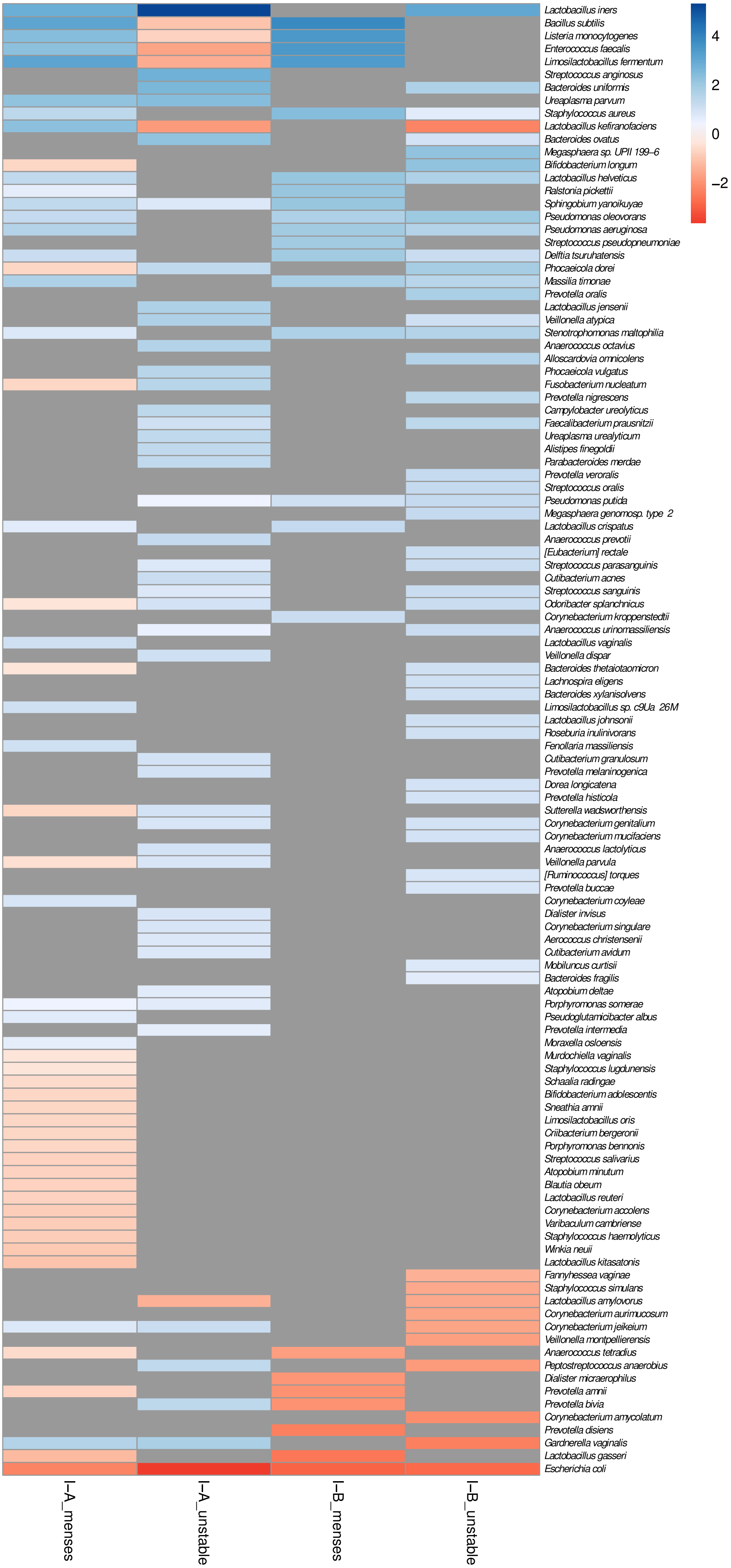
Lactobacillus iners
Bacillus subtilis
Listeria monocytogenes
Enterococcus faecalis
Limosilactobacillus fermentum
Streptococcus anginosus
Bacteroides uniformis
Ureaplasma parvum
Staphylococcus aureus
Lactobacillus kefiranofaciens
Bacteroides ovatus
Megasphaera sp. UPII 199–6
Bifidobacterium longum
Lactobacillus helveticus
Ralstonia pickettii
Sphingobium yanoikuyae
Pseudomonas oleovorans
Pseudomonas aeruginosa
Streptococcus pseudopneumoniae
Delftia tsuruhatensis
Phocaeicola dorei
Massilia timonae
Prevotella oralis
Lactobacillus jensenii
Veillonella atypica
Stenotrophomonas maltophilia
Anaerococcus octavius
Alloscardovia omnicolens
Phocaeicola vulgatus
Fusobacterium nucleatum
Prevotella nigrescens
Campylobacter ureolyticus
Faecalibacterium prausnitzii
Ureaplasma urealyticum
Alistipes finegoldii
Parabacteroides merdae
Prevotella veroralis
Streptococcus oralis
Pseudomonas putida
Megasphaera genomosp. type 2
Lactobacillus crispatus
Anaerococcus prevotii
[Eubacterium] rectale
Streptococcus parasanguinis
Cutibacterium acnes
Streptococcus sanguinis
Odoribacter splanchnicus
Corynebacterium kroppenstedtii
Anaerococcus urinomassiliensis
Lactobacillus vaginalis
Veillonella dispar
Bacteroides thetaiotaomicron
Lachnospira eligens
Bacteroides xylanisolvens
Limosilactobacillus sp. c9Ua 26M
Lactobacillus johnsonii
Roseburia inulinivorans
Fenollaria massiliensis
Cutibacterium granulosum
Prevotella melaninogenica
Dorea longicatena
Prevotella histicola
Sutterella wadsworthensis
Corynebacterium genitalium
Corynebacterium mucifaciens
Anaerococcus lactolyticus
Veillonella parvula
[Ruminococcus] torques
Prevotella buccae
Corynebacterium coyleae
Dialister invisus
Corynebacterium singulare
Aerococcus christensenii
Cutibacterium avidum
Mobiluncus curtisii
Bacteroides fragilis
Atopobium deltae
Porphyromonas somerae
Pseudoglutamicibacter albus
Prevotella intermedia
Moraxella osloensis
Murdochiella vaginalis
Staphylococcus lugdunensis
Schaalia radingae
Bifidobacterium adolescentis
Sneathia amnii
Limosilactobacillus oris
Criibacterium bergeronii
Porphyromonas bennonis
Streptococcus salivarius
Atopobium minutum
Blautia obeum
Lactobacillus reuteri
Corynebacterium accolens
Varibaculum cambriense
Staphylococcus haemolyticus
Winkia neuii
Lactobacillus kitasatonis
Fannyhessea vaginae
Staphylococcus simulans
Lactobacillus amylovorus
Corynebacterium aurimucosum
Corynebacterium jeikeium
Veillonella montpellierensis
Anaerococcus tetradius
Peptostreptococcus anaerobius
Dialister micraerophilus
Prevotella amnii
Prevotella bivia
Corynebacterium amycolatum
Prevotella disiens
Gardnerella vaginalis
Lactobacillus gasseri
Escherichia coli
4
2
0
−2
I−A_menses
I−A_unstable
I−B_menses
I−B_unstable

Supplementary figure 5: Log-fold change of bacterial species in samples from CST-III

Samples in CST-IIIA and CST-IIIB from menses-related dysbiotic or unstable individuals were compared with constant dysbiotic individuals. The heatmap shows the log-fold change of all significant differences. White fields represent no significant change.

This figure occupies a single page.

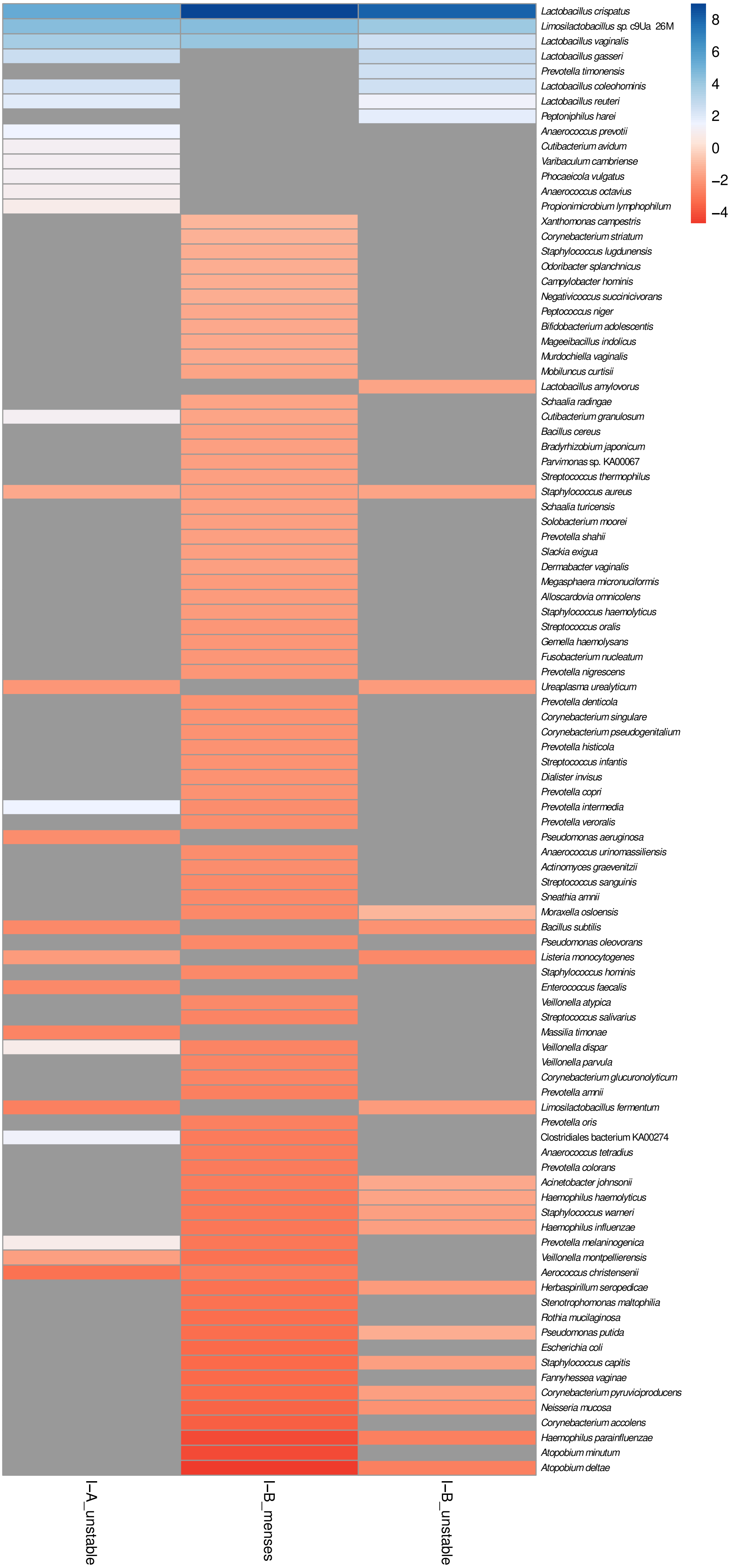
Lactobacillus crispatus
Limosilactobacillus sp. c9Ua 26M
Lactobacillus vaginalis
Lactobacillus gasseri
Prevotella timonensis
Lactobacillus coleohominis
Lactobacillus reuteri
Peptoniphilus harei
Anaerococcus prevotii
Cutibacterium avidum
Varibaculum cambriense
Phocaeicola vulgatus
Anaerococcus octavius
Propionimicrobium lymphophilum
Xanthomonas campestris
Corynebacterium striatum
Staphylococcus lugdunensis
Odoribacter splanchnicus
Campylobacter hominis
Negativicoccus succinicivorans
Peptococcus niger
Bifidobacterium adolescentis
Mageeibacillus indolicus
Murdochiella vaginalis
Mobiluncus curtisii
Lactobacillus amylovorus
Schaalia radingae
Cutibacterium granulosum
Bacillus cereus
Bradyrhizobium japonicum
Parvimonas sp. KA00067
Streptococcus thermophilus
Staphylococcus aureus
Schaalia turicensis
Solobacterium moorei
Prevotella shahii
Slackia exigua
Dermabacter vaginalis
Megasphaera micronuciformis
Alloscardovia omnicolens
Staphylococcus haemolyticus
Streptococcus oralis
Gemella haemolysans
Fusobacterium nucleatum
Prevotella nigrescens
Ureaplasma urealyticum
Prevotella denticola
Corynebacterium singulare
Corynebacterium pseudogenitalium
Prevotella histicola
Streptococcus infantis
Dialister invisus
Prevotella copri
Prevotella intermedia
Prevotella veroralis
Pseudomonas aeruginosa
Anaerococcus urinomassiliensis
Actinomyces graevenitzii
Streptococcus sanguinis
Sneathia amnii
Moraxella osloensis
Bacillus subtilis
Pseudomonas oleovorans
Listeria monocytogenes
Staphylococcus hominis
Enterococcus faecalis
Veillonella atypica
Streptococcus salivarius
Massilia timonae
Veillonella dispar
Veillonella parvula
Corynebacterium glucuronolyticum
Prevotella amnii
Limosilactobacillus fermentum
Prevotella oris
Clostridiales bacterium KA00274
Anaerococcus tetradius
Prevotella colorans
Acinetobacter johnsonii
Haemophilus haemolyticus
Staphylococcus warneri
Haemophilus influenzae
Prevotella melaninogenica
Veillonella montpellierensis
Aerococcus christensenii
Herbaspirillum seropedicae
Stenotrophomonas maltophilia
Rothia mucilaginosa
Pseudomonas putida
Escherichia coli
Staphylococcus capitis
Fannyhessea vaginae
Corynebacterium pyruviciproducens
Neisseria mucosa
Corynebacterium accolens
Haemophilus parainfluenzae
Atopobium minutum
Atopobium deltae
8
6
4
2
0
−2
−4
I–A_unstable
I–B_menses
I–B_unstable

Supplementary figure 6: Volcano plots for the vaginal community dynamics compared to either constant eubiotic or constant dysbiotic

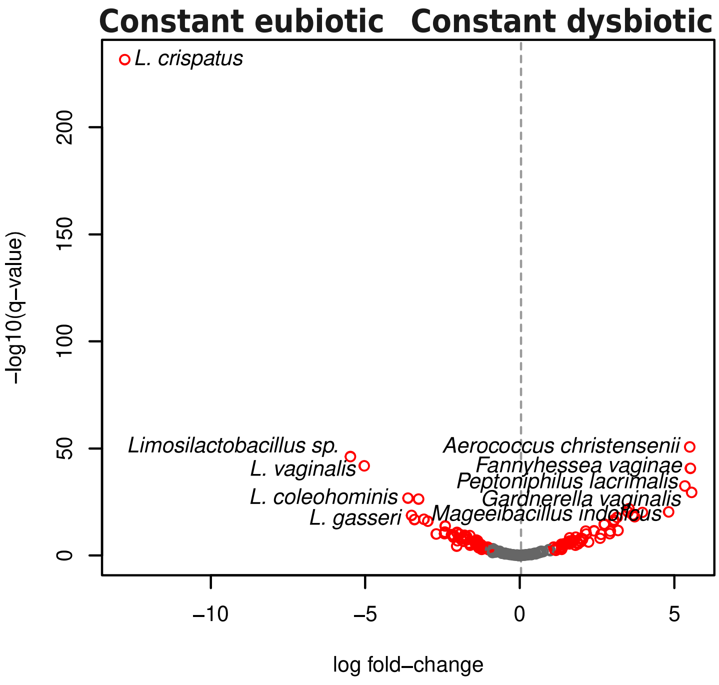

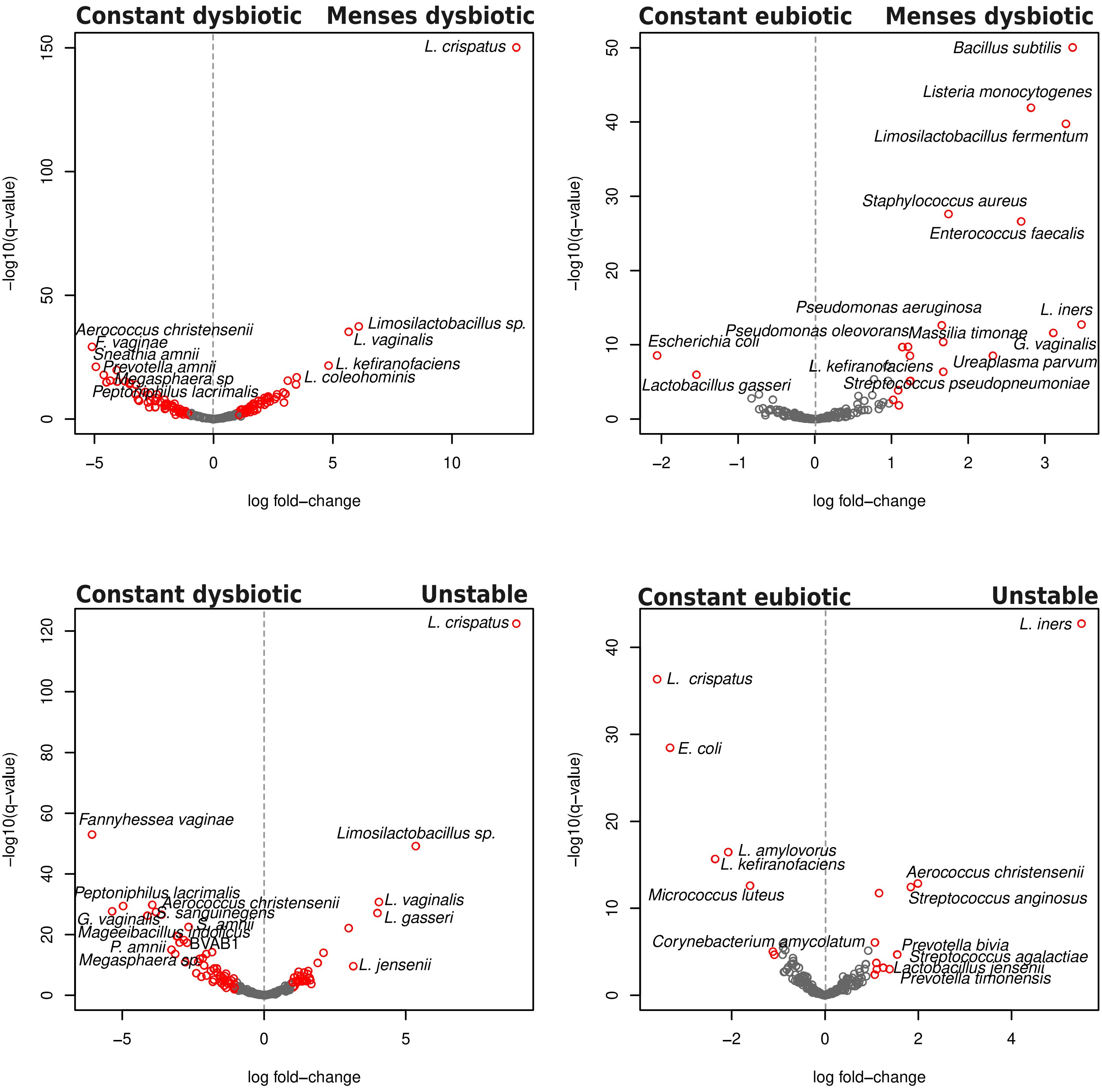

Supplementary figure 7: Histograms showing gene cluster prevalence in nine relevant pangenomes
For each species, the prevalence (number of genomes containing each gene cluster) of each gene cluster is shown as a histogram. Gene clusters present in most or all genomes are considered “core”, while those in one or very few genomes can be considered “cloud”. The “shell” genomes, present in many, but not all genomes, are less frequent

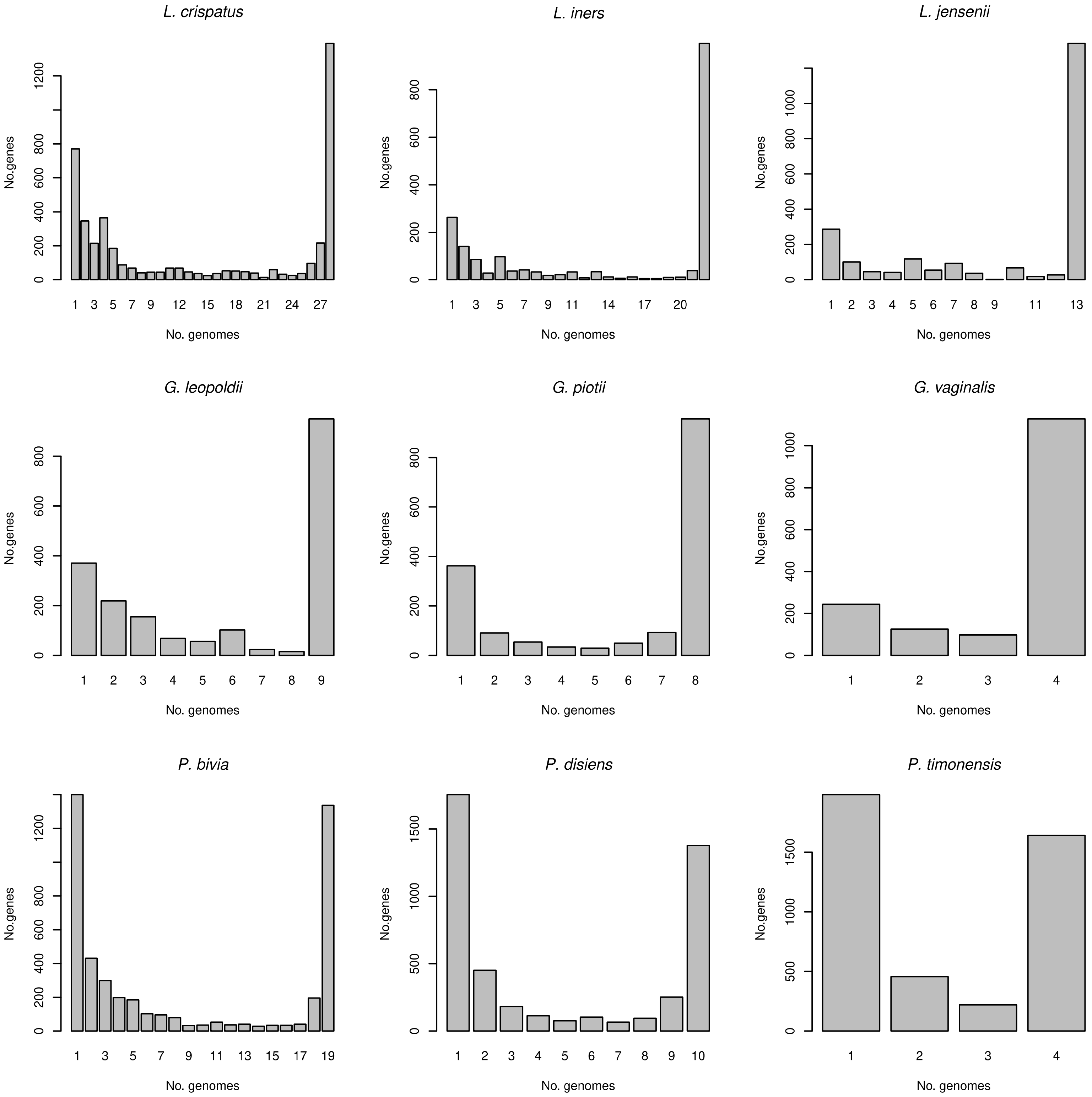

Supplementary figure 8: Phylogenomic analysis of Lactobacillus genomes
Phylogenomic analysis of all detected Lactobacillus species does not find a correlation between the womens' vaginal community dynamics and the observed phylogeny. The presence of a gene is represented in dark blue and its absence in light blue. Blue: constant eubiotic. Red: constant dysbiotic. Yellow: unstable. Green: menses-related dysbiotic.

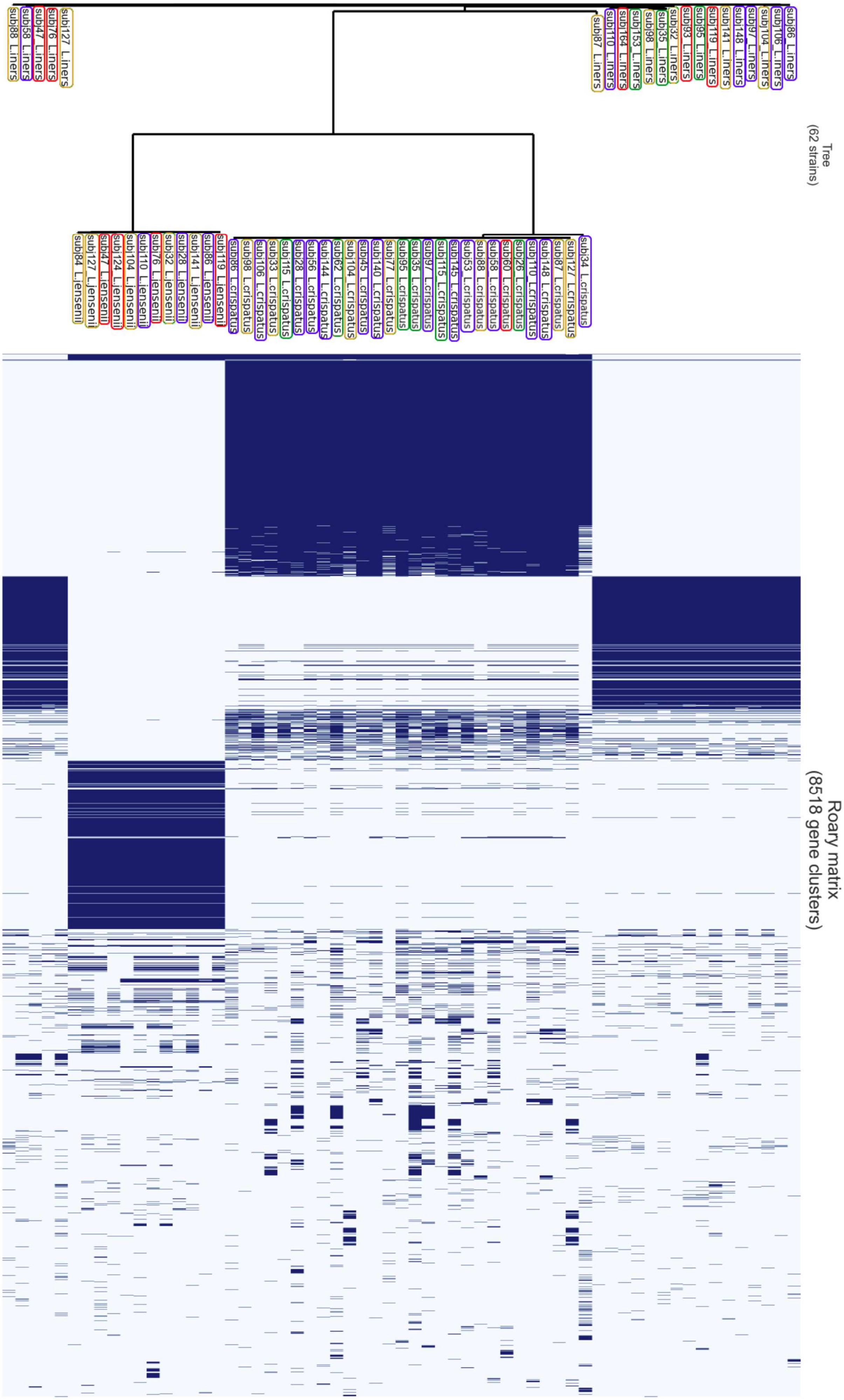

Supplementary figure 9: Phylogenomic analysis of Prevotella genomes

Phylogenomic analysis of all detected Prevotella species does not find a correlation between the womens' vaginal community dynamics and the observed phylogeny. The presence of a gene is represented in dark blue and its absence in light blue.
Blue: constant eubiotic. Red: constant dysbiotic.
Yellow: unstable. Green: menses-related dysbiotic.

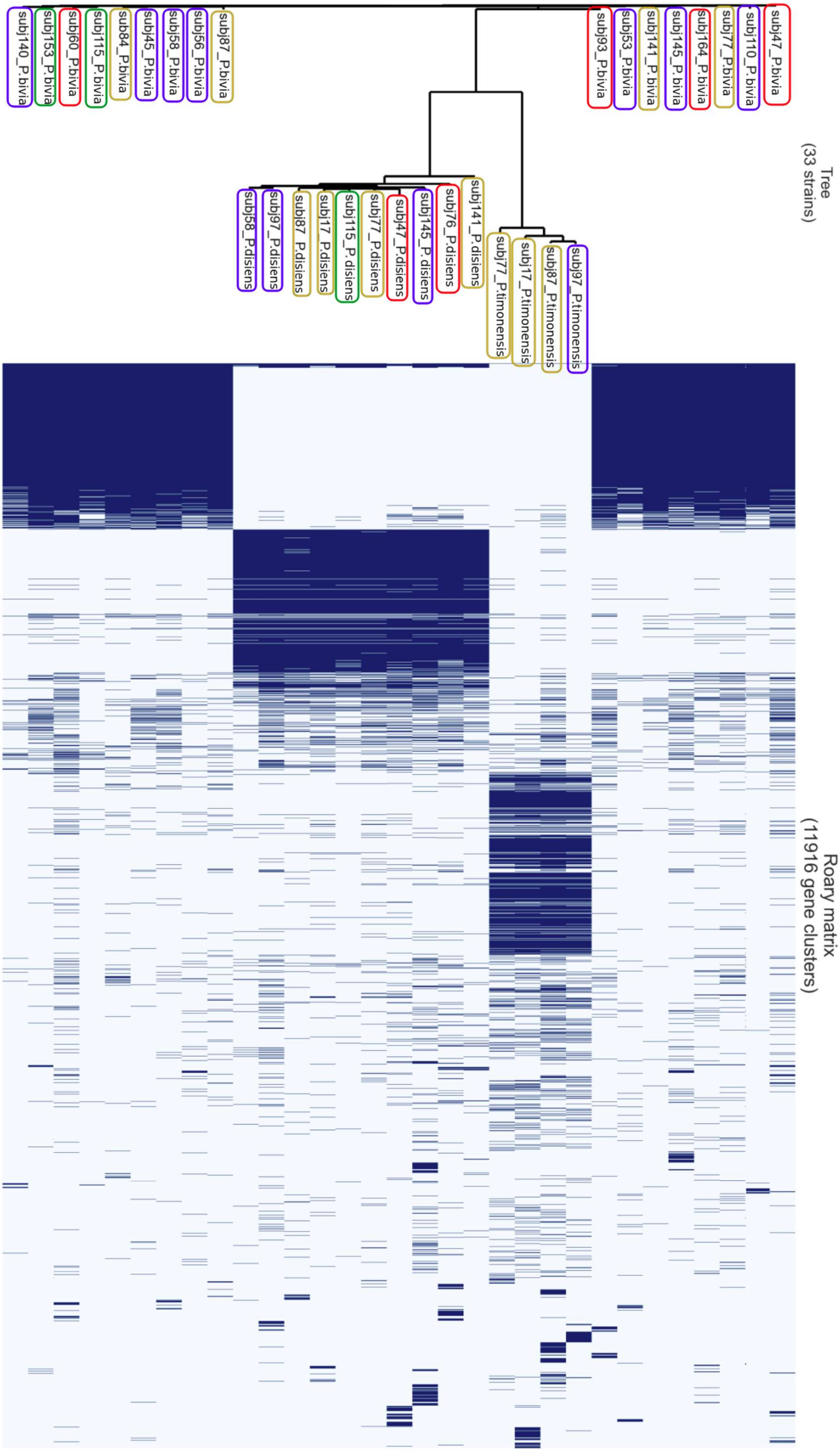

Supplement: Supplementary file 2 — Additional file 1: Supplementary Figure S1. Bacterial and viral profiles for each sample over one menstrual cycle. Each participant’s bacterial and viral profile are depicted as area plots. Sexual intercourse is overlaid as blue dots and vaginal bleedings as red dots. Log10 of the ratio of viral to bacterial reads is shown as a black line over the viral profiles, for time-series with sufficient data (> 5 samples with detectable phages). Missing data is omitted. Next to each taxonomic profile is an ordination showing all samples in the study as gray circles, and the samples for the relevant participant as numbers, following the days of their menstrual cycle. Days with vaginal bleedings are shown in red, days with sexual intercourse in blue and days with both events in purple. Supplementary Figure S2. CST distribution and time-series dynamics for the 16S samples. CSTs are shown as colored dots as per the legend in the second part. The outline of each box depicts the assignment to vaginal community dynamics. Missing samples are omitted. Bleedings are marked as red dots. Blue: constant eubiotic. Green: menses-related dysbiotic. Yellow: unstable. Red: constant dysbiotic. Supplementary Figure S3. CST distribution and time-series dynamics for the shotgun samples. CSTs are marked as colored dots above the taxonomic profiles as per the legend. The outline of each box depicts its dynamic group. Bleedings are marked as light red dots. Missing samples are omitted. Blue: constant eubiotic. Green: menses-related dysbiotic. Yellow: Unstable. Red: constant dysbiotic. Supplementary Figure S4. Log-fold change of bacterial species in samples from CST-I. Samples in CST-IA and CST-IB from menses-related dysbiotic or unstable individuals were compared to constant eubiotic individuals. The heatmap shows the log-fold change of all significant differences. Gray fields represent no significant change. Supplementary Figure S5. Log-fold change of bacterial species in samples from CST-III. Samp [file 40168_2024_1870_MOESM1_ESM.pdf]
